# Supplementary material for: Examining the effectiveness of parental strategies to overcome bedwetting: an observational cohort study
Source: BMJ Open. 2017 Jul 13;7(7):e016749. doi: 10.1136/bmjopen-2017-016749 (PMC5541498; doi:10.1136/bmjopen-2017-016749)
Supplement: Supplementary data [file bmjopen-2017-016749supp001.pdf]

Supplementary online material

Rationale for applying propensity score methods

The main purpose of propensity score-based methods is to draw causal inferences about effects of treatments from observational data. In order to do this certain assumptions are required. In observational data the process of selection to the 'treated' and 'non-treated' groups is not random. Direct comparisons of the outcomes in both groups are likely to be biased if 'treated' and 'non-treated' groups differ systematically on certain variables (both measured and unmeasured) that may also be associated with the outcome. It is, therefore, not possible to establish to what extent the observed results are effects of the 'treatment' versus other important variables that remained uncontrolled in the process of selection of cases into the groups. Dealing with this problem of confounding is a central problem of studies based on observational data. As Rosenabum (2005)[1] states the key issues in such studies are to remove 'overt biases' (related to measured/observed confounders) and to address somehow uncertainty about those hidden (unmeasured confounders).

Multivariable regression and, more recently, propensity score-based methods can be used to minimise effects of confounders when estimating treatment effects using observational data. These methods adjust the crude results to take into account the non-random mechanism of selection of cases to the 'treated' versus 'non-treated' groups by controlling for differences between both groups. Propensity score-based methods are based on the assumption that differences between 'treated' and 'non-treated' groups can be explained in terms of observable variables and bias should, therefore, be minimised by ensuring similarity of both groups on these variables. This is achieved using propensity score-based methods either by matching each case from the 'treated' group to at least one similar case from the 'non-treated' group or by re-weighting both groups in order to construct a synthetic sample in which both groups are similar with respect to the observed confounders. Similarity of the groups is assessed in terms of the probability of receiving the 'treatment' as estimated from the observed variables of the cases. This probability, called the 'propensity score', is further used to create/or reshape the sample in which distributions of variables of study participants are similar in both groups. If this condition is fulfilled then treated and untreated groups are balanced on measured confounders. However, in order to compare their results directly it is also important to assume that all of the important confounders were measured and included in the analysis. It is only under these conditions that estimates of causal effects are assumed to be unbiased.

Application of propensity score-based methods in the current study

We used propensity score-based methods to assess the effectiveness of parental strategies aimed at preventing bedwetting. Within our study, 'treated' refers to those children receiving each parental strategy at 7 ½ years and 'non-treated' refers to those not receiving the strategy.
The propensity score-based analysis involves three general steps: (1) constructing the propensity scores; (2) balancing the sample based on their propensity scores; and (3) estimating effects of the treatment (parental strategy). At each of these steps in the analysis, decisions need to be made that influence the results and final conclusions. Below, we present the key decisions we made in conducting the propensity score-based analysis:

a) Average treatment effect versus average treatment effect on treated:

One of the most crucial decisions in the analysis concerns the indicator of the impact of the treatment. These are: average treatment effect (ATE) or average treatment effect on treated (ATT). They are both rooted within the counterfactual framework, which assumes that each participant included in the study has two potential outcomes i.e. the outcome when treatment was applied and the outcome when treatment was not applied. In reality, only one of these outcomes is truly observed, and the other is unobserved (counterfactual). The ATE is defined as the difference between averages of these two potential outcomes at the population level. The ATT is the difference between potential outcomes calculated for the treated group.
The aim of the analysis was to estimate the effect of each parental strategy aimed at preventing bedwetting at 7½ years as a difference in risk of bedwetting at 9½ years between those children receiving the strategy (treated) versus those children not receiving the strategy (non-treated). We assessed the effectiveness of each parental strategy by means of the average treatment effect for the treated (ATT) because we were interested in the average effect of treatment on those children who ultimately received the treatment rather than in the estimation of the average treatment effect at the population level (ATE).

b) Selection of propensity score based method:
The four most reported methods for using propensity scores in the estimation of the effect of treatment are propensity score matching, stratification on propensity score, inverse probability weighting and covariate adjustment using propensity scores. Austin (2011)[2] provides a detailed discussion of the strengths and limitations of these methods. In brief, the method based on inverse probability weighting is favoured, whilst the other methods were found to be more prone to bias in Monte Carlo studies. We used the inverse probability of treatment weighting (IPTW) where the synthetic balanced sample is constructed (participant variables are independent on the assignment to treated/non-treated groups). We estimated the propensity scores by logistic regression.

c) *Selection of baseline variables to the propensity score model*:
There are at least four possible strategies for variable selection and there is lack of consensus in the literature on which strategy is the most appropriate.[2] The available strategies are: (1) propensity model should include all measured, theoretically relevant variables; (2) model should include variables that are associated with the treatment; (3) model should include variables that are associated with the outcome; (4) model should include only those variables that are associated both with treatment and outcome. The final strategy seems to be the favoured approach as regards theoretical arguments and results of simulation studies.[2] Therefore, we chose this model including only those variables that are associated both with treatment (parental strategy) and outcome (bedwetting at 9 ½ years). We used a two-stage procedure to derive the list of baseline variables to include in the model. Firstly, we conducted two logistic regression analyses in the first completed dataset (m=1). In the first regression model, the outcome variable was predicted from all theoretically relevant variables relating to children and their family (see table S1 for the list of all variables included). In the second regression model, each parental strategy was the outcome variable. Secondly, we applied a threshold for selection of variables to the propensity score model - only the variables that had associations (expressed in terms of odds ratios) with the outcome variables in both models of <0.90 or >1.20.[3] We enriched the propensity score models by including the parental strategies that were highly correlated with the

specific strategy considered in each model. The alternative strategy was included in the model for a given strategy if the tetrachoric correlation was >0.45.

d) *Diagnostics:*
Propensity score based diagnostic methods are designed to examine whether the distributions of the baseline variables are the same/or similar enough in treated and non-treated groups. There are several specific diagnostic procedures that are recommended. Among them the most frequently used are those examining the standardized differences between means of baseline variables after matching/weighting and those comparing their variances. Before analysing the treatment effects, we assessed the adequacy of the propensity score model by checking the overlap in propensity scores. We based our diagnostic checks on the examination of differences between means of baseline variables in the weighted sample. Categorical variables with more than two categories were examined using sets of binary variables. Following the literature we assumed that absolute standardized mean difference < 0.1 indicates negligible differences between groups. Since the model computations were conducted on 50 datasets obtained from the multiple imputation procedure (see section below for details) we performed the diagnostic checks in 5 randomly selected datasets (m=1,4,5,37,50). See tables S2 to S7 and figures S1 to S6 for the results of the diagnostic checks to assess the adequacy of the propensity score models.

e) Dealing with missing data and estimating the treatment effects:
The magnitude of missing data per variable is presented in table S1. To deal with the missing data, we used multiple imputation by chained equations within ICE (Imputation by Chained Equations) STATA package v.14. We generated 50 imputed datasets using 10 cycles of regression switching. We used a range of auxiliary data, which does not itself form part of the analytical model. The criteria of variable selection to the imputation model for each variable included: (i) theoretical and empirical evidence from prior published research; (ii) data driven: variable was selected if in the current dataset it was strongly associated with observed values of the imputed variable or missingness on that variable; (iii) additionally, the imputation models for every variable included the parental strategies and bedwetting at 9½ years.
Conducting the analysis based on propensity scores on multiply imputed datasets required a decision about the exact routine by which the treatment effects are calculated. There are two possibilities: (1) first estimate the propensity score in each of 50 completed datasets and average the propensity score for every subject across all datasets and then estimate treatment effects based on those aggregated propensity scores; (2) estimate propensity scores and treatment effects separately in every completed dataset and then aggregate the treatment effects obtained according to Rubin's rules. Published simulation studies suggest that first strategy provides biased results[4] therefore, we used the second strategy.
To our best knowledge there is no STATA package that deals with treatment effects in the context of multiple imputation, so we developed our own syntax using loops to estimate treatment effects and standard errors separately within each file and exported the results to Excel. At the next stage, we aggregated the exported results by implementing Rubin's rules for estimating the effects and standard errors from the multiply imputed datasets. [5] For the estimation *teffects ipw* we used the STATA built-in command and for the diagnostic procedures we used *tebalance summarize*. Models for medication and bedwetting alarm were not possible to estimate due to a very low number of children receiving these interventions.

We conducted diagnostics checking the plausibility of the imputation process by means of the *midiagplots* STATA command after datasets from the *ICE* package were successfully exported. Due to the large number of generated datasets, the diagnostics were based only on a subset of five randomly selected datasets (m=1,4,5,37,50) obtained from the MI procedure. The diagnostics were conducted with the assumption that the missing data mechanism was MAR. We compared the distributions of observed, imputed and completed values of each variable. The diagnostics revealed that they did not differ greatly which suggested that the imputation model was correctly specified. (The figures and tables including those diagnostic statistics are available on request).

Caveats of using propensity score based methods

Propensity score based methods are a group of methods used to estimate treatment effects from observational data by balancing treatment and non-treatment groups on the set of measured confounders (**X**). As Rosenbaum and Rubin (1983)[6] showed that this can be achieved by using propensity scores instead of all combinations of variables from **X**.

All propensity score methods rely on several assumptions of which the most crucial is the one known as the conditional independence assumption (CIA). It consists of two parts: (a) the potential result of being treated (Y_1) and the potential result of remaining untreated (Y_0**)** are independent of the treatment group (G), after controlling for a vector of measured confounders **(X):** $Y_{\mathbf{0},} Y_1 \perp G|\boldsymbol{X}$ and (b) every case, (with any characteristic on vector **X**) has a positive chance of being included in either treatment group, which is $0<P(G=1|\mathbf{X})<1$.

Part (a) of CIA assumes that there are no unmeasured confounders. If vector **X** does not include all important confounders (those related both to the outcome and the treatment) (a) is not satisfied and causal inferences might be biased. Unfortunately, this is the untestable part of CIA and needs to be assumed. Part (b) of CIA implies that there must be an overlap between treated and non-treated groups with regards to their characteristics on vector **X** which means that any person with any characteristic on **X** within treatment group will have their counterpart in non treatment group[1]. In contrary to (a), part (b) of CIA concerning the overlap of treated and non-treated is testable and can be made by comparing distributions of propensity scores for both groups. We have done this by graphical examination of propensity scores estimated for both treatment groups (see figures S2-S7). This assumption was fulfilled to a satisfactory extent since the differences we observed in the distributions of propensity scores in treated and non-treated groups were minor.

It should be also added here that since in the current paper we relied on ATT, both parts of CIA are adopted in their weaker, less stringent, versions, This, weaker version of CIA requires for (a) only $Y_{\mathbf{0},} \perp G|\boldsymbol{X}$ to hold (which remains untestable) and for (b) that cases from the treatment group will have their counterparts in the untreated group (but the overlap in the opposite direction is not necessary now). As shown in figures S2-S7 this milder version of the assumption generally held in the data.

After applying methods using propensity scores the assessment of balance achieved between both compared groups is the final diagnostic step. It is also notable that the assessment of balance obtained as a result of applying inverse propensity score weighting returned satisfactory results.

References

1. Rosenbaum PR. Observational Study. In: Everitt BS, Howell DC, eds. Encyclopedia of Statistics in Behavioural Science Volume 3. Chichester: John Wiley & Sons, Ltd. 2005: 1451-1462.
2. Austin PC. An introduction to propensity score methods for reducing the effects of confounding in observational studies. Multivariate *Behav Res* 2011; 46(3): 399–424.
3. Monson RR. Occupational Epidemiology. 2nd Edition. CRC Press, Boca Raton, London, New York, Washington, DC, 1990.
4. Mitra R, Reiter JP. A comparison of two methods of estimating propensity scores after multiple imputation. *Stat Methods Med Res* 2016; 25(1): 188-204.
5. White IR, Royston P, Wood AM. Multiple imputation using chained equations: Issues and guidance for practice. *Statistics in Medicine* 2011; 30(4): 377-399.
6. Rosenbaum PR, Rubin DB, The central role of the propensity score in observational studies for causal effects. *Biometrika* 1983; 70: 41-55.

Table S1. Model variables for multiple imputation (MI) with numbers of missing cases per variable (n =1258).

	**MODEL VARIABLES**	**Number of missing/ imputed cases by MI**	**Imputation model**
	Confounders		
Variable number:	Child and family variables:		
1	Gender	0	none
2	Developmental level at 18 months (m)[1]	100	linear regression
3	Temperament - Activity at 24 m[2]	129	logistic
4	Temperament - Adaptability at 24 m[2]	135	logistic
5	Temperament - Intensity at 24 m[2]	170	logistic
6	Temperament - Mood at 24 m[2]	132	logistic
7	Temperament - Persistence at 24 m[2]	140	logistic
8	Prosocial behaviour score at 81m[3]	146	logistic
9	Hyperactivity score at 81m[3]	146	logistic
10	Emotional symptoms score at 81m[3]	145	logistic
11	Conduct problems score at 81 m[3]	144	logistic
12	Peer problems score at 81 m[3]	144	logistic
13	Total behavioral problems score at 81 m[3]	146	logistic
14	EAS temperament -Emotionality 69 m[4]	157	logistic
15	EAS temperament -Activity 69 m[4]	149	logistic
16	EAS temperament -Shyness 69 m[4]	149	logistic
17	EAS temperament -Sociability 69 m[4]	179	logistic
18	Toilet training has not been started by 24 m (parental report)	562	logistic

19	Verbal IQ 8 years (y)[5]	296	logistic
20	Performance IQ 8 y[5]	297	logistic
21	Full scale IQ 8 y[5]	303	logistic
22	Social communication 7.5 y[6]	21	logistic
23	Stressful life events (child related) 42 m[7]	137	logistic
24	Sleep problems at 42 m (parental report)	83	logistic
25	Child's general health at 81 m (parental report)	214	logistic
	Problems with urinating at 7.5 y (parental report)		
26	Frequency child shows signs of needing to urinate	<5	logistic
27	Going to toilet without reminder	<5	logistic
28	Child needs to dash to toilet to urinate	13	logistic
29	Bedwetting frequency (>= twice a week)	0	none
30	Daytime wetting	<5	logistic
	Problems with soiling at 7.5 y (parental report)		
31	Daytime soiling	<5	logistic
32	Nighttime soiling	7	logistic
	Socioeconomic indicators (parental report)		
33	Family size (3 children or more)	22	logistic
34	Crowding index at 85 m	143	logistic
35	Home ownership at 85 m	146	logistic
36	Difficulty affording elementary goods for mother at 85 m	141	logistic
37	Difficulty affording elementary goods for child at 85 m	186	logistic
38	No car at 33m	126	logistic
39	Social class at 3 y11 m - based on mother's occupation	659	logistic
40	Social class at 3 y11 m - based on father/partner's occupation	340	logistic

	Mother: (maternal report)		
42	Maternal education (reported in antenatal period)	32	logistic
43	Early parenthood	0	
44	Marital status of mother	139	logistic
45	Mother has paid work outside home since child born (85 m)	124	logistic
46	Mother has help looking after child (85 m)	168	logistic
47	Mother has help with housework (85 m)	165	logistic
48	Mother feels she is getting enough sleep (85 m)	144	logistic
49	Mother spends enough time with child (85 m)	158	logistic
50	Mother had stressful life events since child's 5th birthday	128	logistic
51	Mother's general health (73 m)	132	logistic
52	Maternal psychopathology – depression (73 m)	135	logistic
53	Maternal psychopathology - anxiety at (73 m)	142	logistic
54	Mother bedwetting history	41	logistic
60	Mother daytime wetting history	41	logistic
61	Parenting style nurturing	113	logistic
62	Parenting style discipline	95	logistic
63	Toilet training at 15 m[8]	73	logistic
64	Toilet training at 24 m[8]	109	logistic
65	Rules about smoking in house	141	logistic
66	Does anyone smoke in the house?	164	logistic
65	Father figure is natural father of child	154	logistic
	PSM - outcome variable:		
O1	Bedwetting at 9.5 y	213	logistic
	PSM - treatment (strategy) variables:		
S1	Lifting	0	
S2	Restricting drinks	0	

S3	Daytime toilet trips	0
S4	Rewarding	0
S5	Medication	0
S6	Alarm prevention	0
S7	Showing displeasure	0
S8	Protection pants	0

1. Maternal rating of developmental level was assessed at 18 months using a questionnaire developed by ALSPAC including items from the Denver Developmental Screening Test (Frankenburg et al. Pediatrics 1992; 89(1):91-97) and comprising four domains of development (Cronbach's alphas: fine motor=0.679; gross motor= 0.689; communication= 0.752 and social skills= 0.624). Scores on each domain were adjusted for age in weeks, standardized (using a linear regression model and extracting the residuals) and reversed where appropriate so that high values on all scores reflected a lower level of development. We adjusted for a total development score derived from the sum of the scores on each domain.
2. Mothers completed the Toddler Temperament Scale (TTS: Fullard, et al. Journal of Pediatric Psychology 1984; 9:205-216) when the study children were 24 months. The TTS comprises statements describing specific behaviours and mothers were asked to rate how often their child behaves in that way on a scale ranging from 1 (almost never) to 6 (almost always). The scale comprises nine temperament traits, but we restricted our analysis to five traits we found were associated with bedwetting in an earlier study (Joinson et al. 2009. Longitudinal and Life Course Studies 1:73-94): *activity* (□□= 0.593), *adaptability* (□□= 0.637), *intensity* (□□= 0.570), *mood* (□□= 0.672) and *persistence* (□□= 0.711).
3. Psychological problems were assessed when the child was aged 81 months using the Revised Rutter Parent Scale for Preschool Children, which is an extension of the Rutter behaviour scale (Elander & Rutter. Journal of Methods in Psychiatric Research 1996; 6:63–78). The questionnaire comprises 43 statements describing behaviours and mothers are asked to rate the extent to which each statement describes their child on a scale comprising the options 1 (certainly true), 2 (sometimes true) and 3 (not true). Responses were aggregated to create scores in five domains: *emotional difficulties*, *conduct difficulties*, *hyperactivity* and *prosocial behaviour*. High levels of problems are indicated by high scores on emotional difficulties, conduct difficulties and hyperactivity and low scores on the prosocial behaviour scale.
4. The Emotionality Activity Sociability (EAS) Questionnaire (Buss & Plomin. Temperament: Early developing personality traits. Hillsdale, NJ: Erlbaum. 1984) was administered when study children were 69 months. The questionnaire comprises 20 statements about behaviours and mothers rated the extent to which each statement describes their child on a scale ranging from 1 (not at all like) to 5 (exactly like). Scores on these items are combined to form four subscales (each comprising five items): *emotionality*, *activity level*, *shyness* and *sociability*.
5. IQ: Wechsler D. Manual for the Wechsler Intelligence Scale for Children. 3rd ed. San Antonio, TX: Psychological

Corporation; 1991.

6. When children were 7.5 years mothers completed the 12-item Social Communication Disorder Checklist (Skuse et al. Br J Psychiatry 2005;187:568-572). A score of 2 SD or more below the mean was selected as indicating impaired social communication.
7. Stressful life events were measured using a maternally reported questionnaire comprising 42 life events that was derived for ALSPAC using previous inventories as a basis for item selection (Barnet et al. J Psychosom Res 1983: 27, 313–320).
8. Toilet training: Mothers were asked: What do you think about toilet training for your child? Options were: “It is too early to start any toilet training yet”, “I have just started toilet training” and “I have been toilet training for some time”.

Assessing the adequacy of the propensity score models

Table S2(i). Diagnostics summary for **LIFTING**

**N in unweighted dataset: treated = 159; untreated 1,099**	**Model adjusted for child and family variables**					**Model adjusted for child and family variables and other parental strategies**				
	**m=1**	**m=4**	**m=5**	**m=37**	**m=50**	**m=1**	**m=4**	**m=5**	**m=37**	**m=50**
**N of treated in weighted dataset:**	**630**	**628**	**629**	**629**	**629**	**632**	**635**	**632**	**637**	**639**
**N of untreated in weighted dataset:**	**628**	**630**	**629**	**629**	**629**	**626**	**623**	**627**	**621**	**619**

Figure S1. Overlap for models for **LIFTING** including child and family variables

m=1	m=4	m=5	m=37	m=50
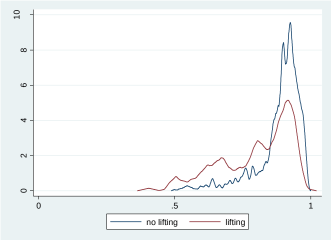	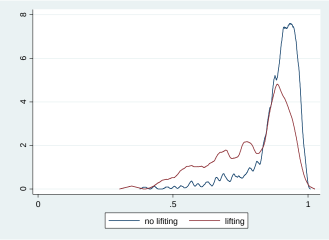	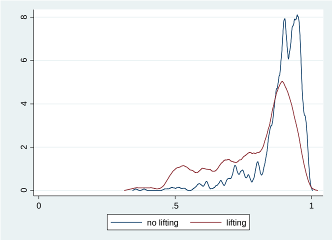	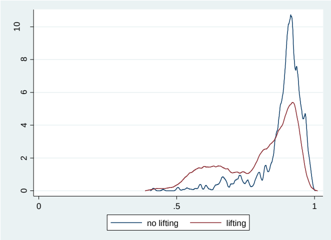	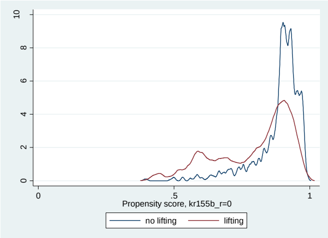
Overlap for models for **LIFTING** including child and family variables & other related parental strategies				
m=1	m=4	m=5	m=37	m=50
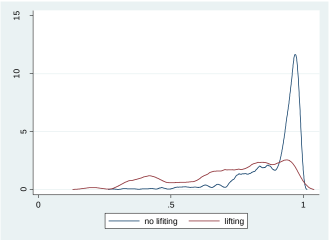	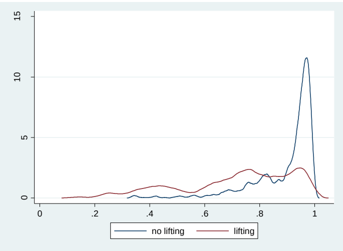	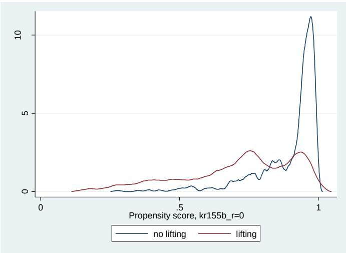	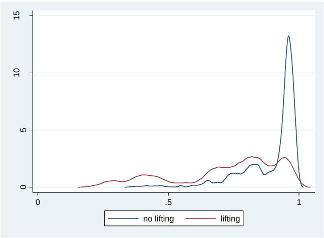	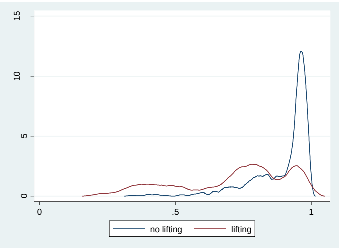

Table S2(ii). Standardized differences on confounders after weighting

**CONFOUNDERS**	**Standardized differences in weighted sample in model adjusted for child and family variables**					**Standardized differences in weighted sample in model adjusted for child and family variables and other parental strategies**				
**Imputed dataset:**	**m=1**	**m=4**	**m=5**	**m=37**	**m=50**	**m=1**	**m=4**	**m=5**	**m=37**	**m=50**
**Child and family variables included as confounders:**										
Temperament - Activity at 24 m										
1. high score >28	.035	.026	.012	.003	.017	.054	.039	.033	.035	.043
Temperament - Adaptability at 24 m										
1. high score >17	-.002	-.009	.006	-.016	.004	.033	.014	.039	.039	.038
Temperament - Mood at 24 m										
1. high score >24	.019	-.002	.018	.017	.011	.073	.071	.063	.064	.048
Conduct problems score at 81 m										
1 abnormal (score >=4 (hyperproblems))	-.011	-.021	-.017	-.032	-.016	-.023	.003	.014	-.016	-.015
Total behavioural problems score at 81 m										
1 abnormal (score >=14 (hyper behavioral problems))	.009	-.004	-.008	.001	.007	.001	.007	.017	.002	.013
EAS temperament - activity at 69 m										
1 abnormal (score >=24 (hyper active))	-.011	.002	-.021	-.011	-.006	.041	.031	.033	.035	.036
EAS temperament -sociability at 69 m										
1 abnormal (score >=22 (antisocial))	.012	.012	.005	.013	.004	.012	.001	-.008	-.002	-.003
Verbal IQ										
1. 80<IQ<=120	.001	.022	-.001	.008	.013	-.026	-.027	-.028	.001	-.014
2. IQ>120	-.006	-.008	-.008	-.003	-.012	.015	.048	.018	-.001	.003
Performance IQ										
1. 80<IQ<=120	-.002	.018	-.001	.009	-.009	.024	.039	.029	.027	.016
2. IQ>120	-.003	-.002	-.001	-.007	-.006	-.028	-.019	-.027	-.057	-.043
Full scale IQ										
1. 80<IQ<=120	.001	.029	.022	.024	.020	-.008	.026	.028	.045	.021

2. IQ>120	.003	-.006	-.016	-.010	-.012	.013	.009	-.026	-.037	-.037
Social cognition										
1.(score>=8)	-.011	-.038	-.033	-.027	-.007	.000	-.036	-.004	.011	.014
Going to toilet without reminder										
1. never	-.006	.000	-.001	-.005	-.012	-.013	.001	.005	-.004	-.011
Bedtime wetting severity										
1. severe	.006	-.001	.007	.000	.017	.043	.041	.051	.061	.085
Daytime soiling										
1. yes	-.024	-.041	-.044	-.052	-.037	-.001	-.029	-.013	-.006	.003
Family size (3 or more children)										
1 # of children >=3	-.013	.005	-.002	.006	-.002	-.082	-.030	-.034	-.031	-.017
Social class - based on mother's occupation										
1. Managerial and technical	.004	-.007	.012	-.009	-.031	.025	.009	-.015	.011	-.018
2. Skilled non-manual	-.010	.004	.010	.011	-.002	-.026	-.024	-.014	.002	-.018
3. Skilled manual	-.019	-.024	-.003	-.029	.010	-.024	-.028	.000	.005	-.020
4. Partly skilled	-.003	-.005	-.022	.003	.019	.024	.037	.036	-.006	.044
5. Unskilled	.017	.019	.020	.014	.021	.037	.013	.029	.014	.036
Social class - based on father/partner's occupation										
1. Managerial and technical	.001	-.003	-.017	.002	-.005	.009	-.003	-.026	.013	.016
2. Skilled non-manual	.000	.012	.014	-.013	-.009	.002	-.002	-.002	-.020	-.043
3. Skilled manual	.003	.019	.024	.021	.030	.009	.023	.054	.0358	.059
4. Partly skilled	-.020	-.032	-.027	-.029	.002	-.044	-.020	-.053	-.044	-.017
5. Unskilled	.010	-.004	.007	.016	-.037	.015	.000	.034	.0199	-.016
Early parenthood										
1. Had child at age <19	.007	.005	.002	-.010	.000	.002	.001	.000	-.021	-.004
Amount of help mother has in looking after child at 85 m										
1. Too little	-.007	.002	.013	-.023	-.015	.030	.008	.023	-.001	.015
Mother state of health 73 m										
1. Mostly well & healthy	.030	.020	.032	.018	.025	.042	-.012	.029	.019	.034

2. Often unwell & hardly ever well	-.022	-.024	-.042	-.023	-.041	-.014	.037	-.034	.0159	-.011
Mother's history of bedwetting										
1. Mother had a problem when older than 5yo	-.009	-.003	-.018	-.011	-.018	-.034	-.020	-.028	-.014	-.023
Toilet training when child is 15 m										
1. Just started toilet training	.023	.002	-.005	.012	.009	.040	.022	.039	.031	.040
2. Toilet training for some time	.017	.016	.019	.015	.016	.051	.021	.019	.026	.018
Rules about smoking in household										
1. Smoking allowed in some rooms	-.021	-.017	-.017	-.010	-.014	-.005	-.014	-.014	.0196	.009
2. Smoking allowed	.004	.013	.014	.011	.010	-.002	.001	.009	.000	-.006
Father figure is natural father of child										
1. No / not living with father figure	-.005	-.003	.008	.021	.024	-.026	-.006	.011	.017	.021
**Related parental strategies variables (those with correlations>.45):**										
Restricting drinks										
1. yes						.000	.015	-.004	.021	.026

Table S3(i). Diagnostics for **RESTRICTING DRINKS**

**N in unweighted dataset: treated = 403; untreated 855**	**Model adjusted for child and family variables**					**Model adjusted for child and family variables and other parental strategies**				
	**m=1**	**m=4**	**m=5**	**m=37**	**m=50**	**m=1**	**m=4**	**m=5**	**m=37**	**m=50**
**N of treated in weighted dataset:**	**631**	**631**	**633**	**631**	**633**	**627**	**630**	**630**	**629**	**629**
**N of untreated in weighted dataset:**	**628**	**627**	**625**	**627**	**625**	**631**	**628**	**628**	**630**	**630**

Figure S2. Overlap for models for **RESTRICTING DRINKS** including child and family variables

<table>
<tr><td>m=1</td><td>m=4</td><td>m=5</td><td>m=37</td><td>m=50</td></tr>
<tr>
<td>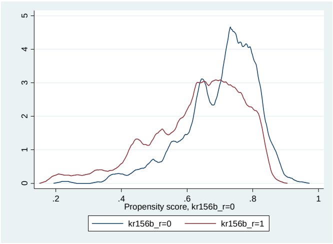</td>
<td>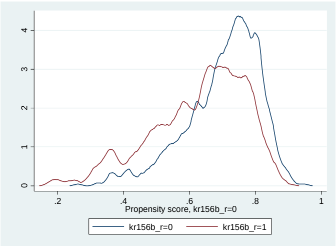</td>
<td>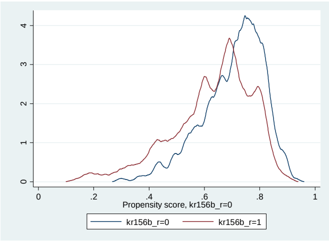</td>
<td>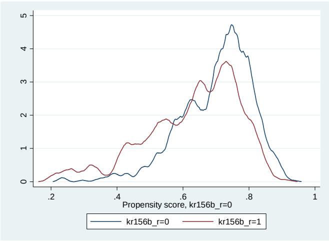</td>
<td>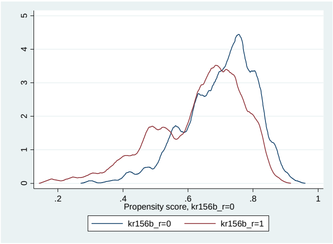</td>
</tr>
<tr><td colspan="5">Overlap for models for RESTRICTING DRINKS including child and family variables & other related parental strategies</td></tr>
<tr><td>m=1</td><td>m=4</td><td>m=5</td><td>m=37</td><td>m=50</td></tr>
<tr>
<td>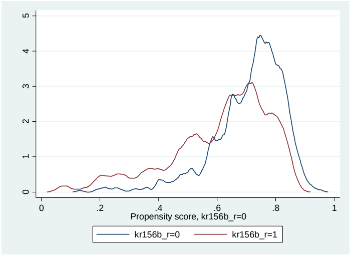</td>
<td>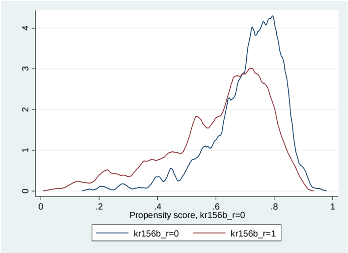</td>
<td>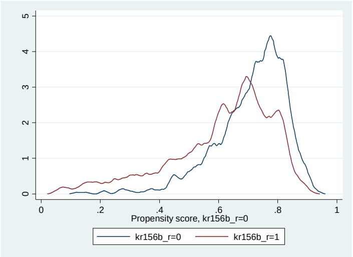</td>
<td>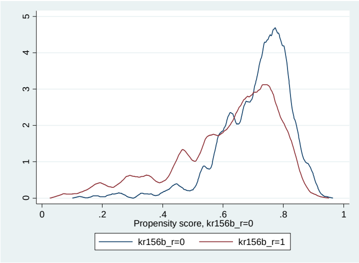</td>
<td>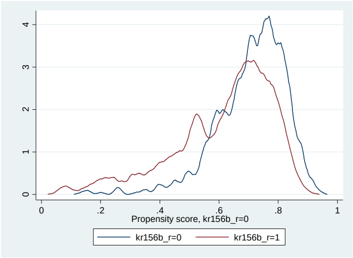</td>
</tr>
</table>

Table S3(ii). Standardized differences on confounders after weighting

**CONFOUNDERS**	**Standardized differences in weighted sample in model adjusted for child and family variables**					**Standardized differences in weighted sample in model adjusted for child and family variables and other parental strategies**				
**Imputed dataset:**	**m=1**	**m=4**	**m=5**	**m=37**	**m=50**	**m=1**	**m=4**	**m=5**	**m=37**	**m=50**
**Child and family variables included as confounders:**										
Gender										
1. Female	*.017*	*.001*	*.010*	*-.004*	*.022*	*.025*	*.016*	*.003*	*.004*	*.027*
Temperament - Adaptability at 24 m										
1. High score >17	*.011*	*.036*	*.036*	*.009*	*.017*	*.020*	*.025*	*.041*	*.014*	*.010*
Emotional symptoms score at 81 m										
1. High score >=4	*-.007*	*.002*	*-.002*	*-.016*	*.006*	*-.108*	*.008*	*-.009*	*-.011*	*.011*
Conduct problems score at 81 m										
1. High score >=4	*.031*	*.041*	*.056*	*.016*	*.057*	*.042*	*.067*	*.068*	*.031*	*.075*
Total behavioral problems at 81 m										
1. High score >=14	*.014*	*.019*	*.031*	*.012*	*.027*	*.028*	*.038*	*.046*	*.030*	*.046*
EAS temperament -sociability at 69 m										
1 abnormal (score >=22 (antisocial))	*.029*	*.039*	*.034*	*.017*	*.020*	*.036*	*.056*	*.037*	*.031*	*.046*
Verbal IQ at 8 y										
1. 80<IQ<=120	*.019*	*.003*	*-.013*	*-.012*	*.015*	*-.001*	*-.014*	*-.023*	*-.024*	*-.012*
2. IQ>120	*-.014*	*-.014*	*-.001*	*.002*	*-.030*	*-.002*	*-.006*	*.002*	*.008*	*-.007*
Performance IQ at 8 y										
1. 80<IQ<=120	*-.022*	*.003*	*.000*	*.027*	*.009*	*-.045*	*-.019*	*-.019*	*-.003*	*-.013*
2. IQ>120	*-.005*	*-.017*	*-.011*	*.008*	*-.009*	*.018*	*-.005*	*.012*	*.033*	*.024*
Full scale IQ at 8 y										
1. 80<IQ<=120	*.015*	*.002*	*.016*	*.011*	*.018*	*.005*	*-.013*	*.019*	*-.002*	*-.015*
2. IQ>120	*-.012*	*-.014*	*-.020*	*.006*	*-.020*	*.012*	*.009*	*-.012*	*.032*	*.014*
Sleep problems at 42 m										
1 (6 sleep difficulties or more)	*-.027*	*-.001*	*-.015*	*-.039*	*-.003*	*-.039*	*-.001*	*-.026*	*-.049*	*-.010*
Child's general health at 81 m										

1. minor problems	*.012*	*-.007*	*-.007*	*.003*	*.017*	*.046*	*.021*	*.017*	*.028*	*.038*
2. ill/unwell	*-.025*	*-.009*	*-.020*	*-.010*	*-.037*	*-.011*	*-.005*	*-.001*	*.007*	*-.010*
Child goes to toilet without reminder at 7.5 y										
1. never	*-.002*	*.008*	*.002*	*-.006*	*.000*	*-.041*	*-.016*	*-.011*	*-.021*	*-.018*
Child needs to dash to toilet to urinate at 7.5 y										
1. straight away	*.014*	*.021*	*.042*	*.001*	*.007*	*-.052*	*-.028*	*-.008*	*-.056*	*-.042*
Daytime wetting at 7.5 y										
1.yes	*.043*	*.031*	*.055*	*.022*	*.027*	*.038*	*.029*	*.033*	*.008*	*.010*
Social class - based on mother's occupation										
1. managerial and technical	*.003*	*-.004*	*.005*	*.003*	*-.021*	*.007*	*-.012*	*-.005*	*-.017*	*-.032*
2. Skilled non-manual	*-.021*	*-.017*	*-.025*	*-.033*	*-.010*	*-.039*	*-.038*	*-.027*	*-.039*	*-.017*
3. Skilled manual	*.005*	*.014*	*.030*	*-.029*	*.002*	*.012*	*.019*	*.045*	*-.019*	*.013*
4. Partly skilled	*.021*	*.026*	*-.001*	*.030*	*.020*	*.013*	*.040*	*.012*	*.052*	*.052*
5. Unskilled	*-.001*	*-.014*	*.023*	*.041*	*.024*	*.024*	*.008*	*.007*	*.050*	*-.004*
Social class - based on father/partner's occupation										
1. Managerial and technical	*-.003*	*-.014*	*-.011*	*-.013*	*-.014*	*.007*	*-.022*	*.010*	*-.003*	*-.020*
2. Skilled non-manual	*-.001*	*-.001*	*-.001*	*-.008*	*-.004*	*.027*	*.021*	*.014*	*.022*	*.027*
3. Skilled manual	*-.013*	*-.012*	*-.015*	*-.003*	*-.006*	*-.066*	*-.042*	*-.042*	*-.050*	*-.028*
4. Partly skilled	*.000*	*.021*	*.024*	*.011*	*.013*	*.020*	*.036*	*.013*	*.024*	*.011*
5. Unskilled	*.026*	*-.006*	*.015*	*.014*	*.011*	*.033*	*.004*	*.029*	*.016*	*.016*
Early parenthood										
1.Having child at age <19	*-.025*	*.004*	*.008*	*-.010*	*.016*	*.000*	*.022*	*.029*	*.011*	*.039*
Amount of help mother has looking after child at 85m										
1. Too little	*-.010*	*.001*	*.021*	*.009*	*.015*	*.005*	*.014*	*.037*	*.026*	*.031*
Mother's bedwetting history										
1. Mother had bedwetting after 5 y	*-.009*	*-.014*	*.008*	*-.012*	*-.030*	*-.020*	*-.030*	*-.030*	*-.033*	*-.081*
Mother daytime wetting history										

1. Mother had daytime wetting after 5 y	*.000*	*.008*	*.025*	*.002*	*.025*	*.010*	*.014*	*.029*	*.015*	*.031*
Toilet training child at 15 m										
1. Just started	*-.011*	*.001*	*.007*	*.011*	*.012*	*-.018*	*-.020*	*-.029*	*-.011*	*-.017*
2. For some time	*-.006*	*-.002*	*.005*	*-.007*	*.004*	*.023*	*.015*	*.023*	*.012*	*.026*
Rules about smoking in household										
1. Smoking allowed in some rooms	*.012*	*-.006*	*.002*	*.008*	*.009*	*.021*	*.011*	*.024*	*.026*	*.032*
2. Smoking allowed	*.027*	*.024*	*.033*	*.033*	*.002*	*.018*	*.014*	*.021*	*.006*	*-.008*
Father figure is natural father of child										
1. No / not living with father figure	*.029*	*.015*	*.037*	*.046*	*.049*	*.033*	*.024*	*.038*	*.045*	*.028*
**Related parental strategies variables (with tetrachoric correlations > .45):**										
Showing displeasure										
1. Yes						*-.031*	*-.012*	*-.027*	*-.029*	*-.043*

Table S4(i). Diagnostics for **DAYTIME TOILET TRIPS**

**N in unweighted dataset: treated = 119; untreated 1,139**	**Model adjusted for child and family variables**					**Model adjusted for child and family variables and other parental strategies**				
	**m=1**	**m=4**	**m=5**	**m=37**	**m=50**	**m=1**	**m=4**	**m=5**	**m=37**	**m=50**
**N of treated in weighted dataset:**	**636**	**630**	**645**	**637**	**639**	**651**	**659**	**659**	**649**	**657**
**N of untreated in weighted dataset:**	**622**	**628**	**613**	**621**	**619**	**607**	**599**	**599**	**609**	**601**

Figure S3. Overlap for models for **DAYTIME TOILET TRIPS** including child and family variables

m=1	m=4	m=5	m=37	m=50
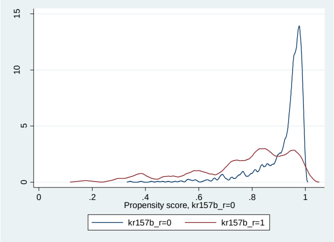	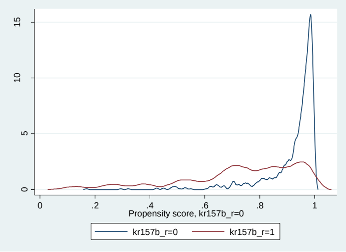	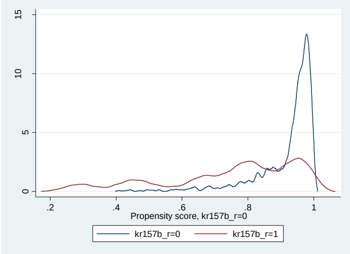	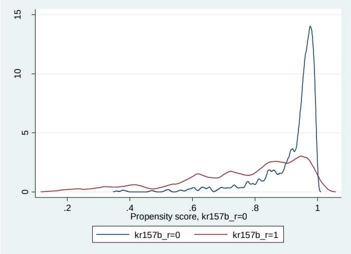	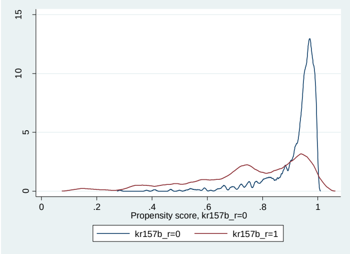
Overlap for models for **DAYTIME TOILET TRIPS** including child and family variables & other related parental strategies				
m=1	m=4	m=5	m=37	m=50
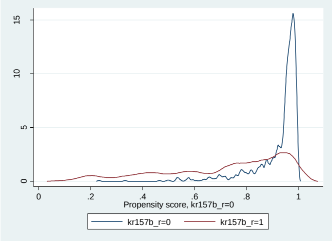	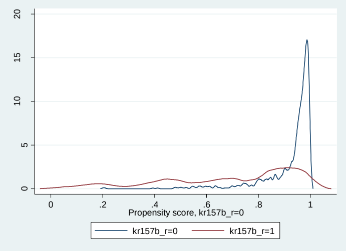	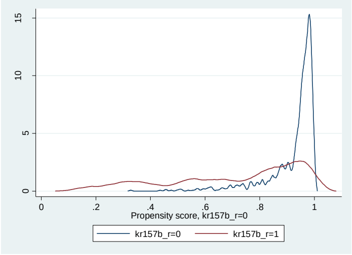	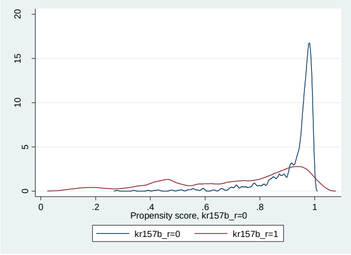	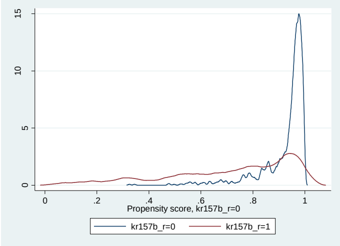

Table S4(ii). Standardized differences on confounders after weighting

**CONFOUNDERS**	**Standardized differences in weighted sample in model adjusted for child and family variables**					**Standardized differences in weighted sample in model adjusted for child and family variables and other parental strategies**				
**Imputed dataset:**	**m=1**	**m=4**	**m=5**	**m=37**	**m=50**	**m=1**	**m=4**	**m=5**	**m=37**	**m=50**
**Child and family variables included as confounders:**										
Gender										
1. Female	*-.038*	*-.042*	*-.063*	*-.025*	*-.042*	*-.032*	*-.009*	*-.038*	*-.011*	*-.037*
Temperament - Activity at 24 m										
1. High score >28	*.018*	*.049*	*.015*	*-.004*	*.020*	*.022*	*.044*	*-.002*	*.001*	*.030*
Temperament - Adaptability at 24 m										
1. High score >17	*-.015*	*.024*	*-.012*	*-.047*	*-.022*	*-.008*	*.015*	*-.019*	*-.060*	*-.021*
Temperament - Mood at 24 m										
1. High score >24	*-.021*	*-.020*	*.004*	*-.028*	*-.023*	*-.063*	*-.080*	*-.025*	*-.070*	*-.050*
Hyperactivity score at 81 m										
1. High score>6	*.025*	*.066*	*.025*	*.012*	*.024*	*.018*	*.033*	*-.009*	*-.006*	*.017*
Emotional symptoms score at 81 m										
1. High score >=4	*-.002*	*.006*	*.007*	*-.020*	*.026*	*.004*	*.015*	*.000*	*-.007*	*.015*
Conduct problems score at 81 m										
1. High score >=4	*.052*	*-.003*	*.037*	*.030*	*.040*	*.059*	*.026*	*.062*	*.044*	*.054*
Total behavioral problems at 81 m										
1. High score >=14	*.034*	*.004*	*-.007*	*-.002*	*.010*	*.039*	*.011*	*-.030*	*-.004*	*.036*
EAS temperament - activity at 69 m										
1. High score >=24	*.051*	*.098*	*.047*	*.057*	*.078*	*.028*	*.064*	*.031*	*.043*	*.056*
EAS temperament -sociability at 69 m										
1. High score >=22	*.022*	*.038*	*-.013*	*.009*	*.035*	*.001*	*.042*	*-.016*	*.011*	*.015*
Verbal IQ at 8 y										
1. 80<IQ<=120	*-.004*	*-.026*	*-.008*	*-.012*	*.029*	*-.013*	*.011*	*.016*	*.031*	*.006*
2. IQ>120	*-.017*	*-.006*	*-.022*	*.015*	*-.004*	*-.041*	*-.060*	*-.049*	*-.032*	*-.023*

Performance IQ at 8 y										
1. 80<IQ<=120	*-.018*	*-.004*	*-.037*	*-.018*	*-.008*	*-.010*	*-.001*	*-.034*	*-.032*	*-.038*
2. IQ>120	*.009*	*-.003*	*-.007*	*.008*	*.031*	*.003*	*.002*	*-.009*	*.000*	*.011*
Full scale IQ at 8 y										
1. 80<IQ<=120	*-.007*	*.044*	*.021*	*-.008*	*.034*	*-.035*	*.037*	*.046*	*.006*	*-.007*
2. IQ>120	*-.010*	*-.026*	*-.028*	*-.002*	*.012*	*-.019*	*-.047*	*-.035*	*-.018*	*.003*
Social cognition										
1. High score>=8	*.006*	*.041*	*.052*	*.006*	*-.008*	*-.011*	*.028*	*.039*	*-.003*	*.023*
Stressful life events (child related) at 42 m										
1. 5 or more stress events	*.012*	*-.069*	*.034*	*.008*	*-.017*	*.016*	*-.069*	*.017*	*-.013*	*-.002*
Sleep problems at 42 m										
1. 6 sleep problems or more	*.042*	*.081*	*.078*	*.041*	*.047*	*.067*	*.119*	*.111*	*.082*	*.065*
Child's general health at 81 m										
1. Minor problems	*.019*	*-.030*	*-.023*	*.035*	*-.014*	*.023*	*-.040*	*-.038*	*.010*	*-.020*
2. Ill/unwell	*-.100*	*-.099*	*-.067*	*-.111*	*-.073*	*-.159*	*-.163*	*-.105*	*-.147*	*-.095*
Bedtime wetting severity at 7.5 y										
1. Severe	*.016*	*.065*	*.042*	*.051*	*.021*	*.058*	*.098*	*.089*	*.095*	*.076*
Daytime wetting at 7.5 y										
1. Yes	*.011*	*.003*	*.026*	*.011*	*.023*	*.065*	*.064*	*.081*	*.056*	*.098*
Child needs to dash to toilet to urinate at 7.5 y										
1. Yes, straight away	*-.007*	*-.001*	*-.005*	*.009*	*-.010*	*.014*	*.020*	*.002*	*.020*	*-.006*
Daytime soiling at 7.5 y										
1. Yes	*.015*	*.054*	*.042*	*.006*	*.060*	*.012*	*.034*	*.030*	*-.005*	*.066*
Night time soiling at 7.5 y										
1. Yes	*.039*	*.107*	*.040*	*.024*	*.063*	*.062*	*.131*	*.056*	*.069*	*.121*
Car access										
1. No car at household when child is 33 m	*.028*	*-.033*	*.083*	*.010*	*.062*	*.010*	*-.039*	*.063*	*.008*	*.053*
Affordability index for elementary goods (mother) at 85m										

1. Severe deprivation	*.006*	*.033*	*.013*	*.016*	*-.029*	*-.001*	*.021*	*.020*	*.019*	*-.029*
Social class - based on mother's occupation										
1. Managerial and technical	*.001*	*.006*	*.022*	*-.007*	*-.042*	*-.050*	*.027*	*.045*	*.002*	*-.020*
2. Skilled non-manual	*.001*	*-.027*	*-.003*	*.006*	*.008*	*.045*	*-.032*	*-.009*	*-.008*	*.009*
3. Skilled manual	*-.014*	*.000*	*.000*	*-.048*	*.033*	*-.038*	*.000*	*.000*	*-.086*	*-.002*
4. Partly skilled	*-.008*	*.014*	*.017*	*.018*	*.012*	*.020*	*.021*	*.004*	*.034*	*-.003*
5. Unskilled	*.032*	*.028*	*-.046*	*.020*	*.029*	*.024*	*.027*	*-.027*	*.051*	*.029*
Social class - based on father's occupation										
1. Managerial and technical	*-.020*	*-.079*	*-.013*	*-.015*	*-.039*	*-.048*	*-.106*	*-.028*	*-.027*	*-.034*
2. Skilled non-manual	*.008*	*.051*	*.030*	*.032*	*.043*	*.033*	*.043*	*.031*	*.066*	*.042*
3. Skilled manual	*-.001*	*.014*	*-.017*	*-.013*	*.003*	*-.019*	*-.028*	*-.024*	*-.051*	*-.029*
4. Partly skilled	*-.026*	*.007*	*.015*	*-.014*	*-.004*	*-.012*	*.097*	*.043*	*.010*	*.031*
5. Unskilled	*.032*	*.014*	*-.006*	*.014*	*-.008*	*.054*	*.007*	*-.007*	*.026*	*-.010*
Early parenthood										
1. Had child at age <19	*-.043*	*-.026*	*-.026*	*-.029*	*-.013*	*-.079*	*-.067*	*-.067*	*-.068*	*-.045*
Amount of help mother has in looking after child at 85 m										
1. Too little	*-.008*	*-.015*	*-.012*	*-.008*	*-.013*	*-.051*	*-.053*	*-.034*	*-.032*	*-.042*
Mother's general health at 73 m										
1. Mostly well & healthy	*.004*	*.090*	*.060*	*.029*	*.032*	*-.026*	*.033*	*.048*	*.003*	*.024*
2. Often unwell & hardly ever well	*-.025*	*-.002*	*-.059*	*-.027*	*-.081*	*-.004*	*.023*	*-.069*	*-.018*	*-.053*
Mother bedwetting history										
1. mother had a problem when older than 5yo	*.014*	*.043*	*.026*	*-.004*	*.016*	*-.021*	*-.010*	*.015*	*-.048*	*-.015*
Mother day time wetting history										
1. Mother had bedwetting after 5 y	*-.033*	*-.032*	*.019*	*-.011*	*.002*	*-.071*	*-.085*	*.024*	*-.039*	*-.013*
Toilet training when child 15m										
1. Just started	*.031*	*.021*	*-.014*	*.005*	*.018*	*.017*	*.011*	*.000*	*.009*	*.011*
2. For some time	*-.038*	*-.010*	*-.041*	*.001*	*-.093*	*-.064*	*-.018*	*-.038*	*-.004*	*-.088*
Rules about smoking in household										

1. Smoking allowed in some rooms	*-.031*	*.028*	*-.001*	*-.002*	*-.006*	*-.034*	*.023*	*.028*	*.011*	*.000*
2. Smoking allowed	*-.030*	*.021*	*.014*	*-.011*	*.003*	*-.051*	*.000*	*.003*	*-.021*	*-.005*
Father figure is natural father of child										
1. No / not living with father figure	*.044*	*.023*	*.022*	*.023*	*.015*	*-.004*	*.008*	*.026*	*.029*	*.006*
**Related parental strategies variables (those with correlations > .45):**										
Restricting drinks										
1. Yes						*.104*	*.152*	*.133*	*.101*	*.146*

Table S5(i). Diagnostics for **REWARDING**

**N in unweighted dataset: treated = 169; untreated 1,089**	**Model adjusted for child and family variables**					**Model adjusted for child and family variables and other parental strategies**				
	**m=1**	**m=4**	**m=5**	**m=37**	**m=50**	**m=1**	**m=4**	**m=5**	**m=37**	**m=50**
**N of treated in weighted dataset:**	**635**	**629**	**627**	**629**	**631**	**615**	**619**	**612**	**606**	**610**
**N of untreated in weighted dataset:**	**623**	**629**	**631**	**629**	**627**	**643**	**639**	**646**	**652**	**647**

Figure S4. Overlap for models for **REWARDING** including child and family variables

m=1	m=4	m=5	m=37	m=50
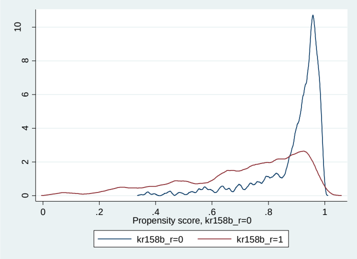	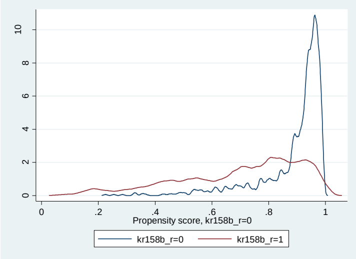	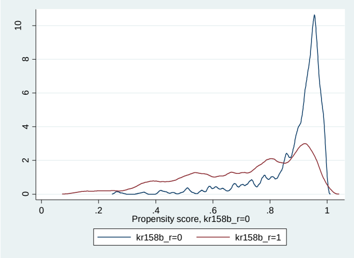	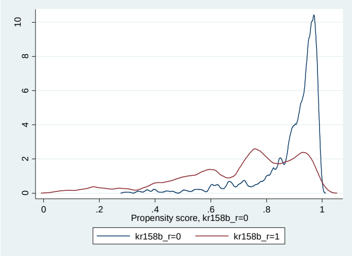	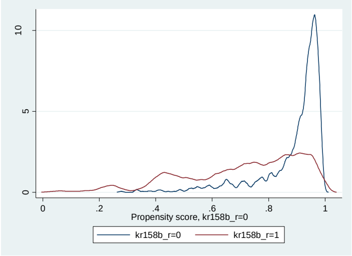
Overlap for models for **REWARDING** including child and family variables & other related parental strategies				
m=1	m=4	m=5	m=37	m=50
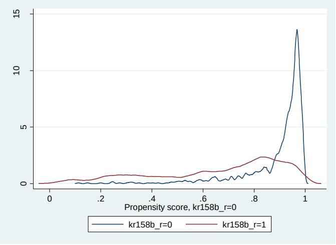	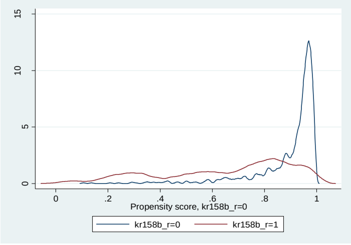	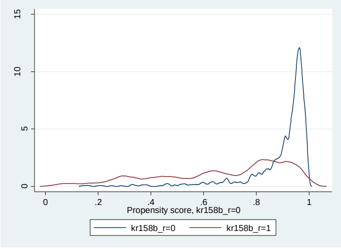	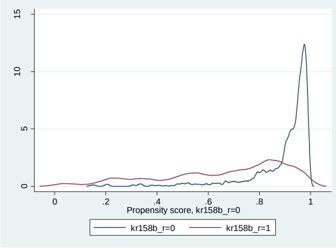	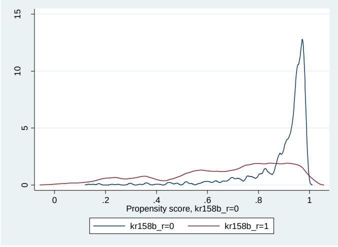

Table S5(ii). Standardized differences on confounders after weighting

CONFOUNDERS	Standardized differences in weighted sample in model adjusted for child and family variables					Standardized differences in weighted sample in model adjusted for child and family variables and other parental strategies				
**Imputed dataset:**	**m=1**	**m=4**	**m=5**	**m=37**	**m=50**	**m=1**	**m=4**	**m=5**	**m=37**	**m=50**
**Child and family variables included as confounders:**										
Gender										
1. Female	*-.071*	*-.070*	*-.070*	*-.038*	*-.066*	*-.043*	*-.006*	*-.020*	*-.011*	*.004*
Temperament - Activity at 24 m										
1. High score >28	*.019*	*-.008*	*-.008*	*-.019*	*-.004*	*.032*	*.014*	*.006*	*-.011*	*.010*
Temperament - Mood at 24 m										
1. high score >24	*.040*	*.046*	*.046*	*.033*	*.050*	*.096*	*.030*	*.048*	*.067*	*.033*
Hyperactivity score at 81 m										
1. High score>6	*.034*	*-.023*	*-.023*	*.009*	*.019*	*.125*	*.030*	*.013*	*.036*	*.033*
Emotional symptoms score at 81 m										
1. High score >=4	*.006*	*-.062*	*-.061*	*-.061*	*-.026*	*-.040*	*.032*	*-.070*	*-.001*	*-.098*
Conduct problems score at 81 m										
1. High score >=4	*-.038*	*.005*	*.005*	*-.040*	*.005*	*-.008*	*.068*	*.098*	*.028*	*.082*
Total behavioral problems at 81 m										
1. High score >=14	*.014*	*-.053*	*-.053*	*-.044*	*.011*	*.054*	*.061*	*.014*	*.055*	*.081*
EAS temperament - activity at 69 m										
1 abnormal (score >=24 (hyper active))	*.067*	*.050*	*.050*	*.077*	*.073*	*.125*	*.106*	*.095*	*.125*	*.091*
EAS temperament -sociability at 69 m										
1. High score >=22	*-.007*	*-.019*	*-.019*	*.013*	*.022*	*.097*	*.049*	*.037*	*.078*	*.036*
Verbal IQ at 8 y										
1. 80<IQ<=120	*.049*	*-.010*	*-.010*	*-.026*	*.010*	*.018*	*.138*	*.045*	*-.029*	*.027*
2. IQ>120	*-.028*	*.030*	*.030*	*.028*	*-.009*	*-.046*	*-.123*	*-.049*	*.012*	*-.061*
Performance IQ at 8 y										

1. 80<IQ<=120	*.014*	*-.023*	*-.023*	*.042*	*-.047*	*.007*	*.066*	*-.026*	*.036*	*-.082*
2. IQ>120	*-.016*	*.004*	*.004*	*-.007*	*.009*	*-.142*	*-.018*	*-.068*	*-.075*	*-.055*
Full scale IQ at 8 y										
1. 80<IQ<=120	*.032*	*.004*	*.004*	*.040*	*.003*	*-.001*	*.023*	*.074*	*.076*	*.017*
2. IQ>120	*.001*	*.032*	*.032*	*-.002*	*.017*	*-.055*	*.008*	*-.088*	*-.044*	*-.069*
Social cognition										
1. Score>=8	*.041*	*-.016*	*-.016*	*-.005*	*.007*	*.049*	*.057*	*.028*	*.070*	*.036*
Stressful life events (child related) at 42 m										
1. 5 or more stress events	*-.013*	*-.005*	*-.005*	*.012*	*-.021*	*.031*	*-.035*	*-.013*	*.006*	*-.019*
Bedwetting severity										
1. Severe	*-.018*	*-.018*	*-.018*	*-.010*	*.004*	*-.053*	*-.065*	*-.071*	*-.093*	*-.060*
Daytime wetting										
1. Yes	*-.001*	*-.019*	*-.019*	*.008*	*-.010*	*.003*	*.081*	*.057*	*.057*	*.017*
Child goes to toilet without reminder at 7.5 y										
1. Never	*.000*	*.002*	*.002*	*-.003*	*.000*	*-.012*	*-.008*	*-.024*	*-.002*	*-.005*
Night time soiling at 7.5 y										
1. Yes	*.057*	*.039*	*.039*	*.005*	*-.007*	*.068*	*.082*	*.084*	*.081*	*-.006*
Affordability index for elementary goods (mother) at 85m										
1. Severe deprivation	*.032*	*.006*	*.006*	*-.025*	*.012*	*.099*	*.089*	*.068*	*.010*	*.076*
Social class - based on mother's occupation										
1. Managerial and technical	*.047*	*.008*	*.008*	*-.004*	*-.019*	*.002*	*.116*	*.085*	*.033*	*-.003*
2. Skilled non-manual	*-.057*	*-.023*	*-.023*	*-.010*	*-.026*	*-.078*	*-.169*	*-.143*	*-.053*	*-.077*
3. Skilled manual	*.071*	*-.018*	*-.018*	*-.025*	*.049*	*.042*	*.069*	*.012*	*-.063*	*-.001*
4. Partly skilled	*.002*	*.018*	*.018*	*.010*	*.003*	*.024*	*.021*	*.039*	*.034*	*.049*
5. Unskilled	*-.032*	*.003*	*.003*	*.043*	*.059*	*.073*	*.045*	*.036*	*.059*	*.086*
Social class - based on father’s occupation										
1. Managerial and technical	*-.035*	*-.033*	*-.033*	*-.051*	*-.013*	*-.071*	*-.055*	*-.097*	*-.054*	*-.009*

2. Skilled non-manual	*.054*	*.038*	*.038*	*.035*	*.061*	*.083*	*.057*	*.087*	*.113*	*.096*
3. Skilled manual	*.030*	*.011*	*.011*	*.029*	*-.019*	*-.028*	*.045*	*.013*	*-.039*	*-.089*
4. Partly skilled	*-.028*	*-.020*	*-.020*	*.000*	*.007*	*.005*	*-.044*	*-.008*	*-.021*	*.026*
5. Unskilled	*-.006*	*.018*	*.018*	*.010*	*.000*	*.067*	*.007*	*.045*	*.050*	*.021*
Early parenthood										
1. Had child at age <19	*.011*	*.024*	*.024*	*.027*	*.044*	*.039*	*.035*	*.047*	*.057*	*.067*
Amount of help mother has in looking after child at 85m										
1. Too little	*.038*	*.069*	*.069*	*-.002*	*.022*	*.106*	*.125*	*.155*	*.131*	*.134*
Mother thinks she spends enough time with child (85m)										
1. No	*.010*	*.012*	*.012*	*.025*	*.032*	*.009*	*.027*	*-.027*	*.032*	*.011*
Mother's state of health 73m										
1. Mostly well & healthy	*.062*	*.023*	*.023*	*.040*	*.040*	*.090*	*-.034*	*.024*	*.033*	*.063*
2. Often unwell & hardly ever well	*-.048*	*-.056*	*-.056*	*-.032*	*-.027*	*.034*	*.022*	*-.067*	*.041*	*.049*
Mother bedwetting history										
1. Mother had bedwetting after 5 y	*.011*	*-.002*	*-.002*	*.037*	*.031*	*.058*	*-.034*	*.011*	*.050*	*.044*
Mother's daytime wetting history										
1. Mother had daytime wetting after 5 y	*.002*	*-.056*	*-.056*	*-.009*	*.013*	*.052*	*.059*	*.009*	*.055*	*.073*
Toilet training when child 15m										
1. Just started	*-.024*	*-.028*	*-.028*	*.009*	*.009*	*.065*	*-.048*	*.011*	*.023*	*.010*
2. For some time	*.005*	*.000*	*.000*	*.010*	*.024*	*.006*	*.023*	*.026*	*.042*	*.047*
**Related parental strategies variables (those with correlations > .45):**										
Restricting drinks										
1. Yes						*-.081*	*-.067*	*-.051*	*-.099*	*-.097*
Alarm prevention										
1. Yes						*-.219*	*-.098*	*-.202*	*-.168*	*-.187*

Table S6(i). Diagnostics for **SHOWING DISPLEASURE**

**N in unweighted dataset: treated = 55; untreated 1,203**	**Model adjusted for child and family variables**					**Model adjusted for child and family variables and other parental strategies**				
	**m=1**	**m=4**	**m=5**	**m=37**	**m=50**	**m=1**	**m=4**	**m=5**	**m=37**	**m=50**
**N of treated in weighted dataset:**	**625**	**627**	**631**	**623**	**626**	**629**	**625**	**628**	**619**	**622**
**N of untreated in weighted dataset:**	**633**	**631**	**628**	**635**	**631**	**629**	**633**	**630**	**639**	**636**

Figure S5. Overlap for models for **SHOWING DISPLEASURE** including child and family variables

m=1

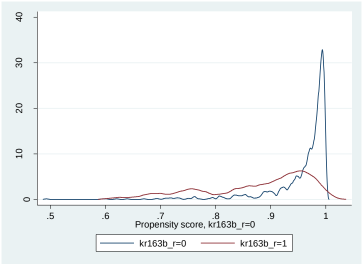

m=4

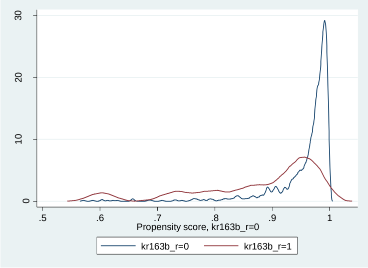

m=5

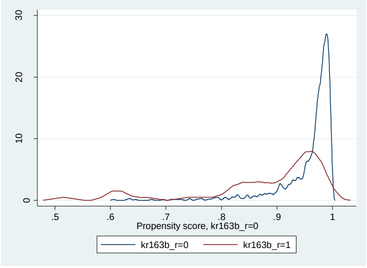

m=37

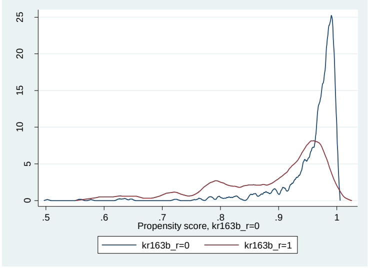

m=50

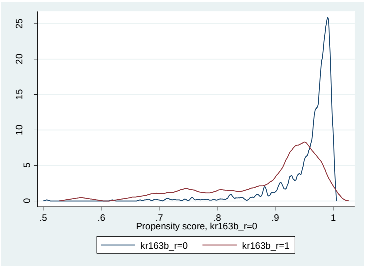

Overlap for models for **SHOWING DISPLEASURE** including child and family variables & other related parental strategies

m=1

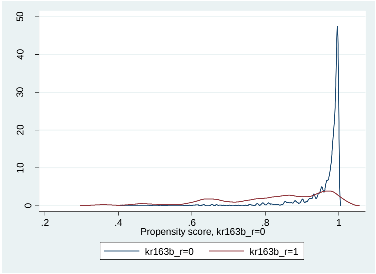

m=4

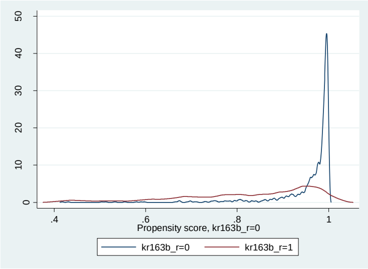

m=5

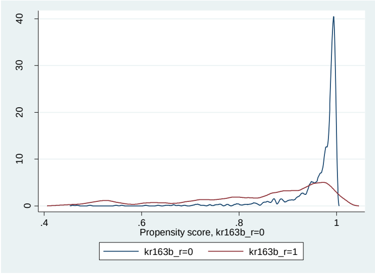

m=37

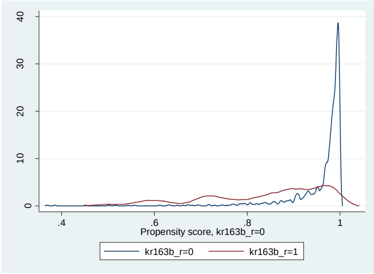

m=50

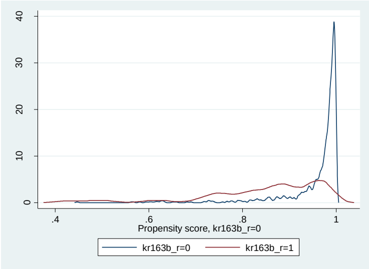

Table S6(ii). Standardized differences on confounders after weighting

**CONFOUNDERS**	**Standardized differences in weighted sample in model adjusted for child and family variables**					**Standardized differences in weighted sample in model adjusted for child and family variables and other parental strategies**				
**Imputed dataset:**	**m=1**	**m=4**	**m=5**	**m=37**	**m=50**	**m=1**	**m=4**	**m=5**	**m=37**	**m=50**
**Child and family variables included as confounders:**										
Gender										
1. Female	*.013*	*.043*	*.030*	*.020*	*.034*	*-.004*	*.049*	*.031*	*-.006*	*.020*
Temperament - Mood at 24 m										
1. High score >24	*-.029*	*-.010*	*-.013*	*.000*	*-.009*	*.009*	*.015*	*.001*	*.026*	*.008*
Hyperactivity score at 81 m										
1. High score>6	*.003*	*.006*	*.009*	*-.010*	*.002*	*-.027*	*-.029*	*-.012*	*-.049*	*.007*
Emotional symptoms score at 81 m										
1. High score >=4	*-.010*	*.000*	*-.014*	*.010*	*-.003*	*-.037*	*-.029*	*-.050*	*-.001*	*-.012*
Conduct problems score at 81 m										
1. High score >=4	*.033*	*.044*	*.037*	*.013*	*.030*	*.009*	*.046*	*.044*	*-.011*	*.035*
Total behavioral problems at 81 m										
1. High score >=14	*.008*	*.011*	*.015*	*.003*	*-.008*	*-.025*	*-.023*	*.006*	*-.028*	*-.010*
EAS temperament - activity at 69 m										
1. High score >=24	*.014*	*-.007*	*.002*	*.016*	*-.007*	*.021*	*-.023*	*-.021*	*.011*	*-.010*
EAS temperament -sociability at 69m										
1. High score >=22	*.037*	*.048*	*.045*	*.060*	*.060*	*.062*	*.076*	*.063*	*.095*	*.098*
Verbal IQ ay 8 y										
1. 80<IQ<=120	*-.001*	*.013*	*.000*	*-.012*	*-.007*	*-.011*	*-.016*	*-.015*	*-.044*	*-.014*
2. IQ>120	*-.011*	*-.014*	*-.012*	*.008*	*.003*	*-.005*	*.010*	*.004*	*.034*	*.009*
Performance IQ at 8 y										
1. 80<IQ<=120	*.020*	*-.013*	*.016*	*.005*	*-.007*	*.025*	*-.048*	*.008*	*-.007*	*-.002*
2. IQ>120	*-.035*	*-.031*	*-.032*	*-.015*	*-.017*	*-.028*	*-.017*	*-.022*	*.012*	*-.017*
Full scale IQ at 8 y										

1. 80<IQ<=120	*.018*	*.016*	*.018*	*.006*	*.004*	*.006*	*-.014*	*-.008*	*-.034*	*-.005*
2. IQ>120	*-.021*	*-.029*	*-.019*	*-.007*	*-.010*	*.002*	*.004*	*.003*	*.044*	*-.001*
Social cognition										
1.(score>=8)	*-.001*	*.013*	*-.010*	*.018*	*-.007*	*.004*	*.024*	*.000*	*.031*	*.006*
Stressful life events (child related) at 42 m										
1. 5 or more stress events	*-.034*	*-.008*	*-.009*	*-.019*	*-.025*	*.006*	*.011*	*-.022*	*-.057*	*-.001*
Sleep problems as 42 m										
1. 6 sleep problems or more	*-.005*	*.001*	*-.033*	*-.025*	*.000*	*-.040*	*-.018*	*-.062*	*-.083*	*-.030*
Child's general health at 81 m										
1. Minor problems	*.007*	*-.006*	*.017*	*.014*	*.009*	*.001*	*.005*	*.024*	*.043*	*-.012*
2. Ill/unwell	*-.058*	*-.042*	*-.032*	*-.060*	*-.057*	*-.040*	*-.049*	*-.006*	*-.064*	*-.011*
Child goes to toilet without reminder at 7.5 y										
1. Never	*-.033*	*-.011*	*-.027*	*-.016*	*.002*	*-.072*	*-.038*	*-.020*	*-.032*	*-.007*
Bedwetting severity at 7.5 y										
1. Severe	*.001*	*-.038*	*-.013*	*-.003*	*-.015*	*.005*	*-.058*	*-.016*	*-.002*	*-.029*
Daytime wetting at 7.5 y										
1. Yes	*-.025*	*-.018*	*.011*	*-.018*	*-.014*	*-.036*	*-.011*	*-.019*	*-.052*	*-.035*
Family size										
1. 3 or more children	*.003*	*-.002*	*-.001*	*-.028*	*-.026*	*.068*	*.014*	*.037*	*.034*	*-.009*
Affordability index for elementary goods (mother) at 85m										
1. Severe deprivation	*.002*	*.008*	*.011*	*.014*	*.016*	*.031*	*.051*	*.021*	*.041*	*.008*
Social class - based on mother's occupation										
1. Managerial and technical	*-.017*	*-.033*	*-.038*	*-.021*	*-.016*	*-.012*	*-.052*	*-.035*	*-.057*	*.005*
2. Skilled non-manual	*.014*	*.022*	*.011*	*.000*	*-.006*	*-.003*	*-.013*	*.002*	*-.005*	*-.024*
3. Skilled manual	*.031*	*.029*	*.037*	*.014*	*.018*	*.025*	*.028*	*.039*	*.005*	*.011*
4. Partly skilled	*-.001*	*.021*	*-.007*	*.019*	*.005*	*-.009*	*.032*	*-.005*	*.074*	*.008*
5. Unskilled	*.016*	*-.014*	*.048*	*.003*	*.033*	*.007*	*-.004*	*.064*	*.009*	*.035*
Social class - based on father's										

occupation										
1. Managerial and technical	-.001	-.012	-.019	.007	.001	.025	.019	.017	.024	.015
2. Skilled non-manual	.014	-.016	-.006	-.001	-.015	.028	.015	.007	.027	.004
3. Skilled manual	-.025	.008	.007	.006	-.008	-.073	-.044	-.036	-.042	-.031
4. Partly skilled	.018	.027	.052	-.004	.030	.042	.026	.053	.007	.028
5. Unskilled	.027	.004	.000	.000	.000	.023	.017	.005	.000	.000
Early parenthood										
1. Had child at age <19	.000	-.023	.008	-.011	.000	.047	-.041	.024	.028	.035
Amount of help mother has in looking after child at 85 m										
1. Too little	-.024	-.006	-.007	-.035	-.021	-.033	-.012	.030	-.048	.006
Mother's general health 73 m										
1. Mostly well & healthy	-.020	-.009	-.023	-.011	-.015	-.044	-.050	-.043	-.010	-.012
2. Often unwell & hardly ever well	.010	.029	.009	.023	-.006	.034	.063	.025	.055	.024
Mother's daytime wetting history										
1. Mother had daytime wetting after 5 y	.004	.018	.005	-.018	.029	.010	.016	-.001	-.005	.046
Toilet training when child 15m										
1. Just started	.034	.037	.034	.026	.029	.013	.042	.026	.014	.025
2 .For some time	.000	-.016	.007	-.004	.017	.061	.015	.040	.066	.057
Rules about smoking in household										
1. Smoking allowed in some rooms	.021	.016	.002	.011	.020	.014	-.014	-.006	-.010	.023
2. Smoking allowed	-.003	-.007	-.020	-.031	-.052	-.029	.002	-.044	-.043	-.052
Father figure is natural father of child										
1. No / not living with father figure	.020	.017	-.001	.008	.027	.035	-.004	-.041	.024	-.007
**Related parental strategies variables (those with correlations > .45):**										
Restricting drinks										
1.yes						.023	.003	.008	-.014	-.007

Table S7(i). Diagnostics for **PROTECTION PANTS**

**N in unweighted dataset: treated = 98; untreated =1,160**	**Model adjusted for child and family variables**				
	**m=1**	**m=4**	**m=5**	**m=37**	**m=50**
**N of treated in weighted dataset:**	**637**	**638**	**629**	**621**	**626**
**N of untreated in weighted dataset:**	**621**	**620**	**629**	**637**	**632**

Figure S6. Overlap for models for **PROTECTION PANTS** including child and family variables

m=1

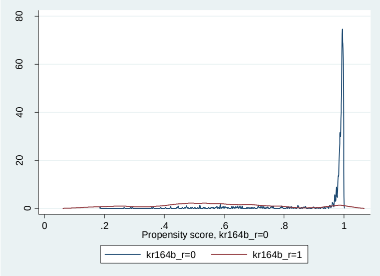

m=4

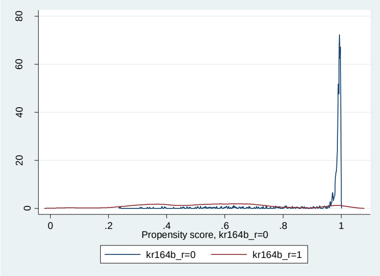

m=5

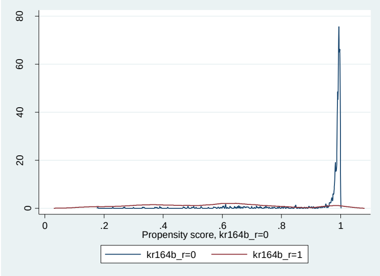

m=37

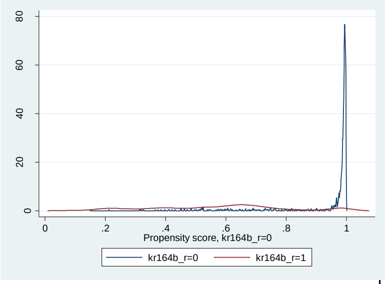

m=50

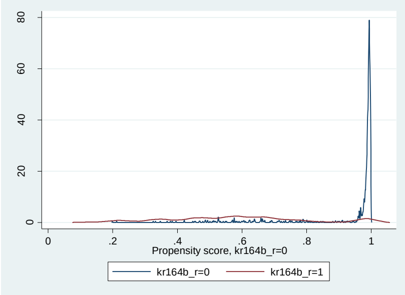

No other strategy was correlated with Protection pants strategy above the agreed level. Therefore in this case the above table includes only one row of figures.

Table S7(ii). Standardized differences on confounders after weighting

**CONFOUNDERS**	**Standardized differences in weighted sample in model adjusted for child and family variables**				
**Imputed dataset:**	**m=1**	**m=4**	**m=5**	**m=37**	**m=50**
**Child and family variables included as confounders:**					
Gender					
1. Female	*.042*	*.028*	*.087*	*.041*	*.086*
Temperament - Adaptability at 24 m					
1. High score >17	*.000*	*.036*	*.053*	*.010*	*.021*
Temperament - Mood at 24 m					
1. High score >24	*-.030*	*-.048*	*-.013*	*.007*	*-.011*
Conduct problems score at 81 m					
1. High score >=4	*-.014*	*.010*	*-.022*	*.037*	*.058*
Total behavioral problems at 81 m					
1. High score >=14	*-.067*	*-.005*	*-.085*	*.048*	*.015*
EAS temperament - activity at 69 m					
1. High score >=24	*.015*	*-.018*	*-.006*	*.039*	*-.013*
EAS temperament -sociability at 69 m					
1. High score >= 22	*.038*	*.058*	*.052*	*-.043*	*.014*
Social cognition					
1. Score>=8	*-.025*	*-.008*	*-.013*	*-.014*	*-.009*
Full scale IQ at 8 y					
1. 80<IQ<=120	*-.016*	*-.059*	*.005*	*.072*	*.022*
2. IQ>120	*.032*	*.116*	*.037*	*.006*	*.078*
Verbal IQ at 8 y					
1. 80<IQ<=120	*-.090*	*-.141*	*-.074*	*-.065*	*-.086*
2. IQ>120	*.058*	*.130*	*.057*	*.053*	*.109*
Performance IQ at 8 y					
1. 80<IQ<=120	*-.139*	*-.176*	*-.130*	*.016*	*-.002*
2. IQ>120	*.072*	*.201*	*.142*	*.037*	*.086*

Stressful life events (child related) at 42 m					
1. 5 or more stressful events	*.041*	*-.008*	*.030*	*-.039*	*-.034*
Sleep problems as 42 m					
1. 6 sleep problems or more	*.001*	*.000*	*.019*	*.026*	*-.008*
Child's general health at 81m					
1. Minor problems	*.029*	*.012*	*.006*	*.093*	*.056*
2. Ill/unwell	*-.007*	*-.057*	*.040*	*-.010*	*.011*
Bedwetting severity at 7.5 y					
1. Severe	*.010*	*.012*	*.000*	*-.012*	*-.004*
Daytime wetting at 7.5 y					
1. Yes	*.024*	*.022*	*.041*	*-.022*	*-.022*
Daytime soiling					
1. Yes	*-.058*	*-.087*	*-.043*	*-.030*	*-.007*
Child needs to dash to toilet to urinate at 7.5 y					
1. Yes, straight away	*-.005*	*-.031*	*.002*	*.019*	*-.020*
Affordability index for elementary goods (mother) at 85m					
1. Severe deprivation	*.055*	*.071*	*.088*	*.048*	*.074*
Rules about smoking in household					
1. Smoking allowed in some rooms	*.039*	*.007*	*-.044*	*.054*	*-.002*
2. Smoking allowed	*.072*	*.060*	*.053*	*.055*	*.048*
Father figure is natural father of child					
1. no / not living with father figure	*-.008*	*-.015*	*-.014*	*-.005*	*.001*
**Related parental strategies variables (those with correlations > .45):**					
None					
