# Supplementary material for: Examining the effectiveness of parental strategies to overcome bedwetting: an observational cohort study
Source: BMJ Open. 2017 Jul 13;7(7):e016749. doi: 10.1136/bmjopen-2017-016749 (PMC5541498; doi:10.1136/bmjopen-2017-016749)
Supplement: Author's manuscript [file bmjopen-2017-016749.draft_revisions.pdf]

BMJ Open

Examining the effectiveness of parental strategies to prevent bedwetting: an observational cohort study

Journal:	*BMJ Open*
Manuscript ID	bmjopen-2017-016749
Article Type:	Research
Date Submitted by the Author:	07-Mar-2017
Complete List of Authors:	Grzeda, Mariusz; University of Bristol School of Social and Community Medicine<br>Heron, Jon; University of Bristol, Centre for Academic Mental Health, ; University of Bristol. UK<br>Tilling, Kate; University of Bristol, Social Medicine<br>Wright, Anne; Evelina London Children's Hospital<br>Joinson, Carol; University of Bristol, Centre for Child and Adolescent Health; School of Social and Community Medicine, University of Bristol
**Primary Subject Heading**:	Paediatrics
Secondary Subject Heading:	Epidemiology
Keywords:	Bedwetting, prevention, child, cohort study, ALSPAC

SCHOLARONE™
Manuscripts

For peer review only

For peer review only - http://bmjopen.bmj.com/site/about/guidelines.xhtml

Examining the effectiveness of parental strategies to prevent bedwetting: an observational cohort study

Corresponding author: Carol Joinson, University of Bristol, Oakfield House, Oakfield Grove, Clifton, Bristol, BS8 2BN. Email address: Carol.Joinson@bristol.ac.uk, Phone: 0117 331 3360

Authors: Mariusz T Grzeda MSci[1], Jon Heron PhD[1], Kate Tilling PhD[1], Anne Wright MBBCh[2], Carol Joinson PhD[1]

Affiliations:

1. School of Social and Community Medicine, University of Bristol, Oakfield House, Oakfield Grove, Clifton, Bristol BS8 2BN, UK.
2. Evelina London Children's Hospital, St Thomas' Hospital, Westminster Bridge Road, London SE1 7EH.

Word count: 3686.

For peer review only

ABSTRACT

Objective: To examine whether a range of common strategies used by parents to prevent bedwetting in 7½-year-old children (including lifting, restricting drinks before bedtime, regular daytime toilet trips, rewards, showing displeasure and using protection pants) are effective in reducing the risk of bedwetting at 9½ years.

Design: Prospective cohort study.

Setting: General community.

Participants: The starting sample included 1,258 children (66.7% boys and 33.2% girls) who were still bedwetting at 7½ years.

Outcome measure: Risk of bedwetting at 9½ years.

Results: Using propensity score based methods we found that two of the parental strategies used at 7½ years were associated with an increased risk of bedwetting at 9½ years, after adjusting the model for child and family variables and other parental strategies: lifting (risk difference= 0.11 [95% CI= 0.01 to 0.20]) and restricting drinks before bedtime (0.12 [0.02 to 0.23]). The effect of using the other parental strategies was in either direction (an increase or decrease in the risk of bedwetting at 9½ years) e.g. showing displeasure (-0.05 [-0.21 to 0.11). When we re-analysed the data using multivariable regression analysis, the results were mostly consistent with the propensity score-based methods.

Conclusion: These findings provide evidence that common strategies used to prevent bedwetting in 7½ year olds are not effective in reducing the risk of bedwetting at 9½ years. Parents should be encouraged to seek professional advice for their child's bedwetting rather than persisting with strategies that may be ineffective.

Keywords: Bedwetting, prevention, child, cohort study, ALSPAC.

For peer review only

Strengths and limitations of this study:

- Major strengths of this study are the availability of prospective data on bedwetting and a wide range of covariates associated with both bedwetting and the parental strategies in a large birth cohort.
- Use of propensity score-based methods with observational data makes it easier to assess whether observed confounding has been adequately eliminated.
- We did not separately examine whether the parental strategies are differentially effective for children with non-monosymptomatic and monosymptomatic nocturnal enuresis.
- We did not have information on the onset or duration of the strategies to prevent bedwetting and, therefore, we were only able to assess strategies that were reported by parents as being used currently.
- There were very small numbers of parents using medication or bedwetting alarms so we were unable to examine the effectiveness of these interventions in this study.

For peer review only

INTRODUCTION

Attainment of bladder control is a major milestone in child development that marks the end of a period of toilet training that is sometimes prolonged and stressful for parents and children. Nocturnal enuresis is the term used by the International Children's Continence Society (ICCS) to describe bedwetting in children aged 5 years or older after ruling out organic causes[1]. It is most common for children to become dry during the day before they remain dry at night, [2] with children usually attaining night-time bladder control between the ages of four and six years[3]. Although a significant proportion of children continue to wet the bed at school age, only a small proportion wet the bed at least twice a week, the frequency required for DSM-V diagnosis of nocturnal enuresis. For example, bedwetting was reported in 15.5% of 7½ year olds in the Avon Longitudinal Study of Parents and Children (ALSPAC), but only 2.6% wet the bed twice a week or more at this age[4]. With increasing age, bedwetting becomes more socially unacceptable and is often met with intolerance and frustration from parents[5]. Bedwetting also places considerable practical and financial burdens on the family in terms of the extra workload of washing bed linen and the cost of protective pants[6]. The psychosocial implications of bedwetting among school age children include worries about participating in sleepovers and school trips, fear of detection, teasing from peers, a sense of being different from others, emotional distress and low self-esteem[6]. Achieving bladder control is, therefore, important for a child's health and wellbeing.

It is a common belief among parents that bedwetting will eventually resolve with age and, as a result, many parents delay seeking treatment for bedwetting until it is having is a considerable impact on the child and family[7]. There is evidence from randomised and quasi-randomised trials that treatment of bedwetting with an alarm or medication can be effective[8,9], but many parents are unaware that effective treatments for bedwetting are

For peer review only - http://bmjopen.bmj.com/site/about/guidelines.xhtml

available[10,11]. Before seeking medical advice, parents often employ a range of simple strategies aimed at preventing bedwetting, the most common being restricting drinks before bed, lifting (removing the sleeping child from bed to empty the bladder in the toilet or potty), rewarding for being dry, regular daytime toilet trips, using protection pants and showing displeasure[10]. Caldwell et al.[12] conducted a systematic review of randomised (or quasi-randomised) trials of simple behavioural interventions that are often used by parents as first line interventions for bedwetting. The studies compared the interventions with an appropriate comparison group, comprising no active treatment, other behavioural interventions, or drugs either alone or in combination with other interventions. They found evidence that simple behavioural interventions (e.g. rewards, lifting) were more effective in promoting dryness than no intervention, but were inferior to treatments such as alarms and medication. The authors of the review noted, however, that most trials were small and of poor methodological quality and caution should be exercised in interpreting the findings.

Although the best method for drawing causal inferences about interventions is a randomized controlled trial (RCT), this type of study design is not always feasible or ethical. When an RCT is not possible, observational studies are the only available type of evidence. Causality is difficult to assess in such studies because of the possibility of confounding – where one or more variable(s) influence both the exposure and the outcome, and thus it appears that there is a causal link between them. One method to assess the effectiveness of an intervention whilst adjusting for known and measured confounders is by using methods based on propensity scores. The aim of this study is to apply propensity score-based methods to observational data from a birth cohort to examine the effectiveness of a range of parental strategies aimed at preventing childhood bedwetting and to compare this with results using regression methods.

METHODS

Participants

The sample comprised participants from the Avon Longitudinal Study of Parents and Children. Detailed information about ALSPAC is available on the study website (http://www.bristol.ac.uk/alspac), which includes a fully searchable dictionary of available data (http://www.bris.ac.uk/alspac/researchers/data-access/data-dictionary). Pregnant women resident in the former Avon Health Authority in south-west England, having an estimated date of delivery between 1/4/91 and 31/12/92 were invited to take part, resulting in a cohort of 14,541 pregnancies and 13,973 singletons/twins (7,217 boys and 6,756 girls) alive at 12 months[13]. Ethical approval for the study was obtained from the ALSPAC Law and Ethics committee and local research ethics committees. Written informed consent was obtained after the procedure(s) had been fully explained.

Bedwetting at 7½ and 9½ years: The starting sample for this study comprised children who were still bedwetting at 7½ years. Parents were asked "How often does your child wet him/herself during the night?" and for both questions were given the response options 'Never'; 'Occasional accident but less than once a week'; 'About once a week'; '2-5 times a week'; 'Nearly every day; and 'More than once a day'. A total of 8,151 parents responded to this questionnaire and 1,258 children (15.4%) were still wetting the bed at 7½ years, comprising 840 (66.7%) boys and 418 (33.2%) girls. Of the 1,258 children, 215 (69.8% boys and 30.2% girls) wet the bed 'at least twice a week'. At age 9½ years the questions on frequency of bedwetting were repeated. Bedwetting data were provided for 8,101 children and 788 (9.7%) wet the bed at this age (120 of these children wet the bed at least twice a week). The proportion of children with bedwetting at 7½ years whose parents provided data at age 9½ was 83% (1,045 out of 1,258) and 47% (n= 493) were still wetting the bed at this

For peer review only - http://bmjopen.bmj.com/site/about/guidelines.xhtml

age, comprising 357 (72.4%) boys and 136 (27.6%) girls. Table 1 describes the characteristics of the study participants.

For peer review only

Table 1. Characteristics of the study participants at 7½ and 9½ years.

	7½ years (n=1,258)	9½ years (n=1,045)
Gender		
Female	33.2%	32.8%
Male	66.8%	67.2%
n	1,258	1,045
Social class[1]		
Non-manual (professional, managerial, skilled professions)	82.0%	82.3%
Manual (partly or unskilled occupations)	18.0%	17.7%
n	1,094	923
Home ownership[1]		
Owner/occupier	85.5%	87.5%
Rented accommodation	14.5%	12.5%
n	1,112	952
Car access[1]		
Yes	91.1%	91.8%
No	8.9%	8.2%
n	1,132	965
Maternal education[1]		
A-level or above	43.9%	46.9%

For peer review only

O-level	33.6%	33.4%
Certificate of secondary school / vocational/ none	22.5%	19.7%
n	1,226	1,021

1. These variables were derived from responses to a questionnaire completed by mothers during the antenatal period.

For peer review only

Parental strategies to prevent bedwetting at 7½ years

A questionnaire was administered to parents when the study children were aged 7½ that contained a list of strategies aimed at preventing bedwetting (see table 2) that were originally elicited from parents[10]. Parents were asked: "*Which of the following methods have you tried in the past or are you using now to try and help your child stop wetting the bed*?" We restricted our analysis to strategies that parents reported '*using now*' because there was no information on the onset or duration of strategies used in the past. The questionnaire explained that these strategies are not necessarily effective in preventing bedwetting.

For peer review only - http://bmjopen.bmj.com/site/about/guidelines.xhtml

Table 2. Associations between the parental strategies used to prevent bedwetting in children at 7½ years (n=1,258).

	Lifting child out of bed to use toilet	Restricting drinks before bedtime	Regular daytime toilet trips	Rewarding child for being dry	Showing displeasure when child wets bed	Using nighttime protection pants / nappies	Medication for bedwetting	Bedwetting alarm
Lifting	1.000							
Restricting drinks	0.509	1.000						
Daytime toilet trips	0.295	0.413	1.000					
Rewards	0.444	0.407	0.523	1.000				
Showing displeasure	0.236	0.456	0.323	0.345	1.000			
Protection pants	-0.038	-0.151	0.231	0.249	0.040	1.000		
Medication	0.238	0.164	0.416	0.423	0.016	0.296	1.000	
Bedwetting alarm	0.050	-0.099	0.230	0.492	-0.070	0.230	0.113	1.000

Note: The associations between strategies in terms of tetrachoric correlation coefficients. All coefficients were computed on the baseline sample.

For peer review only - http://bmjopen.bmj.com/site/about/guidelines.xhtml

Rationale for using propensity score methods in this study

Baseline characteristics of children whose parents used particular strategies to prevent bedwetting (referred to as 'treated' hereafter) might be expected to differ systematically from those whose parents did not use the strategy ('non-treated'), giving rise to confounding. It is, therefore, necessary to account for these differences when estimating the effect of the parental strategy ('treatment') on bedwetting. This could be achieved through multivariable regression (adjusting for differences in baseline variables between treated and untreated participants). Propensity score matching (PSM) has been proposed as a more appropriate method to minimise confounding when estimating treatment effects using observational data[14]. A detailed description of our rationale for using propensity score-based methods in our study is provided in the online supplementary materials.

Statistical analysis

We used PSM based methods to assess the effectiveness of the parental strategies to prevent bedwetting and compared this with estimates derived using logistic regression. Both of these analyses aimed to estimate the effect of each parental strategy (used by parents of children who wet the bed at 7 ½ years) on risk of bedwetting at 9½ years.

Propensity score-based model: We assessed the effectiveness of each strategy by examining the difference in risk of bedwetting at 9½ years between children receiving the parental strategy ('treated') versus those children not receiving the strategy ('non-treated'). The estimate we computed was the *average treatment effect for the treated* (ATT)[15] (see supplementary material for an explanation of our choice of ATT as the measure of treatment effect). In order to put the results of PSM analyses in context we report estimates obtained both from unadjusted models (without any confounders) and from models adjusted for the set of measured confounders.

For peer review only - http://bmjopen.bmj.com/site/about/guidelines.xhtml

We implemented the inverse probability weighting (IPW) method using the propensity score because there is evidence that this method is relatively less biased than other methods based on propensity scores[15,16]. Aside from this, in the reweighting based on propensity scores, as opposed to matching on propensity scores, no case is excluded from the analysis. The baseline variables we selected into the propensity score model were those that were associated with the treatments (parental strategies used at 7 ½ years) and outcome (bedwetting at 9 ½ years).

We used a two-stage procedure to derive the list of baseline variables to include in the model. Firstly, we examined two logistic regression models: in model 1 the outcome variable was predicted from all theoretically relevant variables relating to children and their environment (see table S1 in the supplementary materials for the list of all variables included) In model 2, the parental strategy was the outcome variable. In the second stage, we applied a threshold for selection of variables to the propensity score model. The variables we included were those that had associations (expressed in terms of odds ratios) with the outcome variables in both models of <0.90 or >1.20, thus allowing us to detect at least 'weak' associations[17]. We estimated models with and without adjustment for the confounders. A detailed list of the confounding variables is provided in table S1. Parents frequently used multiple strategies concurrently (indicated by strong correlations between subsets of parental strategies – table 2). For this reason, at the second stage of analysis, we repeated the IPW models for every strategy adjusting for confounders and strategies that were correlated (correlation >0.45, see table 2) with the particular strategy used as the outcome variable in the model. Before analysing the treatment effects, we assessed the adequacy of the propensity score model using diagnostic procedures detailed in the online supplementary material (see tables and figures S2 – S7).

Logistic regression model: We also examined the effectiveness of the parental

For peer review only - http://bmjopen.bmj.com/site/about/guidelines.xhtml

strategies to prevent bedwetting by using multivariable logistic regression to adjust for confounders. We applied logistic regression models to the same set of baseline variables used in the propensity score model and reported the results in the form of adjusted risk differences (rather than odds ratios) for ease of comparison with the results from the propensity score-based model. We estimated risk differences through the STATA *logit* command followed by *adjrr* command[18]. The latter command returned estimates equivalent to average treatment effects (see supplementary materials). In order to obtain robust estimates of the coefficients and their standard errors we used loops developed in STATA in exactly the same way as for propensity score model.

Missing data

Many of the potential confounders we considered had some missing data, thus reducing the sample size available for analyses. The number of missing cases on confounding variables ranged from 0 (gender) to 659 (mother's social class based on occupation) (see table S1). There were no missing data on exposure (treatment) variables. There were 213 missing cases on the outcome variable (bedwetting at 9½ years).

The exact numbers describing the amount of missing data per variable is presented in the supplementary materials (table S1). To deal with the missing data, we used multiple imputation by chained equations[19] within ICE (Imputation by Chained Equations) STATA package v.14[20]. Full details of the methods used are provided in the supplementary materials.

For peer review only

RESULTS

Figure 1 shows the prevalence of each strategy used by parents of 7½ year old children and the total number of strategies employed by parents. The most common strategies for preventing bedwetting were restricting drinks, rewarding and lifting. Only a very small number of parents used medication or bedwetting alarms, so we were unable to examine the effectiveness of these interventions. Over 50% of parents did not use any of the strategies to prevent bedwetting, whilst over 26% of parents used only one strategy. Table 2 shows the associations between the strategies. The strongest correlations were found between lifting and restricting drinks and between regular daytime toilet trips and rewards.

Table 3 shows the average treatment effects on treated for each parental strategy used at 7½ years on risk of bedwetting at 9½ years derived from the propensity score based analysis. The table also shows the average treatment effects derived from the logistic regression model. The coefficients in table 3 are estimated differences between the risk of bedwetting at 9½ years after receiving given ‘treatment’ (parental strategy) and the risk of bedwetting if they had remained ‘untreated’. Figure 2 displays the results of both analyses as forest plots.

For peer review only

For peer review only - http://bmjopen.bmj.com/site/about/guidelines.xhtml

Table 3. Estimated differences in the risk of bedwetting at 9½ years in children whose parents used each strategy compared with those who did not use the strategy.

	Average treatment effect on treated (95% CI) based on inverse probability weighting using propensity score			Average treatment effect / adjusted risk difference (95% CI) based on logistic regression analysis		
	Empty (unadjusted) model[a]	Model adjusted for child and family variables[b]	Model adjusted for child and family variables and other parental strategies[c]	Empty (unadjusted) model[d]	Model adjusted for child and family variables[e]	Model adjusted for child and family variables and other parental strategies[f]
Lifting	0.251 (0.167-0.335)	0.148 (0.059-0.237)	0.106 (0.009-0.202)	0.251 (0.167-0.335)	0.172 (0.081-0.263)	0.125 (0.029-0.221)
Restricting drinks	0.173 (0.110-0.236)	0.138 (0.071-0.205)	0.123 (0.021-0.226)	0.173 (0.110-0.236)	0.147 (0.083-0.211)	0.112 (0.045-0.180)
Daytime toilet trips	0.198 (0.100-0.296)	0.088 (-0.024-0.201)	0.068 (-0.047-0.182)	0.198 (0.100-0.296)	0.087 (-0.024-0.198)	0.057 (-0.056-0.171)
Rewards	0.281 (0.201-0.361)	0.141 (0.049-0.233)	0.088 (-0.014-0.191)	0.281 (0.201-0.361)	0.158 (0.062-0.254)	0.143 (0.044-0.242)
Showing displeasure	0.054 (-0.091-0.199)	0.009 (-0.146-0.164)	-0.052 (-0.214-0.11)	0.054 (-0.091-0.199)	0.009 (-0.134-0.151)	-0.047 (-0.187-0.093)
Protection pants	0.290 (0.182-0.398)	-0.010 (-0.161-0.141)	-0.010 (-0.161-0.141)	0.290 (0.182-0.398)	0.025 (-0.134-0.184)	0.025 (-0.134-0.184)

The estimates provided in this table are average treatment effects for each strategy. They are estimated differences between the risk of bedwetting at 9 ½ years after receiving given 'treatment' (i.e. parental strategy) and the risk of bedwetting if they had remained 'untreated'.

For peer review only - http://bmjopen.bmj.com/site/about/guidelines.xhtml

[a] *Empty model* column for propensity score based methods analysis shows unadjusted differences in risk of bedwetting between ‘treated’ and ‘untreated’ children. These coefficients are equivalent to bivariate regressions including bedwetting at 9 ½ years as an outcome variable and each strategy as a single predictor.

[b] *Model adjusted for child and family variables* represents average treatment effects on treated children (ATETs). These coefficients are estimated differences in risks of bedwetting in weighted samples. These are differences in risks adjusted for child and family variables that accounted for the differences between treated and untreated groups and between those with and without bedwetting at age 9 ½. The list of child and family variables was derived separately for every strategy on the basis of odds ratios (ORs) from regression analyses providing evidence of an association (ORs <0.90 or >1.20) both with the given strategy and with bedwetting at age 9 ½. A detailed list of child and family variables included in model for each parental strategy is provided in the online supplementary material (see tables S2 – S7).

[c] *Model adjusted for child and family variables and other parental strategies.* This is the column with coefficients adjusted for the child and family variables and for other parental strategies that were highly correlated (coefficient of tetrachoric correlation: >.45) with given strategy.

[d] *Empty model*: results of univariable logistic regression analyses including bedwetting at 9 ½ years as an outcome variable and the given strategy as a single predictor. To ensure direct comparisons with the output of the analysis using propensity score based methods, the results of regression analyses are expressed here in terms of risk differences instead of ORs.

[e] *Model adjusted for child and family variables.* This includes estimated differences in risk of bedwetting for given strategy obtained from logistic regression, also including child and family variables.

[f] *Model adjusted for child and family variables and other parental strategies.* Risk differences for given strategy obtained from logistic regression including child and family variables and other strategies associated with the strategy under examination.

For peer review only - http://bmjopen.bmj.com/site/about/guidelines.xhtml

Propensity score based results (inverse probability weighting)

The unadjusted propensity score based analysis shows that the parental strategies used at 7½ years are associated with an increase in the risk of bedwetting at 9½ years. Showing displeasure was the only strategy that was also associated with a small decrease in the risk of bedwetting in the unadjusted model. After applying the propensity score model adjusting for the child and family variables and then further adjusting for the other strategies, the adjusted treatment effects provided evidence that lifting and restricting drinks are associated with an increase in the risk of bedwetting at 9 ½ years. The adjusted results for the other strategies indicated that the effect could be in either direction (either an increase or decrease in the risk of bedwetting).

Logistic regression results

The results obtained from the logistic regression analysis were mostly consistent with the analysis using the propensity score-based methods i.e. both lifting and restricting drinks were associated with an increase in the risk of bedwetting at 9½ years in the fully adjusted model. Using rewards was also associated with an increased risk of bedwetting at 9½ years.

For peer review only

DISCUSSION

We examined a range of common strategies used by parents to prevent bedwetting and found that when these strategies were used with 7½ year-old children who wet the bed, they were not effective in reducing the risk of bedwetting at 9½ years. Parental strategies including lifting and restricting drinks before bedtime were associated with an increased risk of subsequent bedwetting. These were among the most common parental strategies used to prevent bedwetting in our study and there is evidence in a review that these strategies are frequently used by parents across different countries[10].

This is the first study, to our knowledge, to apply propensity score-based methods to examine the effectiveness of parental strategies to prevent bedwetting using observational data from a large birth cohort. Austin [14] discusses several reasons for preferring the use of propensity score based methods to regression models when estimating treatment effects using observational data. In particular, it is easier to assess whether observed confounding has been adequately eliminated using propensity score based methods. It is important to note some caveats associated with PSM-based methods and these are discussed in the supplementary materials. Another major strength of this study is the availability of a wide range of confounders in the ALSPAC dataset that are associated with bedwetting and with the parental strategies to prevent bedwetting. We did not separately examine whether the parental strategies are differentially effective for children with non-monosymptomatic (bedwetting with daytime wetting and/or lower urinary tract symptoms [21]) and monosymptomatic nocturnal enuresis (bedwetting without these symptoms)[22]. There is evidence that frequent childhood bedwetting that is accompanied by daytime wetting is less likely to resolve with age[23,24]. If we had compared children with monosymptomatic and non-monosymptomatic nocturnal enuresis this would necessarily have reduced of the number of cases included in the analysis, resulting in serious problems with estimation of the

For peer review only - http://bmjopen.bmj.com/site/about/guidelines.xhtml

propensity score-based models. We did, however, include a range of covariates in the analyses including frequency of bedwetting (high frequency= twice or more/week), daytime wetting, urgency and voiding postponement. All of these variables were important confounders in most of the models. We did not have information on the onset or duration of the strategies to prevent bedwetting and, therefore, we were only able to assess strategies that were reported by parents as being used currently. Due to this being a community-based, rather than a clinical, sample, there were very small numbers of parents using medication or bedwetting alarms. We were, therefore, unable to examine the effectiveness of these interventions in this study. Additionally, there was no information on which strategies parents initiated compared with those recommended by health care workers or other sources (e.g. the child's grandparents).

Caldwell *et al*.[12] concluded from their systematic review, that simple interventions such as lifting (or waking) could initially be tried as a strategies to prevent bedwetting, since such methods are "safe and are better than doing nothing". However, they cautioned that most of the trials they reviewed were small and of poor methodological quality[12]. Our findings are consistent with the current advice given in the NICE guidelines stating that lifting will not promote long-term dryness[25]. Although lifting appears to be sensible for promoting nighttime dryness, its effectiveness as an intervention for reducing or stopping bedwetting has previously been questioned. Lifting may inadvertently maintain bedwetting by delaying the acquisition of dryness, because this strategy encourages the child to empty the bladder without fully waking. This is counterproductive since children are not learning to waken to the sensation of a full bladder, and therefore, bedwetting persists[10,26]. Lifting the child from their bed to pass urine can occur with or without waking the child, but this distinction was not possible in our study since parents were not specifically asked whether they woke the child when lifting them to use the toilet or potty. It could be argued, however,

For peer review only - http://bmjopen.bmj.com/site/about/guidelines.xhtml

that lifting (either with or without waking) denies the child the opportunity to learn to wake in response to bladder signals without assistance. There is evidence that restricting drinks might increase the risk of bedwetting by reducing the ratio of bladder volume to overnight urine production[27]. Restricting drinks one hour before bedtime is often recommended especially drinks with diuretic properties[28] and drinks containing caffeine[25]. Current NICE guidelines state that adequate daily fluid intake and using the toilet at regular intervals throughout the day are important in the management of bedwetting[25]. We did not find evidence that encouraging regular daytime toilet trips resulted in a decreased risk of bedwetting at 9 ½ years, but our findings are based on parental reports rather than a detailed diary of toileting and fluid intake completed during treatment. Using positive (rewards) or negative reinforcements (showing displeasure) as strategies aimed at preventing bedwetting may be problematic because rewarding outcomes that the child has no control over (i.e. a dry night) could lead children to feel that they have failed if they continue to wet the bed[29]. It is recommended to reward steps towards nighttime dryness that the child *does* have control over (e.g. going to the toilet before bed without prompting, drinking and passing urine at regular intervals throughout the day, helping to change sheets)[25]. Protection pants (nappies, pull-ups) are often recommended as useful short-term solution to supporting children during school trips and sleepovers. However, health professionals do not recommend prolonged use of protection pants because this may not help children to become dry in the long-term. Instead, parents are advised to consider using alternative bed protection such as waterproof sheets[25]. A trial of at least 2 nights in a row without protection pants is recommended for children under 5 years who are still bedwetting, but have been dry during the day for more than 6 months[25].

The majority of children experience a natural resolution of bedwetting with increasing age (spontaneous resolution rate is estimated to be 15% per year[30]). Parents and clinicians,

For peer review only - http://bmjopen.bmj.com/site/about/guidelines.xhtml

therefore, often adopt a *'wait and see'* approach to childhood bedwetting, due to the common belief that it will resolve with age[7]. There is increasing evidence, however, that children who experience frequent bedwetting and those who have bedwetting with additional daytime bladder symptoms are at risk of their problems persisting into adolescence. A cross-sectional study of 5 – 19 year olds found a greater proportion of frequent bedwetting (>= 3 wet nights per week) in older (11 – 19 years) compared with younger children (5 – 10 years)[23]. A prospective study found that children with persistent bedwetting with accompanying daytime wetting had a higher chance of becoming adolescents with bedwetting compared with those who had bedwetting alone in childhood[24]. These findings suggest that parents of children with these patterns of bedwetting should seek early advice from a health professional. In the ALSPAC cohort, however, only 31.9% of parents of 7 ½ year-old children with bedwetting sought professional help[10]. It was previously common practice not to offer treatment for bedwetting until the child was aged at least 7 years. However, the current NICE guidelines for nocturnal enuresis recommend that children over the age of 2 years, with on-going wetting problems, both day and night, who are showing appropriate toileting awareness and behaviour, should be considered for assessment and investigation to exclude a specific medical problem[25]. If initial advice and support does not lead to resolution of bedwetting, NICE recommend that first line treatment options should be offered to all children with bedwetting, particularly those whose families find the management of bedwetting burdensome and request help. Infrequent bedwetting at age five is not uncommon, but if children are still frequently wetting the bed at this age they may need professional help to become dry at night. There is evidence that treating childhood bedwetting is cost-effective compared to leaving it untreated[25]. Successful treatment can result in children having improved self-esteem[31,32] and quality of life[33].

This study adds further weight to the importance of encouraging parents to seek

For peer review only - http://bmjopen.bmj.com/site/about/guidelines.xhtml

professional help for their child's bedwetting, rather than persisting with strategies that may be ineffective. Replication of our findings in other samples would provide further reassurance of our findings. Future research is needed to determine whether there are risk factors in early childhood that predict the continuation of bedwetting at school age. Early identification of the underlying causes of a child's bedwetting, including overactive bladder, nocturnal polyuria and/or problems with sleep and arousal, could help to ensure that appropriate treatment is given.

For peer review only

For peer review only - http://bmjopen.bmj.com/site/about/guidelines.xhtml

Acknowledgments: This study is based on the Avon Longitudinal Study of Parents and Children (ALSPAC). We are extremely grateful to all the families who took part in this study, the midwives for their help in recruiting them, and the whole ALSPAC team, which includes interviewers, computer and laboratory technicians, clerical workers, research scientists, volunteers, managers, receptionists and nurses.

Collaborator: Dr Penny Dobson provided advice on the strategies used by parents to prevent bedwetting and on the current NICE guidelines.

Competing interests: We have read and understood BMJ policy on declaration of interests and declare that we have no competing interests.

Funding statement: This work was supported by the Medical Research Council grant number MR/L007231/1. The UK Medical Research Council, the Wellcome Trust (Grant reference: 102215/2/13/2) and the University of Bristol provide core support for ALSPAC.

Author contributions: Drs Joinson and Heron, Mr Grzeda, Professor Tilling and Dr Wright conceptualized and designed the study. Mr Grzeda carried out the statistical analysis and drafted the result section. Drs Heron, Joinson and Professor Tilling supervised the statistical analysis and interpreted the results. All authors were involved in drafting the manuscript and approved the final manuscript as submitted.

Data sharing statement: This is a secondary data analysis based on data from the ALSPAC cohort. The ALSPAC Executive encourages and facilitate data sharing with all 'bona fide' researchers. The access policy for the ALSPAC data can be found at http://www.bristol.ac.uk/media-library/sites/alspac/documents/ALSPAC_access_policy.pdf.

REFERENCES

1. Austin PF, Bauer SB, Bower W et al. The standardization of terminology of lower urinary tract function in children and adolescents: update report from the Standardization Committee of the International Children's Continence Society. *J Urol* 2014;191(6):1863-1865.e13.
2. Jansson UB, Hanson M, Sillén U, Hellström AL. Voiding pattern and acquisition of bladder control from birth to age 6 years--a longitudinal study. *J Urol* 2005;174(1):289-93.
3. Fergusson DM, Horwood LJ, Shannon FT. Factors related to the age of attainment of nocturnal bladder control: an 8-year longitudinal study. *Pediatrics* 1986;78(5):884-90.
4. Butler RJ, Golding J, Northstone K; ALSPAC Study Team. Nocturnal enuresis at 7.5 years old: prevalence and analysis of clinical signs. *BJU Int* 2005;96(3):404-10.
5. Butler R, McKenna S. Overcoming parental intolerance in childhood nocturnal enuresis: a survey of professional opinion. *BJU Int* 2002;89(3):295-7. Review.
6. Schulpen TW. The burden of nocturnal enuresis. *Acta Paediatr* 1997;86(9):981-4.
7. Berry AK. Helping children with nocturnal enuresis: the wait-and-see approach may not be in anyone's best interest. *Am J Nurs* 2006;106(8):56-63.
8. Glazener CM, Evans JH, Peto RE. Alarm interventions for nocturnal enuresis in children. *Cochrane Database Syst Rev* 2003;(2):CD002911. Review.
9. Glazener CM, Evans JH, Peto RE. Tricyclic and related drugs for nocturnal enuresis in children. *Cochrane Database Syst Rev* 2003;(3):CD002117. Review.
10. Butler RJ, Golding J, Heron J; ALSPAC Study Team. Nocturnal enuresis: a survey of parental coping strategies at 7 1/2 years. *Child Care Health Dev* 2005;31(6):659-67.

For peer review only - http://bmjopen.bmj.com/site/about/guidelines.xhtml

11. Schlomer B, Rodriguez E, Weiss D, Copp H. Parental beliefs about nocturnal enuresis causes, treatments, and the need to seek professional medical care. *J Pediatr Urol* 2013;9(6 Pt B):1043-8.

12. Caldwell PH, Nankivell G, Sureshkumar P. Simple behavioural interventions for nocturnal enuresis in children. *Cochrane Database Syst Rev* 2013;19(7):CD003637.

13. Boyd, A, Golding, J, Macleod, JAA, et al. Cohort Profile: The 'Children of the 90s'--the index offspring of the Avon Longitudinal Study of Parents and Children. *International Journal of Epidemiology* 2013;42:111-127.

14. Austin PC. An introduction to Propensity Score Methods for Reducing the Effects of Confounding in Observational Studies. *Multivariate Behavioral Research* 2011;46: 399-424.

15. Imbens GW. Nonparametric Estimation of Average Treatment Effects under Exogeneity: A Review. National Bureau of Economic Research. *The Review of Economics and Statistics* 2004, 86(1): 4–29.

16. Lunceford JK, Davidian M. Stratification and weighting via the propensity score in estimation of causal treatment effects: A comparative study. *Statistics in Medicine* 2004;23, 2937–2960.

17. Monson RR. Occupational Epidemiology, 2nd Edition, CRC Press, Boca Raton, London, New York, Washington 1990.

18. Norton EC, Miller MM, Kleinman LC. Computing adjusted risk ratios and risk differences in Stata. The Stata Journal, 2001.

19. Royston P, White IR. Multiple Imputation by Chained Equations (MICE): Implementation in Stata, Journal of Statistical Software 2011; 45(4), 1-20.

For peer review only - http://bmjopen.bmj.com/site/about/guidelines.xhtml

20. StataCorp. 2015. *Stata Statistical Software: Release 14*. College Station, TX: StataCorp LP.

21. Franco I, von Gontard A, DeGennaro M. Evaluation and treatment of nonmonosymptomatic nocturnal enuresis: A standardization document from the International Children's Continence Society. *Journal of Pediatric Urology* 2013;9: 234-243.

22. Nevéus T, von Gontard A, Hoebeke P, et al. The standardization of terminology of lower urinary tract function in children and adolescents: report from the Standardisation Committee of the International Children's Continence Society. *J Urol* 2006; 176(1): 314-24.

23. Yeung CK, Sreedhar B, Sihoe JD, et al. Differences in variables of nighttime enuresis between children and adolescents: a critical appraisal from a large epidemiological study. *BJU Int* 2006; 97(5): 1069-1073.

24. Heron J, Grzeda M, von Gontard A, Wright A, Joinson C. Trajectories of urinary incontinence in childhood and bladder and bowel symptoms in adolescence: prospective cohort study. *BMJ Open*, in press.

25. NICE (2010). Nocturnal enuresis: The management of bedwetting in children and young people. NICE Clinical Guidelines, No. 111. National Clinical Guideline Centre, 2010.

26. Christmanson L, Lisper H. Parent behaviors related to bed-wetting and toilet-training as etiological factors in primary enuresis. Scandinavian Journal of Behaviour Therapy. *Scandinavian Journal of Behaviour Therapy* 1982;52 11(1):29-37.

27. Sorotzkin B. Nocturnal enuresis: Current perspectives. *Clinical Psychology Review* 1984;4:293–317.

For peer review only - http://bmjopen.bmj.com/site/about/guidelines.xhtml

28. Novello AC, Novello JR. Enuresis. *Pediatric Clinics of North America* 1987;34(3):719–33.

29. Blackwell C. A guide to enuresis: A guide to treatment of enuresis for professionals. Bristol: ERIC, 1989.

30. Forsythe WI, Redmond A. Enuresis and spontaneous cure rate: study of 1129 enuretics. *Arch Dis Child* 1974*;*49, 259-263.

31. Longstaffe S, Moffatt ME, Whalen JC. Behavioral and self-concept changes after six months of enuresis treatment: a randomized, controlled trial. *Pediatrics* 2000;105(4 Pt 2):935-40.

32. Hägglöf B, Andrén O, Bergström E, Marklund L, Wendelius M. Self-esteem in children with nocturnal enuresis and urinary incontinence: improvement of self-esteem after treatment. *Eur Urol* 1998;33 Suppl 3:16-9.

33. Equit M, Hill J, Hübner A, von Gontard A. Health-related quality of life and treatment effects on children with functional incontinence, and their parents. *J Pediatr Urol* 2014;10(5):922-8.

For peer review only - http://bmjopen.bmj.com/site/about/guidelines.xhtml

Figure 1

Prevalence of parental strategies for preventing bedwetting (figure 1a) and prevalence of children with parents employing a given number of strategies (figure 1b).

Figure 2

Estimated differences in the risk of bedwetting at 9 ½ years in children whose parents used each strategy compared with those who did not use the strategy: results of the propensity score-based model (figure 2a) and the logistic regression analysis (figure 2b).

For peer review only

For peer review only - http://bmjopen.bmj.com/site/about/guidelines.xhtml

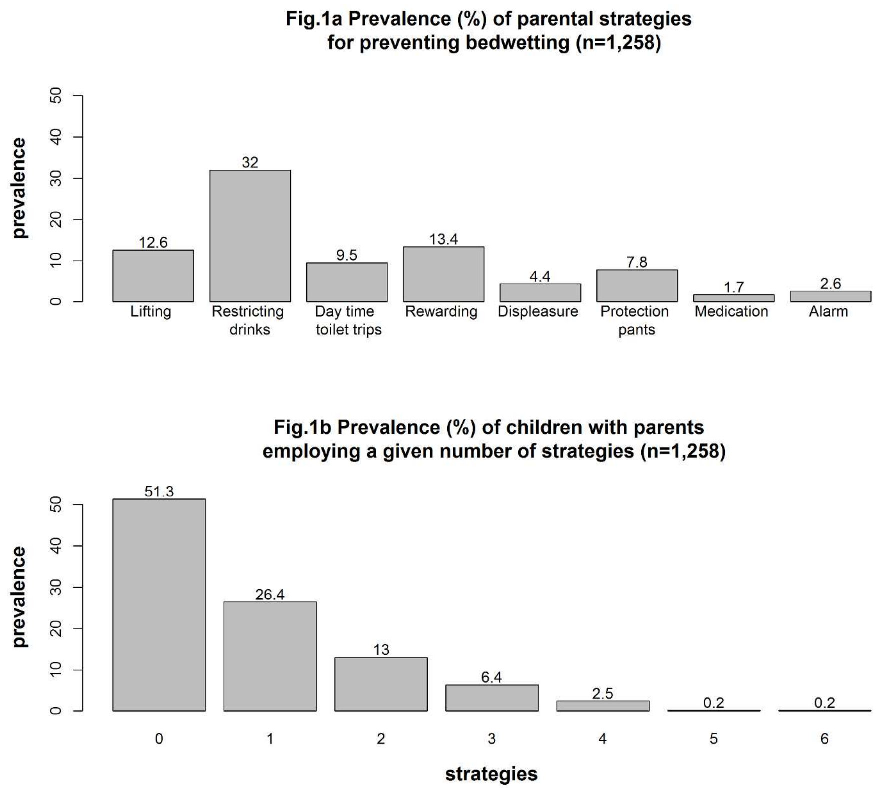

Figure 1

279x228mm (300 x 300 DPI)

For peer review only

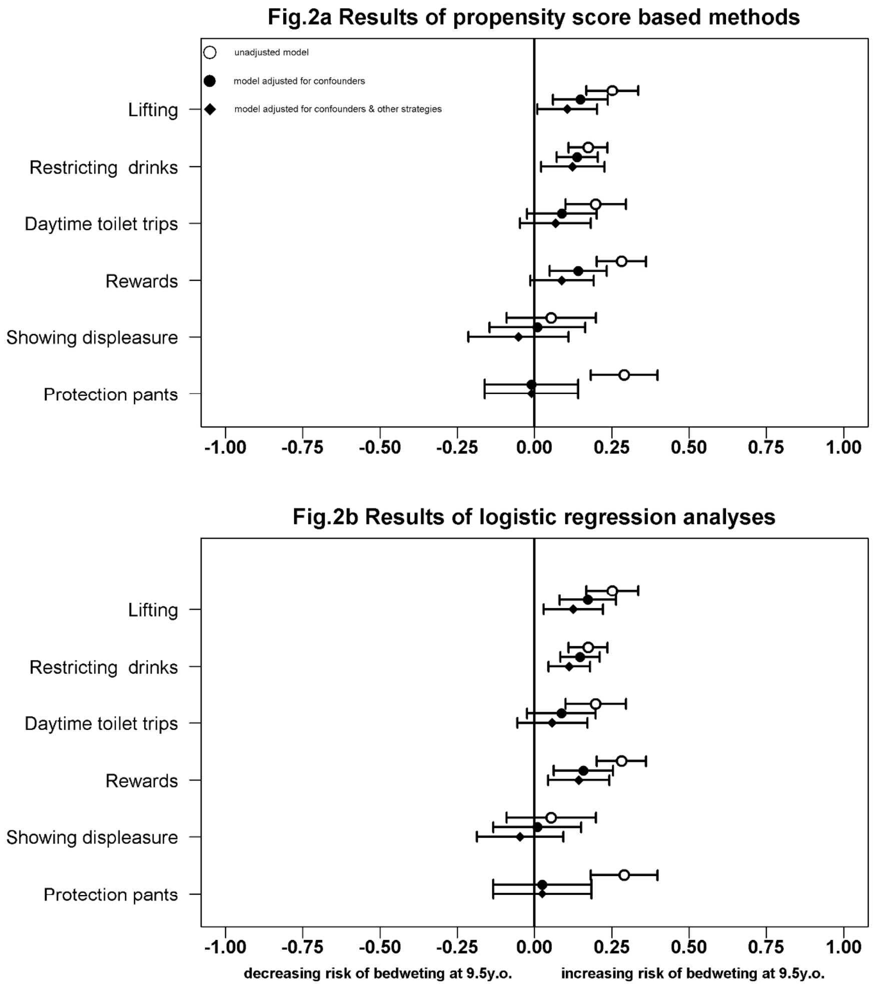

Figure 2

228x228mm (300 x 300 DPI)

Supplementary online material

Rationale for applying propensity score methods

The main purpose of propensity score-based methods is to draw causal inferences about effects of treatments from observational data. In order to do this certain assumptions are required. In observational data the process of selection to the 'treated' and 'non-treated' groups is not random. Direct comparisons of the outcomes in both groups are likely to be biased if 'treated' and 'non-treated' groups differ systematically on certain variables (both measured and unmeasured) that may also be associated with the outcome. It is, therefore, not possible to establish to what extent the observed results are effects of the 'treatment' versus other important variables that remained uncontrolled in the process of selection of cases into the groups. Dealing with this problem of confounding is a central problem of studies based on observational data. As Rosenabum (2005)[1] states the key issues in such studies are to remove 'overt biases' (related to measured/observed confounders) and to address somehow uncertainty about those hidden (unmeasured confounders).

Multivariable regression and, more recently, propensity score-based methods can be used to minimise effects of confounders when estimating treatment effects using observational data. These methods adjust the raw results to take into account the non-random mechanism of selection of cases to the 'treated' versus 'non-treated' groups by controlling for differences between both groups. Propensity score-based methods are based on the assumption that differences between 'treated' and 'non-treated' groups can be explained in terms of observable variables and bias should, therefore, be minimised by ensuring similarity of both groups on these variables. This is achieved using propensity score-based methods either by matching each case from the 'treated' group to at least one similar case from the 'non-treated' group or by re-weighting both groups in order to construct a synthetic sample in which both groups are similar with respect to the observed confounders. Similarity of the groups is assessed in terms of the probability of receiving the 'treatment' as estimated from the observed variables of the cases. This probability, called the 'propensity score', is further used to create/or reshape the sample in which distributions of variables of study participants are similar in both groups. If this condition is fulfilled then treated and untreated groups are balanced on measured confounders. However, in order to compare their results directly it is also important to assume that all of the important confounders were measured and included in the analysis. It is only under these conditions that estimates of causal effects are assumed to be unbiased.

Application of propensity score-based methods in the current study

We used propensity score-based methods to assess the effectiveness of parental strategies aimed at preventing bedwetting. Within our study, 'treated' refers to those children receiving each parental strategy at 7 ½ years and 'non-treated' refers to those not receiving the strategy.
The propensity score-based analysis involves three general steps: (1) constructing the propensity scores; (2) balancing the sample based on their propensity scores; and (3) estimating effects of the treatment (parental strategy). At each of these steps in the analysis, decisions need to be made that influence the results and final conclusions. Below, we present the key decisions we made in conducting the propensity score-based analysis:

a) Average treatment effect versus average treatment effect on treated:

For peer review only - http://bmjopen.bmj.com/site/about/guidelines.xhtml

One of the most crucial decisions in the analysis concerns the indicator of the impact of the treatment. These are: average treatment effect (ATE) or average treatment effect on treated (ATT). They are both rooted within the counterfactual framework, which assumes that each participant included in the study has two potential outcomes i.e. the outcome when treatment was applied and the outcome when treatment was not applied. In reality, only one of these outcomes is truly observed, and the other is unobserved (counterfactual). The ATE is defined as the difference between averages of these two potential outcomes at the population level. The ATT is the difference between potential outcomes calculated for the treated group.
The aim of the analysis was to estimate the effect of each parental strategy aimed at preventing bedwetting at 7½ years as a difference in risk of bedwetting at 9½ years between those children receiving the strategy (treated) versus those children not receiving the strategy (non-treated). We assessed the effectiveness of each parental strategy by means of the average treatment effect for the treated (ATT) because we were interested in the average effect of treatment on those children who ultimately received the treatment rather than in the estimation of the average treatment effect at the population level (ATE).

b) Selection of propensity score based method:
The four most reported methods for using propensity scores in the estimation of the effect of treatment are propensity score matching, stratification on propensity score, inverse probability weighting and covariate adjustment using propensity scores. Austin (2011)[2] provides a detailed discussion of the strengths and limitations of these methods. In brief, the method based on inverse probability weighting is favoured, whilst the other methods were found to be more prone to bias in Monte Carlo studies. We used the inverse probability of treatment weighting (IPTW) where the synthetic balanced sample is constructed (participant variables are independent on the assignment to treated/non-treated groups). We estimated the propensity scores by logistic regression.

c) *Selection of baseline variables to the propensity score model*:
There are at least four possible strategies for variable selection and there is lack of consensus in the literature on which strategy is the most appropriate.[2] The available strategies are: (1) propensity model should include all measured, theoretically relevant variables; (2) model should include variables that are associated with the treatment; (3) model should include variables that are associated with the outcome; (4) model should include only those variables that are associated both with treatment and outcome. The final strategy seems to be the favoured approach as regards theoretical arguments and results of simulation studies.[2] Therefore, we chose this model including only those variables that are associated both with treatment (parental strategy) and outcome (bedwetting at 9 ½ years). We used a two-stage procedure to derive the list of baseline variables to include in the model. Firstly, we conducted two logistic regression analyses in the first completed dataset (m=1). In the first regression model, the outcome variable was predicted from all theoretically relevant variables relating to children and their family (see table S1 for the list of all variables included). In the second regression model, each parental strategy was the outcome variable. Secondly, we applied a threshold for selection of variables to the propensity score model - only the variables that had associations (expressed in terms of odds ratios) with the outcome variables in both models of <0.90 or >1.20.[3] We enriched the propensity score models by including the parental strategies that were highly correlated with the

For peer review only - http://bmjopen.bmj.com/site/about/guidelines.xhtml

specific strategy considered in each model. The alternative strategy was included in the model for a given strategy if the tetrachoric correlation was >0.45.

d) *Diagnostics:*
Propensity score based diagnostic methods are designed to examine whether the distributions of the baseline variables are the same/or similar enough in treated and non-treated groups. There are several specific diagnostic procedures that are recommended. Among them the most frequently used are those examining the standardized differences between means of baseline variables after matching/weighting and those comparing their variances. Before analysing the treatment effects, we assessed the adequacy of the propensity score model by checking the overlap in propensity scores. We based our diagnostic checks on the examination of differences between means of baseline variables in the weighted sample. Categorical variables with more than two categories were examined using sets of binary variables. Following the literature we assumed that absolute standardized mean difference < 0.1 indicates negligible differences between groups. Since the model computations were conducted on 50 datasets obtained from the multiple imputation procedure (see section below for details) we performed the diagnostic checks in 5 randomly selected datasets (m=1,4,5,37,50). See tables S2 to S7 for the results of the diagnostic checks to assess the adequacy of the propensity score models.

e) Dealing with missing data and estimating the treatment effects:
The magnitude of missing data per variable is presented in table S1. To deal with the missing data, we used multiple imputation by chained equations within ICE (Imputation by Chained Equations) STATA package v.14. We generated 50 imputed datasets using 10 cycles of regression switching. We used a range of auxiliary data, which does not itself form part of the analytical model. The criteria of variable selection to the imputation model for each variable included: (i) theoretical and empirical evidence from prior published research; (ii) data driven: variable was selected if in the current dataset it was strongly associated with observed values of the imputed variable or missingness on that variable; (iii) additionally, the imputation models for every variable included the parental strategies and bedwetting at 9½ years.
Conducting the analysis based on propensity scores on multiply imputed datasets required a decision about the exact routine by which the treatment effects are calculated. There are two possibilities: (1) first estimate the propensity score in each of 50 completed datasets and average the propensity score for every subject across all datasets and then estimate treatment effects based on those aggregated propensity scores; (2) estimate propensity scores and treatment effects separately in every completed dataset and then aggregate the treatment effects obtained according to Rubin's rules. Published simulation studies suggest that first strategy provides biased results[4] therefore, we used the second strategy.
To our best knowledge there is no STATA package that deals with treatment effects in the context of multiple imputation, so we developed our own syntax using loops to estimate treatment effects and standard errors separately within each file and exported the results to Excel. At the next stage, we aggregated the exported results by implementing Rubin's rules for estimating the effects and standard errors from the multiply imputed datasets. [5] For the estimation *teffects ipw* we used the STATA built-in command and for the diagnostic procedures we used *tebalance summarize*. Models for medication and bedwetting alarm were not possible to estimate due to a very low number of children receiving these interventions.

For peer review only - http://bmjopen.bmj.com/site/about/guidelines.xhtml

We conducted diagnostics checking the plausibility of the imputation process by means of the *midiagplots* STATA command after datasets from the *ICE* package were successfully exported. Due to the large number of generated datasets, the diagnostics were based only on a subset of five randomly selected datasets (m=1,4,5,37,50) obtained from the MI procedure. The diagnostics were conducted with the assumption that the missing data mechanism was MAR. We compared the distributions of observed, imputed and completed values of each variable. The diagnostics revealed that they did not differ greatly which suggested that the imputation model was correctly specified. (The figures and tables including those diagnostic statistics are available on request).

Caveats of using propensity score based methods

Propensity score based methods are a group of methods used to estimate treatment effects from observational data by balancing treatment and non-treatment groups on the set of measured confounders (**X**). As Rosenbaum and Rubin (1983)[6] showed that this can be achieved by using propensity scores instead of all combinations of variables from **X**.

All propensity score methods rely on several assumptions of which the most crucial is the one known as the conditional independence assumption (CIA). It consists of two parts: (a) the potential result of being treated (Y_1) and the potential result of remaining untreated (Y_0) are independent of the treatment group (G), after controlling for a vector of measured confounders **(X):** $Y_{\mathbf{0},} Y_1 \perp G|\boldsymbol{X}$ and (b) every case, (with any characteristic on vector **X**) has a positive chance of being included in either treatment group, which is $0<P(G=1|\mathbf{X})<1$.

Part (a) of CIA assumes that there are no unmeasured confounders. If vector **X** does not include all important confounders (those related both to the outcome and the treatment) (a) is not satisfied and causal inferences might be biased. Unfortunately, this is the untestable part of CIA and needs to be assumed. Part (b) of CIA implies that there must be an overlap between treated and non-treated groups with regards to their characteristics on vector **X** which means that any person with any characteristic on **X** within treatment group will have their counterpart in non treatment group[1]. In contrary to (a), part (b) of CIA concerning the overlap of treated and non-treated is testable and can be made by comparing distributions of propensity scores for both groups. We have done this by graphical examination of propensity scores estimated for both treatment groups (see figures S2-S7). This assumption was fulfilled to a satisfactory extent since the differences we observed in the distributions of propensity scores in treated and non-treated groups were minor.

It should be also added here that since in the current paper we relied on ATT, both parts of CIA are adopted in their weaker, less stringent, versions, This, weaker version of CIA requires for (a) only $Y_{\mathbf{0},} \perp G|\boldsymbol{X}$ to hold (which remains untestable) and for (b) that cases from the treatment group will have their counterparts in the untreated group (but the overlap in the opposite direction is not necessary now). As shown in figures S2-S7 this milder version of the assumption generally held in the data.

After applying methods using propensity scores the assessment of balance achieved between both compared groups is the final diagnostic step. It is also notable that the assessment of balance obtained as a result of applying inverse propensity score weighting returned satisfactory results.

For peer review only - http://bmjopen.bmj.com/site/about/guidelines.xhtml

References

1. Rosenbaum PR. Observational Study. In: Everitt BS, Howell DC, eds. Encyclopedia of Statistics in Behavioural Science Volume 3. Chichester: John Wiley & Sons, Ltd. 2005: 1451-1462.
2. Austin PC. An introduction to propensity score methods for reducing the effects of confounding in observational studies. Multivariate *Behav Res* 2011; 46(3): 399–424.
3. Monson RR. Occupational Epidemiology. 2nd Edition. CRC Press, Boca Raton, London, New York, Washington, DC, 1990.
4. Mitra R, Reiter JP. A comparison of two methods of estimating propensity scores after multiple imputation. *Stat Methods Med Res* 2016; 25(1): 188-204.
5. White IR, Royston P, Wood AM. Multiple imputation using chained equations: Issues and guidance for practice. *Statistics in Medicine* 2011; 30(4): 377-399.
6. Rosenbaum PR, Rubin DB, The central role of the propensity score in observational studies for causal effects. *Biometrika* 1983; 70: 41-55.

For peer review only

Table S1. Model variables for multiple imputation (MI) with numbers of missing cases per variable (n =1258).

	MODEL VARIABLES	Number of missing/ imputed cases by MI	Imputation model
	Confounders		
Variable number:	Child and family variables:		
1	Gender	0	none
2	Developmental level at 18 months (m)[1]	100	linear regression
3	Temperament - Activity at 24 m[2]	129	logistic
4	Temperament - Adaptability at 24 m[2]	135	logistic
5	Temperament - Intensity at 24 m[2]	170	logistic
6	Temperament - Mood at 24 m[2]	132	logistic
7	Temperament - Persistence at 24 m[2]	140	logistic
8	Prosocial behaviour score at 81m[3]	146	logistic
9	Hyperactivity score at 81m[3]	146	logistic
10	Emotional symptoms score at 81m[3]	145	logistic
11	Conduct problems score at 81 m[3]	144	logistic
12	Peer problems score at 81 m[3]	144	logistic
13	Total behavioral problems score at 81 m[3]	146	logistic
14	EAS temperament -Emotionality 69 m[4]	157	logistic
15	EAS temperament -Activity 69 m[4]	149	logistic
16	EAS temperament -Shyness 69 m[4]	149	logistic
17	EAS temperament -Sociability 69 m[4]	179	logistic
18	Toilet training has not been started by 24 m (parental report)	562	logistic

For peer review only - http://bmjopen.bmj.com/site/about/guidelines.xhtml

19	Verbal IQ 8 years (y)[5]	296	logistic
20	Performance IQ 8 y[5]	297	logistic
21	Full scale IQ 8 y[5]	303	logistic
22	Social communication 7.5 y[6]	21	logistic
23	Stressful life events (child related) 42 m[7]	137	logistic
24	Sleep problems at 42 m (parental report)	83	logistic
25	Child's general health at 81 m (parental report)	214	logistic
	Problems with urinating at 7.5 y (parental report)		
26	Frequency child shows signs of needing to urinate	<5	logistic
27	Going to toilet without reminder	<5	logistic
28	Child needs to dash to toilet to urinate	13	logistic
29	Bedwetting frequency (>= twice a week)	0	none
30	Daytime wetting	<5	logistic
	Problems with soiling at 7.5 y (parental report)		
31	Daytime soiling	<5	logistic
32	Nighttime soiling	7	logistic
	Socioeconomic indicators (parental report)		
33	Family size (3 children or more)	22	logistic
34	Crowding index at 85 m	143	logistic
35	Home ownership at 85 m	146	logistic
36	Difficulty affording elementary goods for mother at 85 m	141	logistic
37	Difficulty affording elementary goods for child at 85 m	186	logistic
38	No car at 33m	126	logistic
39	Social class at 3 y11 m - based on mother's occupation	659	logistic
40	Social class at 3 y11 m - based on father/partner's occupation	340	logistic

For peer review only - http://bmjopen.bmj.com/site/about/guidelines.xhtml

	Mother: (maternal report)		
42	Maternal education (reported in antenatal period)	32	logistic
43	Early parenthood	0	
44	Marital status of mother	139	logistic
45	Mother has paid work outside home since child born (85 m)	124	logistic
46	Mother has help looking after child (85 m)	168	logistic
47	Mother has help with housework (85 m)	165	logistic
48	Mother feels she is getting enough sleep (85 m)	144	logistic
49	Mother spends enough time with child (85 m)	158	logistic
50	Mother had stressful life events since child's 5th birthday	128	logistic
51	Mother's general health (73 m)	132	logistic
52	Maternal psychopathology – depression (73 m)	135	logistic
53	Maternal psychopathology - anxiety at (73 m)	142	logistic
54	Mother bedwetting history	41	logistic
60	Mother daytime wetting history	41	logistic
61	Parenting style nurturing	113	logistic
62	Parenting style discipline	95	logistic
63	Toilet training at 15 m[8]	73	logistic
64	Toilet training at 24 m[8]	109	logistic
65	Rules about smoking in house	141	logistic
66	Does anyone smoke in the house?	164	logistic
65	Father figure is natural father of child	154	logistic
	PSM - outcome variable:		
O1	Bedwetting at 9.5 y	213	logistic
	PSM - treatment (strategy) variables:		
S1	Lifting	0	
S2	Restricting drinks	0	

For peer review only - http://bmjopen.bmj.com/site/about/guidelines.xhtml

S3	Daytime toilet trips	0
S4	Rewarding	0
S5	Medication	0
S6	Alarm prevention	0
S7	Showing displeasure	0
S8	Protection pants	0

1. Maternal rating of developmental level was assessed at 18 months using a questionnaire developed by ALSPAC including items from the Denver Developmental Screening Test (Frankenburg et al. Pediatrics 1992; 89(1):91-97) and comprising four domains of development (Cronbach's alphas: fine motor=0.679; gross motor= 0.689; communication= 0.752 and social skills= 0.624). Scores on each domain were adjusted for age in weeks, standardized (using a linear regression model and extracting the residuals) and reversed where appropriate so that high values on all scores reflected a lower level of development. We adjusted for a total development score derived from the sum of the scores on each domain.
2. Mothers completed the Toddler Temperament Scale (TTS: Fullard, et al. Journal of Pediatric Psychology 1984; 9:205-216) when the study children were 24 months. The TTS comprises statements describing specific behaviours and mothers were asked to rate how often their child behaves in that way on a scale ranging from 1 (almost never) to 6 (almost always). The scale comprises nine temperament traits, but we restricted our analysis to five traits we found were associated with bedwetting in an earlier study (Joinson et al. 2009. Longitudinal and Life Course Studies 1:73-94): *activity* (□□= 0.593), *adaptability* (□□= 0.637), *intensity* (□□= 0.570), *mood* (□□= 0.672) and *persistence* (□□= 0.711).
3. Psychological problems were assessed when the child was aged 81 months using the Revised Rutter Parent Scale for Preschool Children, which is an extension of the Rutter behaviour scale (Elander & Rutter. Journal of Methods in Psychiatric Research 1996; 6:63–78). The questionnaire comprises 43 statements describing behaviours and mothers are asked to rate the extent to which each statement describes their child on a scale comprising the options 1 (certainly true), 2 (sometimes true) and 3 (not true). Responses were aggregated to create scores in five domains: *emotional difficulties*, *conduct difficulties*, *hyperactivity* and *prosocial behaviour*. High levels of problems are indicated by high scores on emotional difficulties, conduct difficulties and hyperactivity and low scores on the prosocial behaviour scale.
4. The Emotionality Activity Sociability (EAS) Questionnaire (Buss & Plomin. Temperament: Early developing personality traits. Hillsdale, NJ: Erlbaum. 1984) was administered when study children were 69 months. The questionnaire comprises 20 statements about behaviours and mothers rated the extent to which each statement describes their child on a scale ranging from 1 (not at all like) to 5 (exactly like). Scores on these items are combined to form four subscales (each comprising five items): *emotionality*, *activity level*, *shyness* and *sociability*.
5. IQ: Wechsler D. Manual for the Wechsler Intelligence Scale for Children. 3rd ed. San Antonio, TX: Psychological

For peer review only - http://bmjopen.bmj.com/site/about/guidelines.xhtml

Corporation; 1991.

6. When children were 7.5 years mothers completed the 12-item Social Communication Disorder Checklist (Skuse et al. Br J Psychiatry 2005;187:568-572). A score of 2 SD or more below the mean was selected as indicating impaired social communication.
7. Stressful life events were measured using a maternally reported questionnaire comprising 42 life events that was derived for ALSPAC using previous inventories as a basis for item selection (Barnet et al. J Psychosom Res 1983: 27, 313–320).
8. Toilet training: Mothers were asked: What do you think about toilet training for your child? Options were: "It is too early to start any toilet training yet", "I have just started toilet training" and "I have been toilet training for some time".

For peer review only

Assessing the adequacy of the propensity score models

Table S2 (i). Diagnostics summary for **LIFTING**

**N in unweighted dataset: treated = 159; untreated 1,099**	**Model adjusted for child and family variables**					**Model adjusted for child and family variables and other parental strategies**				
	**m=1**	**m=4**	**m=5**	**m=37**	**m=50**	**m=1**	**m=4**	**m=5**	**m=37**	**m=50**
**N of treated in weighted dataset:**	**630**	**628**	**629**	**629**	**629**	**632**	**635**	**632**	**637**	**639**
**N of untreated in weighted dataset:**	**628**	**630**	**629**	**629**	**629**	**626**	**623**	**627**	**621**	**619**

Figure S2. Overlap for models for **LIFTING** including child and family variables

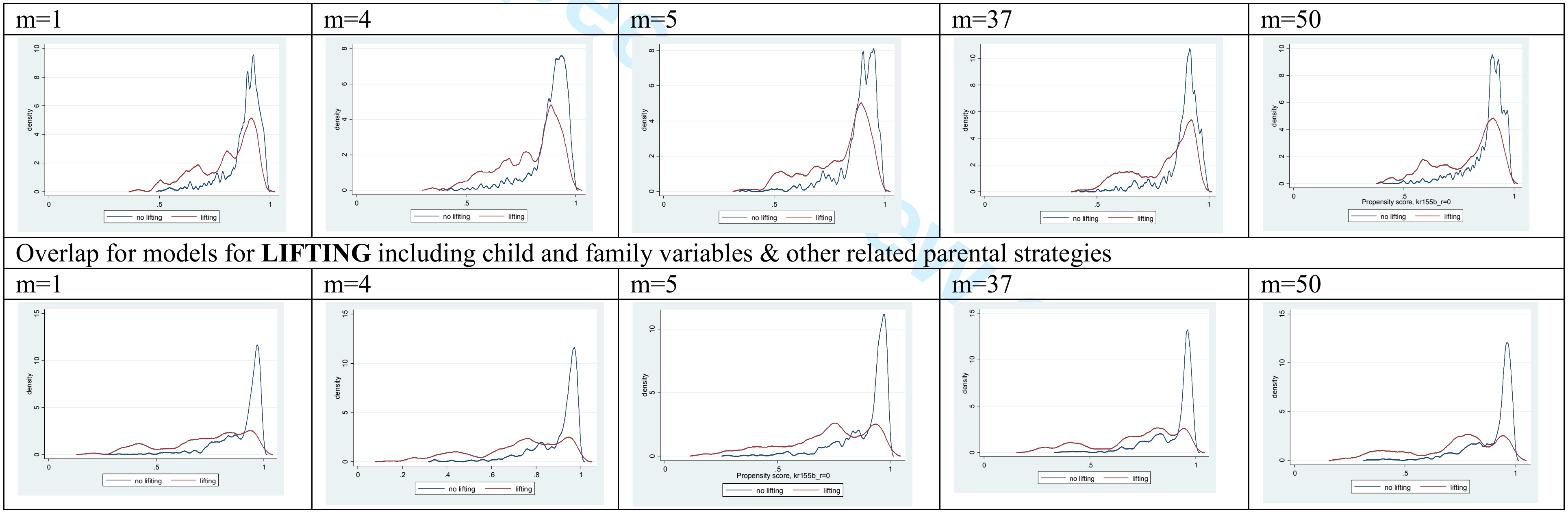

For peer review only - http://bmjopen.bmj.com/site/about/guidelines.xhtml

Table S2 (ii). Standardized differences on confounders after weighting

**CONFOUNDERS**	**Standardized differences in weighted sample in model adjusted for child and family variables**					**Standardized differences in weighted sample in model adjusted for child and family variables and other parental strategies**				
**Imputed dataset:**	**m=1**	**m=4**	**m=5**	**m=37**	**m=50**	**m=1**	**m=4**	**m=5**	**m=37**	**m=50**
**Child and family variables included as confounders:**										
Temperament - Activity at 24 m										
1. high score >28	.035	.026	.012	.003	.017	.054	.039	.033	.035	.043
Temperament - Adaptability at 24 m										
1. high score >17	-.002	-.009	.006	-.016	.004	.033	.014	.039	.039	.038
Temperament - Mood at 24 m										
1. high score >24	.019	-.002	.018	.017	.011	.073	.071	.063	.064	.048
Conduct problems score at 81 m										
1 abnormal (score >=4 (hyperproblems))	-.011	-.021	-.017	-.032	-.016	-.023	.003	.014	-.016	-.015
Total behavioural problems score at 81 m										
1 abnormal (score >=14 (hyper behavioral problems))	.009	-.004	-.008	.001	.007	.001	.007	.017	.002	.013
EAS temperament - activity at 69 m										
1 abnormal (score >=24 (hyper active))	-.011	.002	-.021	-.011	-.006	.041	.031	.033	.035	.036
EAS temperament -sociability at 69 m										
1 abnormal (score >=22 (antisocial))	.012	.012	.005	.013	.004	.012	.001	-.008	-.002	-.003
Verbal IQ										
1. 80<IQ<=120	.001	.022	-.001	.008	.013	-.026	-.027	-.028	.001	-.014
2. IQ>120	-.006	-.008	-.008	-.003	-.012	.015	.048	.018	-.001	.003
Performance IQ										
1. 80<IQ<=120	-.002	.018	-.001	.009	-.009	.024	.039	.029	.027	.016
2. IQ>120	-.003	-.002	-.001	-.007	-.006	-.028	-.019	-.027	-.057	-.043
Full scale IQ										
1. 80<IQ<=120	.001	.029	.022	.024	.020	-.008	.026	.028	.045	.021

For peer review only - http://bmjopen.bmj.com/site/about/guidelines.xhtml

2. IQ>120	.003	-.006	-.016	-.010	-.012	.013	.009	-.026	-.037	-.037
Social cognition										
1.(score>=8)	-.011	-.038	-.033	-.027	-.007	.000	-.036	-.004	.011	.014
Going to toilet without reminder										
1. never	-.006	.000	-.001	-.005	-.012	-.013	.001	.005	-.004	-.011
Bedtime wetting severity										
1. severe	.006	-.001	.007	.000	.017	.043	.041	.051	.061	.085
Daytime soiling										
1. yes	-.024	-.041	-.044	-.052	-.037	-.001	-.029	-.013	-.006	.003
Family size (3 or more children)										
1 # of children >=3	-.013	.005	-.002	.006	-.002	-.082	-.030	-.034	-.031	-.017
Social class - based on mother's occupation										
1. Managerial and technical	.004	-.007	.012	-.009	-.031	.025	.009	-.015	.011	-.018
2. Skilled non-manual	-.010	.004	.010	.011	-.002	-.026	-.024	-.014	.002	-.018
3. Skilled manual	-.019	-.024	-.003	-.029	.010	-.024	-.028	.000	.005	-.020
4. Partly skilled	-.003	-.005	-.022	.003	.019	.024	.037	.036	-.006	.044
5. Unskilled	.017	.019	.020	.014	.021	.037	.013	.029	.014	.036
Social class - based on father/partner's occupation										
1. Managerial and technical	.001	-.003	-.017	.002	-.005	.009	-.003	-.026	.013	.016
2. Skilled non-manual	.000	.012	.014	-.013	-.009	.002	-.002	-.002	-.020	-.043
3. Skilled manual	.003	.019	.024	.021	.030	.009	.023	.054	.0358	.059
4. Partly skilled	-.020	-.032	-.027	-.029	.002	-.044	-.020	-.053	-.044	-.017
5. Unskilled	.010	-.004	.007	.016	-.037	.015	.000	.034	.0199	-.016
Early parenthood										
1. Had child at age <19	.007	.005	.002	-.010	.000	.002	.001	.000	-.021	-.004
Amount of help mother has in looking after child at 85 m										
1. Too little	-.007	.002	.013	-.023	-.015	.030	.008	.023	-.001	.015
Mother state of health 73 m										
1. Mostly well & healthy	.030	.020	.032	.018	.025	.042	-.012	.029	.019	.034

For peer review only - http://bmjopen.bmj.com/site/about/guidelines.xhtml

2. Often unwell & hardly ever well	-.022	-.024	-.042	-.023	-.041	-.014	.037	-.034	.0159	-.011
Mother's history of bedwetting										
1. Mother had a problem when older than 5yo	-.009	-.003	-.018	-.011	-.018	-.034	-.020	-.028	-.014	-.023
Toilet training when child is 15 m										
1. Just started toilet training	.023	.002	-.005	.012	.009	.040	.022	.039	.031	.040
2. Toilet training for some time	.017	.016	.019	.015	.016	.051	.021	.019	.026	.018
Rules about smoking in household										
1. Smoking allowed in some rooms	-.021	-.017	-.017	-.010	-.014	-.005	-.014	-.014	.0196	.009
2. Smoking allowed	.004	.013	.014	.011	.010	-.002	.001	.009	.000	-.006
Father figure is natural father of child										
1. No / not living with father figure	-.005	-.003	.008	.021	.024	-.026	-.006	.011	.017	.021
**Related parental strategies variables (those with correlations>.45):**										
Restricting drinks										
1. yes						.000	.015	-.004	.021	.026

For peer review only

Table S3 (i). Diagnostics for **RESTRICTING DRINKS**

**N in unweighted dataset: treated = 403; untreated 855**	**Model adjusted for child and family variables**					**Model adjusted for child and family variables and other parental strategies**				
	**m=1**	**m=4**	**m=5**	**m=37**	**m=50**	**m=1**	**m=4**	**m=5**	**m=37**	**m=50**
**N of treated in weighted dataset:**	**631**	**631**	**633**	**631**	**633**	**627**	**630**	**630**	**629**	**629**
**N of untreated in weighted dataset:**	**628**	**627**	**625**	**627**	**625**	**631**	**628**	**628**	**630**	**630**

Figure S3. Overlap for models for **RESTRICTING DRINKS** including child and family variables

m=1	m=4	m=5	m=37	m=50
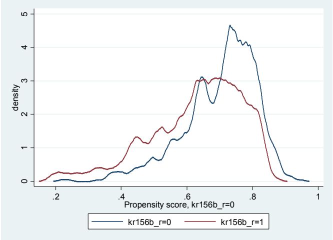	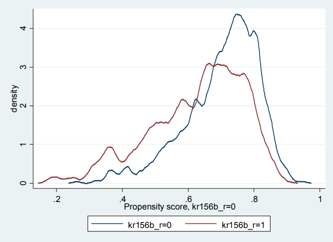	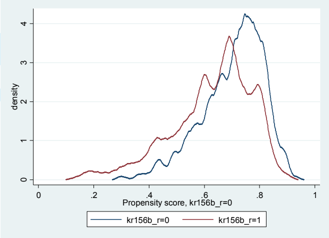	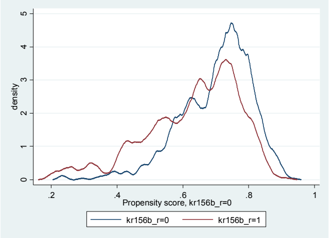	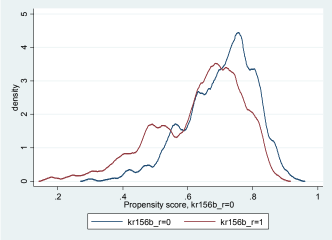
Overlap for models for **RESTRICTING DRINKS** including child and family variables & other related parental strategies				
m=1	m=4	m=5	m=37	m=50
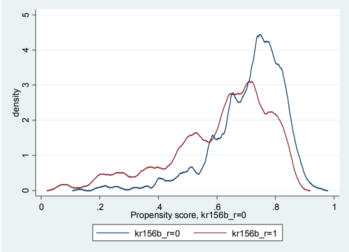	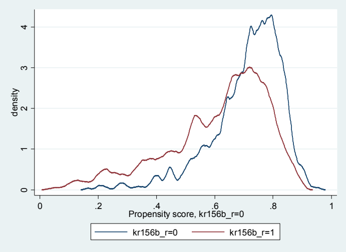	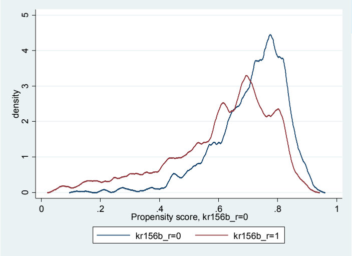	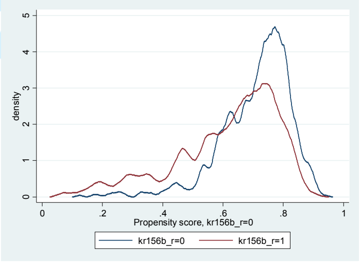	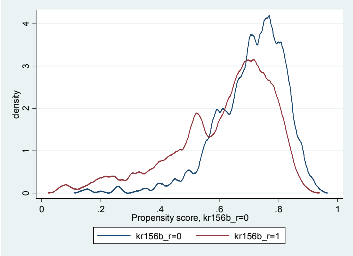

For peer review only - http://bmjopen.bmj.com/site/about/guidelines.xhtml

Table S3 (ii). Standardized differences on confounders after weighting

CONFOUNDERS	Standardized differences in weighted sample in model adjusted for child and family variables					Standardized differences in weighted sample in model adjusted for child and family variables and other parental strategies				
**Imputed dataset:**	**m=1**	**m=4**	**m=5**	**m=37**	**m=50**	**m=1**	**m=4**	**m=5**	**m=37**	**m=50**
**Child and family variables included as confounders:**										
Gender										
1. Female	*.017*	*.001*	*.010*	*-.004*	*.022*	*.025*	*.016*	*.003*	*.004*	*.027*
Temperament - Adaptability at 24 m										
1. High score >17	*.011*	*.036*	*.036*	*.009*	*.017*	*.020*	*.025*	*.041*	*.014*	*.010*
Emotional symptoms score at 81 m										
1. High score >=4	*-.007*	*.002*	*-.002*	*-.016*	*.006*	*-.108*	*.008*	*-.009*	*-.011*	*.011*
Conduct problems score at 81 m										
1. High score >=4	*.031*	*.041*	*.056*	*.016*	*.057*	*.042*	*.067*	*.068*	*.031*	*.075*
Total behavioral problems at 81 m										
1. High score >=14	*.014*	*.019*	*.031*	*.012*	*.027*	*.028*	*.038*	*.046*	*.030*	*.046*
EAS temperament -sociability at 69 m										
1 abnormal (score >=22 (antisocial))	*.029*	*.039*	*.034*	*.017*	*.020*	*.036*	*.056*	*.037*	*.031*	*.046*
Verbal IQ at 8 y										
1. 80<IQ<=120	*.019*	*.003*	*-.013*	*-.012*	*.015*	*-.001*	*-.014*	*-.023*	*-.024*	*-.012*
2. IQ>120	*-.014*	*-.014*	*-.001*	*.002*	*-.030*	*-.002*	*-.006*	*.002*	*.008*	*-.007*
Performance IQ at 8 y										
1. 80<IQ<=120	*-.022*	*.003*	*.000*	*.027*	*.009*	*-.045*	*-.019*	*-.019*	*-.003*	*-.013*
2. IQ>120	*-.005*	*-.017*	*-.011*	*.008*	*-.009*	*.018*	*-.005*	*.012*	*.033*	*.024*
Full scale IQ at 8 y										
1. 80<IQ<=120	*.015*	*.002*	*.016*	*.011*	*.018*	*.005*	*-.013*	*.019*	*-.002*	*-.015*
2. IQ>120	*-.012*	*-.014*	*-.020*	*.006*	*-.020*	*.012*	*.009*	*-.012*	*.032*	*.014*
Sleep problems at 42 m										
1 (6 sleep difficulties or more)	*-.027*	*-.001*	*-.015*	*-.039*	*-.003*	*-.039*	*-.001*	*-.026*	*-.049*	*-.010*
Child's general health at 81 m										

For peer review only - http://bmjopen.bmj.com/site/about/guidelines.xhtml

1. minor problems	*.012*	*-.007*	*-.007*	*.003*	*.017*	*.046*	*.021*	*.017*	*.028*	*.038*
2. ill/unwell	*-.025*	*-.009*	*-.020*	*-.010*	*-.037*	*-.011*	*-.005*	*-.001*	*.007*	*-.010*
Child goes to toilet without reminder at 7.5 y										
1. never	*-.002*	*.008*	*.002*	*-.006*	*.000*	*-.041*	*-.016*	*-.011*	*-.021*	*-.018*
Child needs to dash to toilet to urinate at 7.5 y										
1. straight away	*.014*	*.021*	*.042*	*.001*	*.007*	*-.052*	*-.028*	*-.008*	*-.056*	*-.042*
Daytime wetting at 7.5 y										
1.yes	*.043*	*.031*	*.055*	*.022*	*.027*	*.038*	*.029*	*.033*	*.008*	*.010*
Social class - based on mother's occupation										
1. managerial and technical	*.003*	*-.004*	*.005*	*.003*	*-.021*	*.007*	*-.012*	*-.005*	*-.017*	*-.032*
2. Skilled non-manual	*-.021*	*-.017*	*-.025*	*-.033*	*-.010*	*-.039*	*-.038*	*-.027*	*-.039*	*-.017*
3. Skilled manual	*.005*	*.014*	*.030*	*-.029*	*.002*	*.012*	*.019*	*.045*	*-.019*	*.013*
4. Partly skilled	*.021*	*.026*	*-.001*	*.030*	*.020*	*.013*	*.040*	*.012*	*.052*	*.052*
5. Unskilled	*-.001*	*-.014*	*.023*	*.041*	*.024*	*.024*	*.008*	*.007*	*.050*	*-.004*
Social class - based on father/partner's occupation										
1. Managerial and technical	*-.003*	*-.014*	*-.011*	*-.013*	*-.014*	*.007*	*-.022*	*.010*	*-.003*	*-.020*
2. Skilled non-manual	*-.001*	*-.001*	*-.001*	*-.008*	*-.004*	*.027*	*.021*	*.014*	*.022*	*.027*
3. Skilled manual	*-.013*	*-.012*	*-.015*	*-.003*	*-.006*	*-.066*	*-.042*	*-.042*	*-.050*	*-.028*
4. Partly skilled	*.000*	*.021*	*.024*	*.011*	*.013*	*.020*	*.036*	*.013*	*.024*	*.011*
5. Unskilled	*.026*	*-.006*	*.015*	*.014*	*.011*	*.033*	*.004*	*.029*	*.016*	*.016*
Early parenthood										
1.Having child at age <19	*-.025*	*.004*	*.008*	*-.010*	*.016*	*.000*	*.022*	*.029*	*.011*	*.039*
Amount of help mother has looking after child at 85m										
1. Too little	*-.010*	*.001*	*.021*	*.009*	*.015*	*.005*	*.014*	*.037*	*.026*	*.031*
Mother's bedwetting history										
1. Mother had bedwetting after 5 y	*-.009*	*-.014*	*.008*	*-.012*	*-.030*	*-.020*	*-.030*	*-.030*	*-.033*	*-.081*
Mother daytime wetting history										

For peer review only - http://bmjopen.bmj.com/site/about/guidelines.xhtml

1. Mother had daytime wetting after 5 y	*.000*	*.008*	*.025*	*.002*	*.025*	*.010*	*.014*	*.029*	*.015*	*.031*
Toilet training child at 15 m										
1. Just started	*-.011*	*.001*	*.007*	*.011*	*.012*	*-.018*	*-.020*	*-.029*	*-.011*	*-.017*
2. For some time	*-.006*	*-.002*	*.005*	*-.007*	*.004*	*.023*	*.015*	*.023*	*.012*	*.026*
Rules about smoking in household										
1. Smoking allowed in some rooms	*.012*	*-.006*	*.002*	*.008*	*.009*	*.021*	*.011*	*.024*	*.026*	*.032*
2. Smoking allowed	*.027*	*.024*	*.033*	*.033*	*.002*	*.018*	*.014*	*.021*	*.006*	*-.008*
Father figure is natural father of child										
1. No / not living with father figure	*.029*	*.015*	*.037*	*.046*	*.049*	*.033*	*.024*	*.038*	*.045*	*.028*
**Related parental strategies variables (with tetrachoric correlations > .45):**										
Showing displeasure										
1. Yes						*-.031*	*-.012*	*-.027*	*-.029*	*-.043*

For peer review only

For peer review only - http://bmjopen.bmj.com/site/about/guidelines.xhtml

Table S4 (i). Diagnostics for **DAYTIME TOILET TRIPS**

**N in unweighted dataset: treated = 119; untreated 1,139**	**Model adjusted for child and family variables**					**Model adjusted for child and family variables and other parental strategies**				
	**m=1**	**m=4**	**m=5**	**m=37**	**m=50**	**m=1**	**m=4**	**m=5**	**m=37**	**m=50**
**N of treated in weighted dataset:**	**636**	**630**	**645**	**637**	**639**	**651**	**659**	**659**	**649**	**657**
**N of untreated in weighted dataset:**	**622**	**628**	**613**	**621**	**619**	**607**	**599**	**599**	**609**	**601**

Figure S4. Overlap for models for **DAYTIME TOILET TRIPS** including child and family variables

m=1

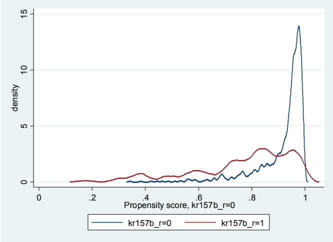

m=4

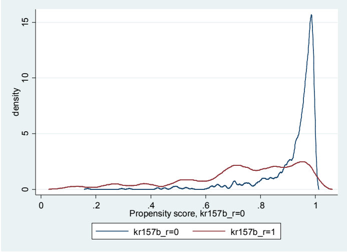

m=5

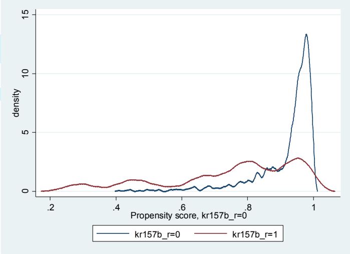

m=37

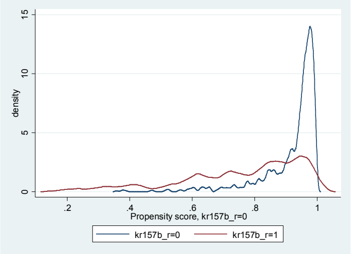

m=50

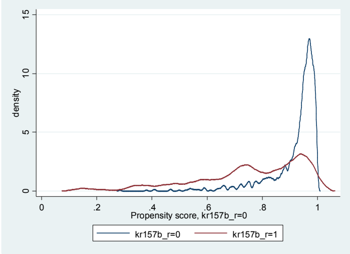

Overlap for models for **DAYTIME TOILET TRIPS** including child and family variables & other related parental strategies

m=1

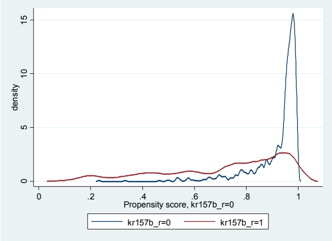

m=4

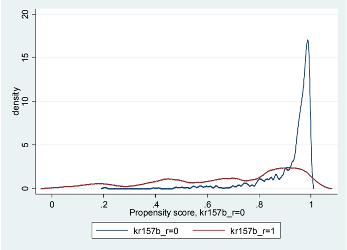

m=5

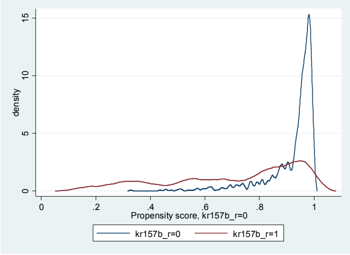

m=37

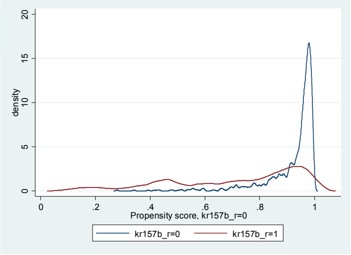

m=50

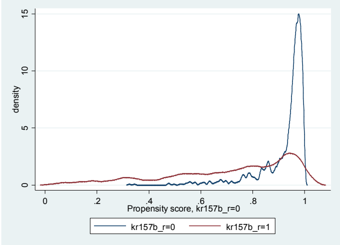

For peer review only - http://bmjopen.bmj.com/site/about/guidelines.xhtml

Table S4 (ii). Standardized differences on confounders after weighting

CONFOUNDERS	Standardized differences in weighted sample in model adjusted for child and family variables					Standardized differences in weighted sample in model adjusted for child and family variables and other parental strategies				
**Imputed dataset:**	**m=1**	**m=4**	**m=5**	**m=37**	**m=50**	**m=1**	**m=4**	**m=5**	**m=37**	**m=50**
**Child and family variables included as confounders:**										
Gender										
1. Female	*-.038*	*-.042*	*-.063*	*-.025*	*-.042*	*-.032*	*-.009*	*-.038*	*-.011*	*-.037*
Temperament - Activity at 24 m										
1. High score >28	*.018*	*.049*	*.015*	*-.004*	*.020*	*.022*	*.044*	*-.002*	*.001*	*.030*
Temperament - Adaptability at 24 m										
1. High score >17	*-.015*	*.024*	*-.012*	*-.047*	*-.022*	*-.008*	*.015*	*-.019*	*-.060*	*-.021*
Temperament - Mood at 24 m										
1. High score >24	*-.021*	*-.020*	*.004*	*-.028*	*-.023*	*-.063*	*-.080*	*-.025*	*-.070*	*-.050*
Hyperactivity score at 81 m										
1. High score>6	*.025*	*.066*	*.025*	*.012*	*.024*	*.018*	*.033*	*-.009*	*-.006*	*.017*
Emotional symptoms score at 81 m										
1. High score >=4	*-.002*	*.006*	*.007*	*-.020*	*.026*	*.004*	*.015*	*.000*	*-.007*	*.015*
Conduct problems score at 81 m										
1. High score >=4	*.052*	*-.003*	*.037*	*.030*	*.040*	*.059*	*.026*	*.062*	*.044*	*.054*
Total behavioral problems at 81 m										
1. High score >=14	*.034*	*.004*	*-.007*	*-.002*	*.010*	*.039*	*.011*	*-.030*	*-.004*	*.036*
EAS temperament - activity at 69 m										
1. High score >=24	*.051*	*.098*	*.047*	*.057*	*.078*	*.028*	*.064*	*.031*	*.043*	*.056*
EAS temperament -sociability at 69 m										
1. High score >=22	*.022*	*.038*	*-.013*	*.009*	*.035*	*.001*	*.042*	*-.016*	*.011*	*.015*
Verbal IQ at 8 y										
1. 80<IQ<=120	*-.004*	*-.026*	*-.008*	*-.012*	*.029*	*-.013*	*.011*	*.016*	*.031*	*.006*
2. IQ>120	*-.017*	*-.006*	*-.022*	*.015*	*-.004*	*-.041*	*-.060*	*-.049*	*-.032*	*-.023*

For peer review only - http://bmjopen.bmj.com/site/about/guidelines.xhtml

Performance IQ at 8 y										
1. 80<IQ<=120	*-.018*	*-.004*	*-.037*	*-.018*	*-.008*	*-.010*	*-.001*	*-.034*	*-.032*	*-.038*
2. IQ>120	*.009*	*-.003*	*-.007*	*.008*	*.031*	*.003*	*.002*	*-.009*	*.000*	*.011*
Full scale IQ at 8 y										
1. 80<IQ<=120	*-.007*	*.044*	*.021*	*-.008*	*.034*	*-.035*	*.037*	*.046*	*.006*	*-.007*
2. IQ>120	*-.010*	*-.026*	*-.028*	*-.002*	*.012*	*-.019*	*-.047*	*-.035*	*-.018*	*.003*
Social cognition										
1. High score>=8	*.006*	*.041*	*.052*	*.006*	*-.008*	*-.011*	*.028*	*.039*	*-.003*	*.023*
Stressful life events (child related) at 42 m										
1. 5 or more stress events	*.012*	*-.069*	*.034*	*.008*	*-.017*	*.016*	*-.069*	*.017*	*-.013*	*-.002*
Sleep problems at 42 m										
1. 6 sleep problems or more	*.042*	*.081*	*.078*	*.041*	*.047*	*.067*	*.119*	*.111*	*.082*	*.065*
Child's general health at 81 m										
1. Minor problems	*.019*	*-.030*	*-.023*	*.035*	*-.014*	*.023*	*-.040*	*-.038*	*.010*	*-.020*
2. Ill/unwell	*-.100*	*-.099*	*-.067*	*-.111*	*-.073*	*-.159*	*-.163*	*-.105*	*-.147*	*-.095*
Bedtime wetting severity at 7.5 y										
1. Severe	*.016*	*.065*	*.042*	*.051*	*.021*	*.058*	*.098*	*.089*	*.095*	*.076*
Daytime wetting at 7.5 y										
1. Yes	*.011*	*.003*	*.026*	*.011*	*.023*	*.065*	*.064*	*.081*	*.056*	*.098*
Child needs to dash to toilet to urinate at 7.5 y										
1. Yes, straight away	*-.007*	*-.001*	*-.005*	*.009*	*-.010*	*.014*	*.020*	*.002*	*.020*	*-.006*
Daytime soiling at 7.5 y										
1. Yes	*.015*	*.054*	*.042*	*.006*	*.060*	*.012*	*.034*	*.030*	*-.005*	*.066*
Night time soiling at 7.5 y										
1. Yes	*.039*	*.107*	*.040*	*.024*	*.063*	*.062*	*.131*	*.056*	*.069*	*.121*
Car access										
1. No car at household when child is 33 m	*.028*	*-.033*	*.083*	*.010*	*.062*	*.010*	*-.039*	*.063*	*.008*	*.053*
Affordability index for elementary goods (mother) at 85m										

For peer review only - http://bmjopen.bmj.com/site/about/guidelines.xhtml

1. Severe deprivation	*.006*	*.033*	*.013*	*.016*	*-.029*	*-.001*	*.021*	*.020*	*.019*	*-.029*
Social class - based on mother's occupation										
1. Managerial and technical	*.001*	*.006*	*.022*	*-.007*	*-.042*	*-.050*	*.027*	*.045*	*.002*	*-.020*
2. Skilled non-manual	*.001*	*-.027*	*-.003*	*.006*	*.008*	*.045*	*-.032*	*-.009*	*-.008*	*.009*
3. Skilled manual	*-.014*	*.000*	*.000*	*-.048*	*.033*	*-.038*	*.000*	*.000*	*-.086*	*-.002*
4. Partly skilled	*-.008*	*.014*	*.017*	*.018*	*.012*	*.020*	*.021*	*.004*	*.034*	*-.003*
5. Unskilled	*.032*	*.028*	*-.046*	*.020*	*.029*	*.024*	*.027*	*-.027*	*.051*	*.029*
Social class - based on father's occupation										
1. Managerial and technical	*-.020*	*-.079*	*-.013*	*-.015*	*-.039*	*-.048*	*-.106*	*-.028*	*-.027*	*-.034*
2. Skilled non-manual	*.008*	*.051*	*.030*	*.032*	*.043*	*.033*	*.043*	*.031*	*.066*	*.042*
3. Skilled manual	*-.001*	*.014*	*-.017*	*-.013*	*.003*	*-.019*	*-.028*	*-.024*	*-.051*	*-.029*
4. Partly skilled	*-.026*	*.007*	*.015*	*-.014*	*-.004*	*-.012*	*.097*	*.043*	*.010*	*.031*
5. Unskilled	*.032*	*.014*	*-.006*	*.014*	*-.008*	*.054*	*.007*	*-.007*	*.026*	*-.010*
Early parenthood										
1. Had child at age <19	*-.043*	*-.026*	*-.026*	*-.029*	*-.013*	*-.079*	*-.067*	*-.067*	*-.068*	*-.045*
Amount of help mother has in looking after child at 85 m										
1. Too little	*-.008*	*-.015*	*-.012*	*-.008*	*-.013*	*-.051*	*-.053*	*-.034*	*-.032*	*-.042*
Mother's general health at 73 m										
1. Mostly well & healthy	*.004*	*.090*	*.060*	*.029*	*.032*	*-.026*	*.033*	*.048*	*.003*	*.024*
2. Often unwell & hardly ever well	*-.025*	*-.002*	*-.059*	*-.027*	*-.081*	*-.004*	*.023*	*-.069*	*-.018*	*-.053*
Mother bedwetting history										
1. mother had a problem when older than 5yo	*.014*	*.043*	*.026*	*-.004*	*.016*	*-.021*	*-.010*	*.015*	*-.048*	*-.015*
Mother day time wetting history										
1. Mother had bedwetting after 5 y	*-.033*	*-.032*	*.019*	*-.011*	*.002*	*-.071*	*-.085*	*.024*	*-.039*	*-.013*
Toilet training when child 15m										
1. Just started	*.031*	*.021*	*-.014*	*.005*	*.018*	*.017*	*.011*	*.000*	*.009*	*.011*
2. For some time	*-.038*	*-.010*	*-.041*	*.001*	*-.093*	*-.064*	*-.018*	*-.038*	*-.004*	*-.088*
Rules about smoking in household										

For peer review only - http://bmjopen.bmj.com/site/about/guidelines.xhtml

1. Smoking allowed in some rooms	-.031	.028	-.001	-.002	-.006	-.034	.023	.028	.011	.000
2. Smoking allowed	-.030	.021	.014	-.011	.003	-.051	.000	.003	-.021	-.005
Father figure is natural father of child										
1. No / not living with father figure	.044	.023	.022	.023	.015	-.004	.008	.026	.029	.006
**Related parental strategies variables (those with correlations > .45):**										
Restricting drinks										
1. Yes						.104	.152	.133	.101	.146

For peer review only

Table S5 (i). Diagnostics for **REWARDING**

**N in unweighted dataset: treated = 169; untreated 1,089**	**Model adjusted for child and family variables**					**Model adjusted for child and family variables and other parental strategies**				
	**m=1**	**m=4**	**m=5**	**m=37**	**m=50**	**m=1**	**m=4**	**m=5**	**m=37**	**m=50**
**N of treated in weighted dataset:**	**635**	**629**	**627**	**629**	**631**	**615**	**619**	**612**	**606**	**610**
**N of untreated in weighted dataset:**	**623**	**629**	**631**	**629**	**627**	**643**	**639**	**646**	**652**	**647**

Figure S5. Overlap for models for **REWARDING** including child and family variables

m=1

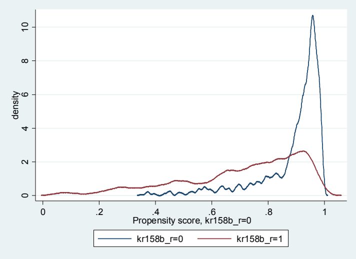

m=4

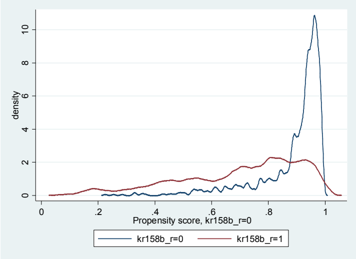

m=5

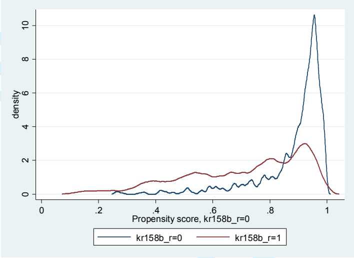

m=37

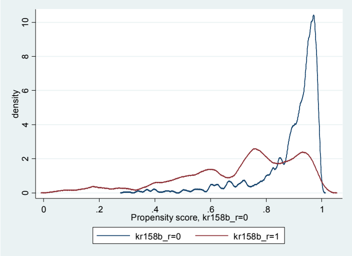

m=50

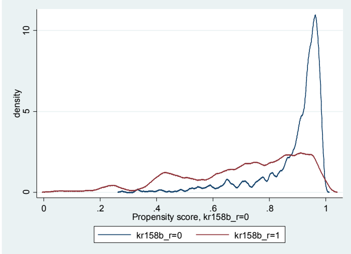

Overlap for models for **REWARDING** including child and family variables & other related parental strategies

m=1

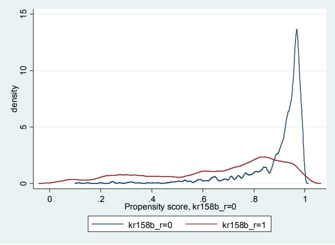

m=4

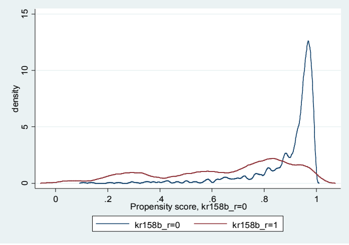

m=5

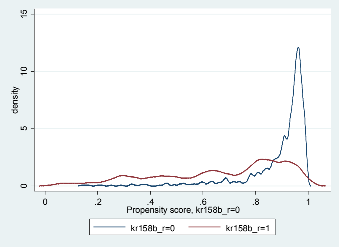

m=37

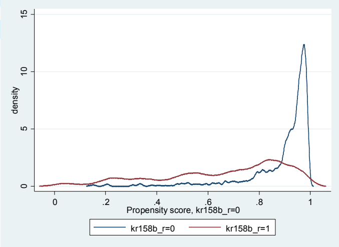

m=50

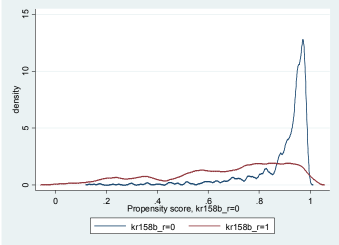

Table S5 (ii). Standardized differences on confounders after weighting

CONFOUNDERS	Standardized differences in weighted sample in model adjusted for child and family variables					Standardized differences in weighted sample in model adjusted for child and family variables and other parental strategies				
**Imputed dataset:**	**m=1**	**m=4**	**m=5**	**m=37**	**m=50**	**m=1**	**m=4**	**m=5**	**m=37**	**m=50**
**Child and family variables included as confounders:**										
Gender										
1. Female	*-.071*	*-.070*	*-.070*	*-.038*	*-.066*	*-.043*	*-.006*	*-.020*	*-.011*	*.004*
Temperament - Activity at 24 m										
1. High score >28	*.019*	*-.008*	*-.008*	*-.019*	*-.004*	*.032*	*.014*	*.006*	*-.011*	*.010*
Temperament - Mood at 24 m										
1. high score >24	*.040*	*.046*	*.046*	*.033*	*.050*	*.096*	*.030*	*.048*	*.067*	*.033*
Hyperactivity score at 81 m										
1. High score>6	*.034*	*-.023*	*-.023*	*.009*	*.019*	*.125*	*.030*	*.013*	*.036*	*.033*
Emotional symptoms score at 81 m										
1. High score >=4	*.006*	*-.062*	*-.061*	*-.061*	*-.026*	*-.040*	*.032*	*-.070*	*-.001*	*-.098*
Conduct problems score at 81 m										
1. High score >=4	*-.038*	*.005*	*.005*	*-.040*	*.005*	*-.008*	*.068*	*.098*	*.028*	*.082*
Total behavioral problems at 81 m										
1. High score >=14	*.014*	*-.053*	*-.053*	*-.044*	*.011*	*.054*	*.061*	*.014*	*.055*	*.081*
EAS temperament - activity at 69 m										
1 abnormal (score >=24 (hyper active))	*.067*	*.050*	*.050*	*.077*	*.073*	*.125*	*.106*	*.095*	*.125*	*.091*
EAS temperament -sociability at 69 m										
1. High score >=22	*-.007*	*-.019*	*-.019*	*.013*	*.022*	*.097*	*.049*	*.037*	*.078*	*.036*
Verbal IQ at 8 y										
1. 80<IQ<=120	*.049*	*-.010*	*-.010*	*-.026*	*.010*	*.018*	*.138*	*.045*	*-.029*	*.027*
2. IQ>120	*-.028*	*.030*	*.030*	*.028*	*-.009*	*-.046*	*-.123*	*-.049*	*.012*	*-.061*
Performance IQ at 8 y										

For peer review only - http://bmjopen.bmj.com/site/about/guidelines.xhtml

1. 80<IQ<=120	*.014*	*-.023*	*-.023*	*.042*	*-.047*	*.007*	*.066*	*-.026*	*.036*	*-.082*
2. IQ>120	*-.016*	*.004*	*.004*	*-.007*	*.009*	*-.142*	*-.018*	*-.068*	*-.075*	*-.055*
Full scale IQ at 8 y										
1. 80<IQ<=120	*.032*	*.004*	*.004*	*.040*	*.003*	*-.001*	*.023*	*.074*	*.076*	*.017*
2. IQ>120	*.001*	*.032*	*.032*	*-.002*	*.017*	*-.055*	*.008*	*-.088*	*-.044*	*-.069*
Social cognition										
1. Score>=8	*.041*	*-.016*	*-.016*	*-.005*	*.007*	*.049*	*.057*	*.028*	*.070*	*.036*
Stressful life events (child related) at 42 m										
1. 5 or more stress events	*-.013*	*-.005*	*-.005*	*.012*	*-.021*	*.031*	*-.035*	*-.013*	*.006*	*-.019*
Bedwetting severity										
1. Severe	*-.018*	*-.018*	*-.018*	*-.010*	*.004*	*-.053*	*-.065*	*-.071*	*-.093*	*-.060*
Daytime wetting										
1. Yes	*-.001*	*-.019*	*-.019*	*.008*	*-.010*	*.003*	*.081*	*.057*	*.057*	*.017*
Child goes to toilet without reminder at 7.5 y										
1. Never	*.000*	*.002*	*.002*	*-.003*	*.000*	*-.012*	*-.008*	*-.024*	*-.002*	*-.005*
Night time soiling at 7.5 y										
1. Yes	*.057*	*.039*	*.039*	*.005*	*-.007*	*.068*	*.082*	*.084*	*.081*	*-.006*
Affordability index for elementary goods (mother) at 85m										
1. Severe deprivation	*.032*	*.006*	*.006*	*-.025*	*.012*	*.099*	*.089*	*.068*	*.010*	*.076*
Social class - based on mother's occupation										
1. Managerial and technical	*.047*	*.008*	*.008*	*-.004*	*-.019*	*.002*	*.116*	*.085*	*.033*	*-.003*
2. Skilled non-manual	*-.057*	*-.023*	*-.023*	*-.010*	*-.026*	*-.078*	*-.169*	*-.143*	*-.053*	*-.077*
3. Skilled manual	*.071*	*-.018*	*-.018*	*-.025*	*.049*	*.042*	*.069*	*.012*	*-.063*	*-.001*
4. Partly skilled	*.002*	*.018*	*.018*	*.010*	*.003*	*.024*	*.021*	*.039*	*.034*	*.049*
5. Unskilled	*-.032*	*.003*	*.003*	*.043*	*.059*	*.073*	*.045*	*.036*	*.059*	*.086*
Social class - based on father's occupation										
1. Managerial and technical	*-.035*	*-.033*	*-.033*	*-.051*	*-.013*	*-.071*	*-.055*	*-.097*	*-.054*	*-.009*

For peer review only - http://bmjopen.bmj.com/site/about/guidelines.xhtml

2. Skilled non-manual	*.054*	*.038*	*.038*	*.035*	*.061*	*.083*	*.057*	*.087*	*.113*	*.096*
3. Skilled manual	*.030*	*.011*	*.011*	*.029*	*-.019*	*-.028*	*.045*	*.013*	*-.039*	*-.089*
4. Partly skilled	*-.028*	*-.020*	*-.020*	*.000*	*.007*	*.005*	*-.044*	*-.008*	*-.021*	*.026*
5. Unskilled	*-.006*	*.018*	*.018*	*.010*	*.000*	*.067*	*.007*	*.045*	*.050*	*.021*
Early parenthood										
1. Had child at age <19	*.011*	*.024*	*.024*	*.027*	*.044*	*.039*	*.035*	*.047*	*.057*	*.067*
Amount of help mother has in looking after child at 85m										
1. Too little	*.038*	*.069*	*.069*	*-.002*	*.022*	*.106*	*.125*	*.155*	*.131*	*.134*
Mother thinks she spends enough time with child (85m)										
1. No	*.010*	*.012*	*.012*	*.025*	*.032*	*.009*	*.027*	*-.027*	*.032*	*.011*
Mother's state of health 73m										
1. Mostly well & healthy	*.062*	*.023*	*.023*	*.040*	*.040*	*.090*	*-.034*	*.024*	*.033*	*.063*
2. Often unwell & hardly ever well	*-.048*	*-.056*	*-.056*	*-.032*	*-.027*	*.034*	*.022*	*-.067*	*.041*	*.049*
Mother bedwetting history										
1. Mother had bedwetting after 5 y	*.011*	*-.002*	*-.002*	*.037*	*.031*	*.058*	*-.034*	*.011*	*.050*	*.044*
Mother's daytime wetting history										
1. Mother had daytime wetting after 5 y	*.002*	*-.056*	*-.056*	*-.009*	*.013*	*.052*	*.059*	*.009*	*.055*	*.073*
Toilet training when child 15m										
1. Just started	*-.024*	*-.028*	*-.028*	*.009*	*.009*	*.065*	*-.048*	*.011*	*.023*	*.010*
2. For some time	*.005*	*.000*	*.000*	*.010*	*.024*	*.006*	*.023*	*.026*	*.042*	*.047*
**Related parental strategies variables (those with correlations > .45):**										
Restricting drinks										
1. Yes						*-.081*	*-.067*	*-.051*	*-.099*	*-.097*
Alarm prevention										
1. Yes						*-.219*	*-.098*	*-.202*	*-.168*	*-.187*

For peer review only - http://bmjopen.bmj.com/site/about/guidelines.xhtml

Table S6 (i). Diagnostics for **SHOWING DISPLEASURE**

N in unweighted dataset: treated = 55; untreated 1,203	Model adjusted for child and family variables					Model adjusted for child and family variables and other parental strategies				
	m=1	m=4	m=5	m=37	m=50	m=1	m=4	m=5	m=37	m=50
N of treated in weighted dataset:	625	627	631	623	626	629	625	628	619	622
N of untreated in weighted dataset:	633	631	628	635	631	629	633	630	639	636

Figure S6. Overlap for models for **SHOWING DISPLEASURE** including child and family variables

m=1	m=4	m=5	m=37	m=50
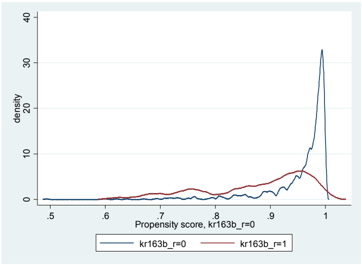	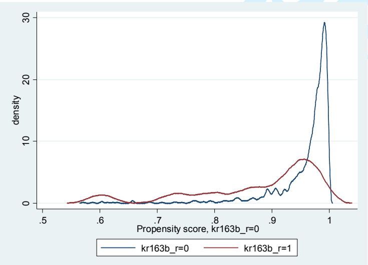	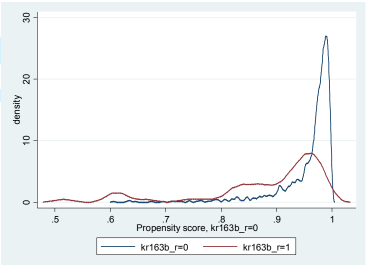	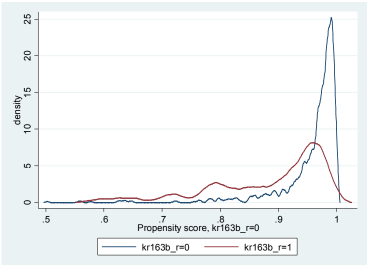	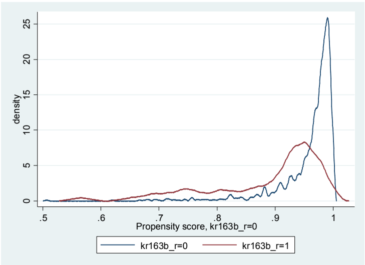
Overlap for models for **SHOWING DISPLEASURE** including child and family variables & other related parental strategies				
m=1	m=4	m=5	m=37	m=50
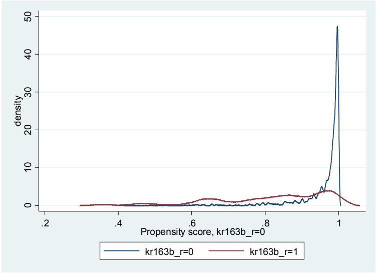	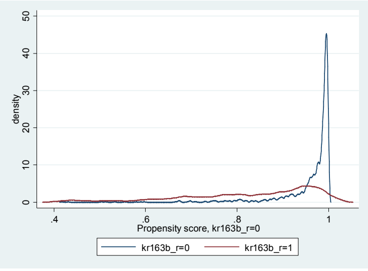	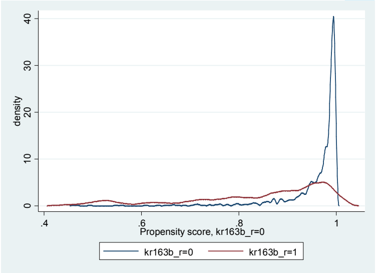	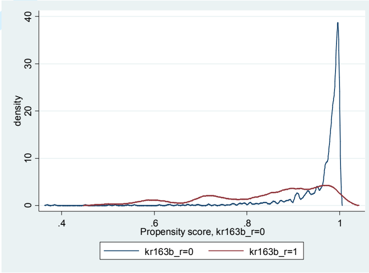	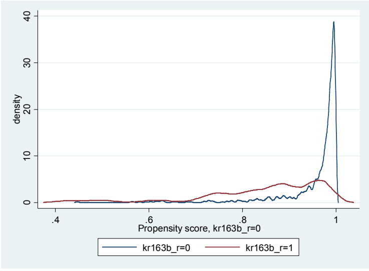

For peer review only - http://bmjopen.bmj.com/site/about/guidelines.xhtml

Table S6 (ii). Standardized differences on confounders after weighting

CONFOUNDERS	Standardized differences in weighted sample in model adjusted for child and family variables					Standardized differences in weighted sample in model adjusted for child and family variables and other parental strategies				
**Imputed dataset:**	**m=1**	**m=4**	**m=5**	**m=37**	**m=50**	**m=1**	**m=4**	**m=5**	**m=37**	**m=50**
**Child and family variables included as confounders:**										
Gender										
1. Female	*.013*	*.043*	*.030*	*.020*	*.034*	*-.004*	*.049*	*.031*	*-.006*	*.020*
Temperament - Mood at 24 m										
1. High score >24	*-.029*	*-.010*	*-.013*	*.000*	*-.009*	*.009*	*.015*	*.001*	*.026*	*.008*
Hyperactivity score at 81 m										
1. High score>6	*.003*	*.006*	*.009*	*-.010*	*.002*	*-.027*	*-.029*	*-.012*	*-.049*	*.007*
Emotional symptoms score at 81 m										
1. High score >=4	*-.010*	*.000*	*-.014*	*.010*	*-.003*	*-.037*	*-.029*	*-.050*	*-.001*	*-.012*
Conduct problems score at 81 m										
1. High score >=4	*.033*	*.044*	*.037*	*.013*	*.030*	*.009*	*.046*	*.044*	*-.011*	*.035*
Total behavioral problems at 81 m										
1. High score >=14	*.008*	*.011*	*.015*	*.003*	*-.008*	*-.025*	*-.023*	*.006*	*-.028*	*-.010*
EAS temperament - activity at 69 m										
1. High score >=24	*.014*	*-.007*	*.002*	*.016*	*-.007*	*.021*	*-.023*	*-.021*	*.011*	*-.010*
EAS temperament -sociability at 69m										
1. High score >=22	*.037*	*.048*	*.045*	*.060*	*.060*	*.062*	*.076*	*.063*	*.095*	*.098*
Verbal IQ ay 8 y										
1. 80<IQ<=120	*-.001*	*.013*	*.000*	*-.012*	*-.007*	*-.011*	*-.016*	*-.015*	*-.044*	*-.014*
2. IQ>120	*-.011*	*-.014*	*-.012*	*.008*	*.003*	*-.005*	*.010*	*.004*	*.034*	*.009*
Performance IQ at 8 y										
1. 80<IQ<=120	*.020*	*-.013*	*.016*	*.005*	*-.007*	*.025*	*-.048*	*.008*	*-.007*	*-.002*
2. IQ>120	*-.035*	*-.031*	*-.032*	*-.015*	*-.017*	*-.028*	*-.017*	*-.022*	*.012*	*-.017*
Full scale IQ at 8 y										

For peer review only - http://bmjopen.bmj.com/site/about/guidelines.xhtml

1. 80<IQ<=120	.018	.016	.018	.006	.004	.006	-.014	-.008	-.034	-.005
2. IQ>120	-.021	-.029	-.019	-.007	-.010	.002	.004	.003	.044	-.001
**Social cognition**										
1.(score>=8)	-.001	.013	-.010	.018	-.007	.004	.024	.000	.031	.006
**Stressful life events (child related) at 42 m**										
1. 5 or more stress events	-.034	-.008	-.009	-.019	-.025	.006	.011	-.022	-.057	-.001
**Sleep problems as 42 m**										
1. 6 sleep problems or more	-.005	.001	-.033	-.025	.000	-.040	-.018	-.062	-.083	-.030
**Child's general health at 81 m**										
1. Minor problems	.007	-.006	.017	.014	.009	.001	.005	.024	.043	-.012
2. Ill/unwell	-.058	-.042	-.032	-.060	-.057	-.040	-.049	-.006	-.064	-.011
**Child goes to toilet without reminder at 7.5 y**										
1. Never	-.033	-.011	-.027	-.016	.002	-.072	-.038	-.020	-.032	-.007
**Bedwetting severity at 7.5 y**										
1. Severe	.001	-.038	-.013	-.003	-.015	.005	-.058	-.016	-.002	-.029
**Daytime wetting at 7.5 y**										
1. Yes	-.025	-.018	.011	-.018	-.014	-.036	-.011	-.019	-.052	-.035
**Family size**										
1. 3 or more children	.003	-.002	-.001	-.028	-.026	.068	.014	.037	.034	-.009
**Affordability index for elementary goods (mother) at 85m**										
1. Severe deprivation	.002	.008	.011	.014	.016	.031	.051	.021	.041	.008
**Social class - based on mother's occupation**										
1. Managerial and technical	-.017	-.033	-.038	-.021	-.016	-.012	-.052	-.035	-.057	.005
2. Skilled non-manual	.014	.022	.011	.000	-.006	-.003	-.013	.002	-.005	-.024
3. Skilled manual	.031	.029	.037	.014	.018	.025	.028	.039	.005	.011
4. Partly skilled	-.001	.021	-.007	.019	.005	-.009	.032	-.005	.074	.008
5. Unskilled	.016	-.014	.048	.003	.033	.007	-.004	.064	.009	.035
**Social class - based on father's**										

For peer review only

For peer review only - http://bmjopen.bmj.com/site/about/guidelines.xhtml

occupation										
1. Managerial and technical	*-.001*	*-.012*	*-.019*	*.007*	*.001*	*.025*	*.019*	*.017*	*.024*	*.015*
2. Skilled non-manual	*.014*	*-.016*	*-.006*	*-.001*	*-.015*	*.028*	*.015*	*.007*	*.027*	*.004*
3. Skilled manual	*-.025*	*.008*	*.007*	*.006*	*-.008*	*-.073*	*-.044*	*-.036*	*-.042*	*-.031*
4. Partly skilled	*.018*	*.027*	*.052*	*-.004*	*.030*	*.042*	*.026*	*.053*	*.007*	*.028*
5. Unskilled	*.027*	*.004*	*.000*	*.000*	*.000*	*.023*	*.017*	*.005*	*.000*	*.000*
Early parenthood										
1. Had child at age <19	*.000*	*-.023*	*.008*	*-.011*	*.000*	*.047*	*-.041*	*.024*	*.028*	*.035*
Amount of help mother has in looking after child at 85 m										
1. Too little	*-.024*	*-.006*	*-.007*	*-.035*	*-.021*	*-.033*	*-.012*	*.030*	*-.048*	*.006*
Mother's general health 73 m										
1. Mostly well & healthy	*-.020*	*-.009*	*-.023*	*-.011*	*-.015*	*-.044*	*-.050*	*-.043*	*-.010*	*-.012*
2. Often unwell & hardly ever well	*.010*	*.029*	*.009*	*.023*	*-.006*	*.034*	*.063*	*.025*	*.055*	*.024*
Mother's daytime wetting history										
1. Mother had daytime wetting after 5 y	*.004*	*.018*	*.005*	*-.018*	*.029*	*.010*	*.016*	*-.001*	*-.005*	*.046*
Toilet training when child 15m										
1. Just started	*.034*	*.037*	*.034*	*.026*	*.029*	*.013*	*.042*	*.026*	*.014*	*.025*
2 .For some time	*.000*	*-.016*	*.007*	*-.004*	*.017*	*.061*	*.015*	*.040*	*.066*	*.057*
Rules about smoking in household										
1. Smoking allowed in some rooms	*.021*	*.016*	*.002*	*.011*	*.020*	*.014*	*-.014*	*-.006*	*-.010*	*.023*
2. Smoking allowed	*-.003*	*-.007*	*-.020*	*-.031*	*-.052*	*-.029*	*.002*	*-.044*	*-.043*	*-.052*
Father figure is natural father of child										
1. No / not living with father figure	*.020*	*.017*	*-.001*	*.008*	*.027*	*.035*	*-.004*	*-.041*	*.024*	*-.007*
**Related parental strategies variables (those with correlations > .45):**										
Restricting drinks										
1.yes						*.023*	*.003*	*.008*	*-.014*	*-.007*

For peer review only - http://bmjopen.bmj.com/site/about/guidelines.xhtml

Table S7 (i). Diagnostics for **PROTECTION PANTS**

**N in unweighted dataset: treated = 98; untreated =1,160**	**Model adjusted for child and family variables**				
	**m=1**	**m=4**	**m=5**	**m=37**	**m=50**
**N of treated in weighted dataset:**	**637**	**638**	**629**	**621**	**626**
**N of untreated in weighted dataset:**	**621**	**620**	**629**	**637**	**632**

Figure S7. Overlap for models for **PROTECTION PANTS** including child and family variables

m=1	m=4	m=5	m=37	m=50
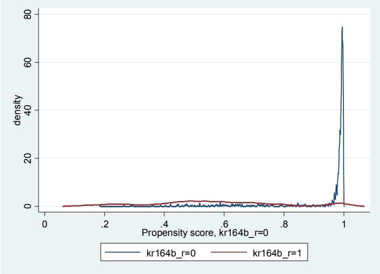	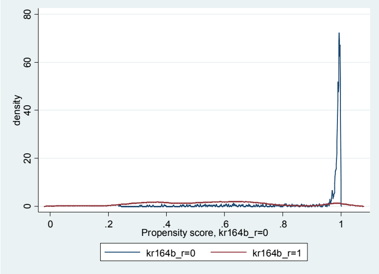	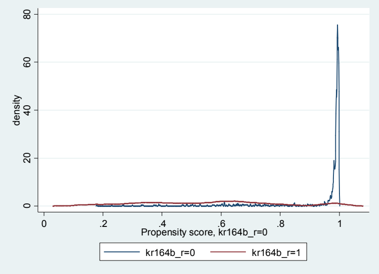	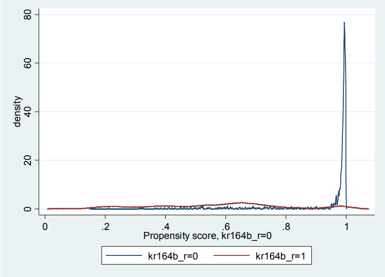	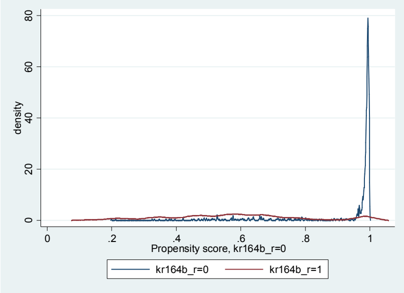

No other strategy was correlated with Protection pants strategy above the agreed level. Therefore in this case the above table includes only one row of figures.

For peer review only

Table S7 (ii). Standardized differences on confounders after weighting

**CONFOUNDERS**	**Standardized differences in weighted sample in model adjusted for child and family variables**				
**Imputed dataset:**	**m=1**	**m=4**	**m=5**	**m=37**	**m=50**
**Child and family variables included as confounders:**					
Gender					
1. Female	*.042*	*.028*	*.087*	*.041*	*.086*
Temperament - Adaptability at 24 m					
1. High score >17	*.000*	*.036*	*.053*	*.010*	*.021*
Temperament - Mood at 24 m					
1. High score >24	*-.030*	*-.048*	*-.013*	*.007*	*-.011*
Conduct problems score at 81 m					
1. High score >=4	*-.014*	*.010*	*-.022*	*.037*	*.058*
Total behavioral problems at 81 m					
1. High score >=14	*-.067*	*-.005*	*-.085*	*.048*	*.015*
EAS temperament - activity at 69 m					
1. High score >=24	*.015*	*-.018*	*-.006*	*.039*	*-.013*
EAS temperament -sociability at 69 m					
1. High score >= 22	*.038*	*.058*	*.052*	*-.043*	*.014*
Social cognition					
1. Score>=8	*-.025*	*-.008*	*-.013*	*-.014*	*-.009*
Full scale IQ at 8 y					
1. 80<IQ<=120	*-.016*	*-.059*	*.005*	*.072*	*.022*
2. IQ>120	*.032*	*.116*	*.037*	*.006*	*.078*
Verbal IQ at 8 y					
1. 80<IQ<=120	*-.090*	*-.141*	*-.074*	*-.065*	*-.086*
2. IQ>120	*.058*	*.130*	*.057*	*.053*	*.109*
Performance IQ at 8 y					
1. 80<IQ<=120	*-.139*	*-.176*	*-.130*	*.016*	*-.002*
2. IQ>120	*.072*	*.201*	*.142*	*.037*	*.086*

For peer review only - http://bmjopen.bmj.com/site/about/guidelines.xhtml

Stressful life events (child related) at 42 m					
1. 5 or more stressful events	*.041*	*-.008*	*.030*	*-.039*	*-.034*
Sleep problems as 42 m					
1. 6 sleep problems or more	*.001*	*.000*	*.019*	*.026*	*-.008*
Child's general health at 81m					
1. Minor problems	*.029*	*.012*	*.006*	*.093*	*.056*
2. Ill/unwell	*-.007*	*-.057*	*.040*	*-.010*	*.011*
Bedwetting severity at 7.5 y					
1. Severe	*.010*	*.012*	*.000*	*-.012*	*-.004*
Daytime wetting at 7.5 y					
1. Yes	*.024*	*.022*	*.041*	*-.022*	*-.022*
Daytime soiling					
1. Yes	*-.058*	*-.087*	*-.043*	*-.030*	*-.007*
Child needs to dash to toilet to urinate at 7.5 y					
1. Yes, straight away	*-.005*	*-.031*	*.002*	*.019*	*-.020*
Affordability index for elementary goods (mother) at 85m					
1. Severe deprivation	*.055*	*.071*	*.088*	*.048*	*.074*
Rules about smoking in household					
1. Smoking allowed in some rooms	*.039*	*.007*	*-.044*	*.054*	*-.002*
2. Smoking allowed	*.072*	*.060*	*.053*	*.055*	*.048*
Father figure is natural father of child					
1. no / not living with father figure	*-.008*	*-.015*	*-.014*	*-.005*	*.001*
**Related parental strategies variables (those with correlations > .45):**					
None					

For peer review only - http://bmjopen.bmj.com/site/about/guidelines.xhtml

STROBE 2007 (v4) Statement—Checklist of items that should be included in reports of *cohort studies*

Section/Topic	Item #	Recommendation	Reported on page #
**Title and abstract**	1	(*a*) Indicate the study's design with a commonly used term in the title or the abstract	1
		(*b*) Provide in the abstract an informative and balanced summary of what was done and what was found	2
**Introduction**			
Background/rationale	2	Explain the scientific background and rationale for the investigation being reported	4-5
Objectives	3	State specific objectives, including any prespecified hypotheses	5
**Methods**			
Study design	4	Present key elements of study design early in the paper	6
Setting	5	Describe the setting, locations, and relevant dates, including periods of recruitment, exposure, follow-up, and data collection	6
Participants	6	(*a*) Give the eligibility criteria, and the sources and methods of selection of participants. Describe methods of follow-up	6
		(*b*) For matched studies, give matching criteria and number of exposed and unexposed	
Variables	7	Clearly define all outcomes, exposures, predictors, potential confounders, and effect modifiers. Give diagnostic criteria, if applicable	6, 10-11, & table S1
Data sources/ measurement	8*	For each variable of interest, give sources of data and details of methods of assessment (measurement). Describe comparability of assessment methods if there is more than one group	6, 10, table S1
Bias	9	Describe any efforts to address potential sources of bias	12-14 & supplementary materials
Study size	10	Explain how the study size was arrived at	6-7
Quantitative variables	11	Explain how quantitative variables were handled in the analyses. If applicable, describe which groupings were chosen and why	12-14
Statistical methods	12	(*a*) Describe all statistical methods, including those used to control for confounding	12-14 & supplementary materials
		(*b*) Describe any methods used to examine subgroups and interactions	

For peer review only

		(*c*) Explain how missing data were addressed	14 & supplementary materials
		(*d*) If applicable, explain how loss to follow-up was addressed	14 & supplementary materials
		(*e*) Describe any sensitivity analyses	14 & supplementary materials
**Results**			
Participants	13*	(a) Report numbers of individuals at each stage of study—eg numbers potentially eligible, examined for eligibility, confirmed eligible, included in the study, completing follow-up, and analysed	6-7
		(b) Give reasons for non-participation at each stage	6-7
		(c) Consider use of a flow diagram	
Descriptive data	14*	(a) Give characteristics of study participants (eg demographic, clinical, social) and information on exposures and potential confounders	8-9
		(b) Indicate number of participants with missing data for each variable of interest	14 & table S1
		(c) Summarise follow-up time (eg, average and total amount)	
Outcome data	15*	Report numbers of outcome events or summary measures over time	
Main results	16	(*a*) Give unadjusted estimates and, if applicable, confounder-adjusted estimates and their precision (eg, 95% confidence interval). Make clear which confounders were adjusted for and why they were included	16
		(*b*) Report category boundaries when continuous variables were categorized	
		(*c*) If relevant, consider translating estimates of relative risk into absolute risk for a meaningful time period	
Other analyses	17	Report other analyses done—eg analyses of subgroups and interactions, and sensitivity analyses	Supplementary materials (p. 3-4)
**Discussion**			
Key results	18	Summarise key results with reference to study objectives	19
**Limitations**			
Interpretation	20	Give a cautious overall interpretation of results considering objectives, limitations, multiplicity of analyses, results from similar studies, and other relevant evidence	19-23
Generalisability	21	Discuss the generalisability (external validity) of the study results	24
**Other information**			
Funding	22	Give the source of funding and the role of the funders for the present study and, if applicable, for the original study on	24

For peer review only

		which the present article is based	

*Give information separately for cases and controls in case-control studies and, if applicable, for exposed and unexposed groups in cohort and cross-sectional studies.

Note: An Explanation and Elaboration article discusses each checklist item and gives methodological background and published examples of transparent reporting. The STROBE checklist is best used in conjunction with this article (freely available on the Web sites of PLoS Medicine at http://www.plosmedicine.org/, Annals of Internal Medicine at http://www.annals.org/, and Epidemiology at http://www.epidem.com/). Information on the STROBE Initiative is available at www.strobe-statement.org.

For peer review only

Examining the effectiveness of parental strategies to overcome bedwetting: an observational cohort study

Journal:	*BMJ Open*
Manuscript ID	bmjopen-2017-016749.R1
Article Type:	Research
Date Submitted by the Author:	15-May-2017

Complete List of Authors:	Grzeda, Mariusz; University of Bristol School of Social and Community Medicine<br>Heron, Jon; University of Bristol, Centre for Academic Mental Health, ; University of Bristol. UK<br>Tilling, Kate; University of Bristol, Social Medicine<br>Wright, Anne; Evelina London Children's Hospital<br>Joinson, Carol; University of Bristol, Centre for Child and Adolescent Health; School of Social and Community Medicine, University of Bristol

**Primary Subject Heading**:	Paediatrics
Secondary Subject Heading:	Epidemiology
Keywords:	Bedwetting, prevention, child, cohort study, ALSPAC

SCHOLARONE™
Manuscripts

For peer review only - http://bmjopen.bmj.com/site/about/guidelines.xhtml

Examining the effectiveness of parental strategies to overcome bedwetting: an observational cohort study

Corresponding author: Carol Joinson, University of Bristol, Oakfield House, Oakfield Grove, Clifton, Bristol, BS8 2BN. Email address: Carol.Joinson@bristol.ac.uk, Phone: 0117 331 3360

Authors: Mariusz T Grzeda MSci[1], Jon Heron PhD[1], Kate Tilling PhD[1], Anne Wright MBBCh[2], Carol Joinson PhD[1]

Affiliations:

1. School of Social and Community Medicine, University of Bristol, Oakfield House, Oakfield Grove, Clifton, Bristol BS8 2BN, UK.
2. Evelina London Children's Hospital, St Thomas' Hospital, Westminster Bridge Road, London SE1 7EH.

Word count: 3671

For peer review only

ABSTRACT

Objective: To examine whether a range of common strategies used by parents to overcome bedwetting in 7½-year-old children (including lifting, restricting drinks before bedtime, regular daytime toilet trips, rewards, showing displeasure and using protection pants) are effective in reducing the risk of bedwetting at 9½ years.

Design: Prospective cohort study.

Setting: General community.

Participants: The starting sample included 1,258 children (66.7% boys and 33.2% girls) who were still bedwetting at 7½ years.

Outcome measure: Risk of bedwetting at 9½ years.

Results: Using propensity score based methods we found that two of the parental strategies used at 7½ years were associated with an increased risk of bedwetting at 9½ years, after adjusting the model for child and family variables and other parental strategies: lifting (risk difference= 0.106 [95% CI= 0.009–0.202] i.e. there is an 10.6% (0.9% to 20.2%) increase in risk of bedwetting at 9½ years among children whose parents used lifting compared with children whose parents did not use this strategy) and restricting drinks before bedtime (0.123 [0.021–0.226]). The effect of using the other parental strategies was in either direction (an increase or decrease in the risk of bedwetting at 9½ years) e.g. showing displeasure (-0.052 [-0.214 to 0.110). When we re-analysed the data using multivariable regression analysis, the results were mostly consistent with the propensity score-based methods.

Conclusion: These findings provide evidence that common strategies used to overcome bedwetting in 7½ year olds are not effective in reducing the risk of bedwetting at 9½ years. Parents should be encouraged to seek professional advice for their child's bedwetting rather than persisting with strategies that may be ineffective.

For peer review only

Keywords: Bedwetting, prevention, child, cohort study, ALSPAC.

Strengths and limitations of this study:

- Major strengths of this study are the availability of prospective data on bedwetting and a wide range of covariates associated with both bedwetting and the parental strategies in a large birth cohort.
- Use of propensity score-based methods with observational data makes it easier to assess whether observed confounding has been adequately eliminated.
- We did not separately examine whether the parental strategies are differentially effective for children with non-monosymptomatic and monosymptomatic enuresis.
- We did not have information on the onset or duration of the strategies to overcome bedwetting and, therefore, we were only able to assess strategies that were reported by parents as being used currently.
- There were very small numbers of parents using medication or bedwetting alarms so we were unable to examine the effectiveness of these interventions in this study.

For peer review only

INTRODUCTION

Attainment of bladder control is a major milestone in child development that marks the end of a period of toilet training that is sometimes prolonged and stressful for parents and children. Enuresis is the term used by the International Children's Continence Society (ICCS) to describe bedwetting in children aged 5 years or older after ruling out organic causes[1]. It is most common for children to become dry during the day before they remain dry at night, [2] with children usually attaining night-time bladder control between the ages of four and six years[3]. Although a significant proportion of children continue to wet the bed at school age, only a small proportion wet the bed at least twice a week, the frequency required for DSM-V diagnosis of enuresis. For example, bedwetting was reported in 15.5% of 7½ year olds in the Avon Longitudinal Study of Parents and Children (ALSPAC), but only 2.6% wet the bed twice a week or more at this age[4]. With increasing age, bedwetting becomes more socially unacceptable and is often met with intolerance and frustration from parents[5]. Bedwetting also places considerable practical and financial burdens on the family in terms of the extra workload of washing bed linen and the cost of protective pants[6]. The psychosocial implications of bedwetting among school age children include worries about participating in sleepovers and school trips, fear of detection, teasing from peers, a sense of being different from others, emotional distress and low self-esteem[6]. Achieving bladder control is, therefore, important for a child's health and wellbeing.

It is a common belief among parents that bedwetting will eventually resolve with age and, as a result, many parents delay seeking treatment for bedwetting until it is having is a considerable impact on the child and family[7]. There is evidence from randomised and quasi-randomised trials that treatment of bedwetting with an alarm or medication can be effective[8,9], but many parents are unaware that effective treatments for bedwetting are available[10,11]. Before seeking medical advice, parents often employ a range of simple

strategies aimed at overcoming bedwetting, the most common being restricting drinks before bed, lifting (removing the sleeping child from bed to empty the bladder in the toilet or potty), rewarding for being dry, regular daytime toilet trips, using protection pants and showing displeasure[10]. Caldwell et al.[12] conducted a systematic review of randomised (or quasi-randomised) trials of simple behavioural interventions that are often used by parents as first line interventions for bedwetting. The studies compared the interventions with an appropriate comparison group, comprising no active treatment, other behavioural interventions, or drugs either alone or in combination with other interventions. They found evidence that simple behavioural interventions (e.g. rewards, lifting) were more effective in promoting dryness than no intervention, but were inferior to treatments such as alarms and medication. The authors of the review noted, however, that most trials were small and of poor methodological quality and caution should be exercised in interpreting the findings.

Although the best method for drawing causal inferences about interventions is a randomized controlled trial (RCT), this type of study design is not always feasible or ethical. When an RCT is not possible, observational studies are the only available type of evidence. Causality is difficult to assess in such studies because of the possibility of confounding – where one or more variable(s) influence both the exposure and the outcome, and thus it appears that there is a causal link between them. One method to assess the effectiveness of an intervention whilst adjusting for known and measured confounders is by using methods based on propensity scores. The aim of this study is to apply propensity score-based methods to observational data from a birth cohort to examine the effectiveness of a range of parental strategies aimed at overcoming childhood bedwetting and to compare this with results using regression methods.

METHODS

Participants

The sample comprised participants from the Avon Longitudinal Study of Parents and Children. Detailed information about ALSPAC is available on the study website (http://www.bristol.ac.uk/alspac), which includes a fully searchable dictionary of available data (http://www.bris.ac.uk/alspac/researchers/data-access/data-dictionary). Pregnant women resident in the former Avon Health Authority in south-west England, having an estimated date of delivery between 1/4/91 and 31/12/92 were invited to take part, resulting in a cohort of 14,541 pregnancies and 13,973 singletons/twins (7,217 boys and 6,756 girls) alive at 12 months[13]. Ethical approval for the study was obtained from the ALSPAC Law and Ethics committee and local research ethics committees. Written informed consent was obtained after the procedure(s) had been fully explained.

Bedwetting at 7½ and 9½ years: The starting sample for this study comprised children who were still bedwetting at 7½ years. Parents were asked "How often does your child wet him/herself during the night?" and for both questions were given the response options 'Never'; 'Occasional accident but less than once a week'; 'About once a week'; '2-5 times a week'; 'Nearly every day; and 'More than once a day'. A total of 8,151 parents responded to this questionnaire and 1,258 children (15.4%) were still wetting the bed at 7½ years, comprising 840 (66.7%) boys and 418 (33.2%) girls. Of the 1,258 children, 215 (69.8% boys and 30.2% girls) wet the bed 'at least twice a week'. At age 9½ years the questions on frequency of bedwetting were repeated. Bedwetting data were provided for 8,101 children and 788 (9.7%) wet the bed at this age (120 of these children wet the bed at least twice a week). The proportion of children with bedwetting at 7½ years whose parents provided data at age 9½ was 83% (1,045 out of 1,258) and 47% (n= 493) were still wetting the bed at this age, comprising 357 (72.4%) boys and 136 (27.6%) girls.

For peer review only - http://bmjopen.bmj.com/site/about/guidelines.xhtml

Parental strategies to overcome bedwetting at 7½ years

A questionnaire was administered to parents when the study children were aged 7½ that contained a list of strategies aimed at overcoming bedwetting that were originally elicited from parents[10]. Parents were asked: "*Which of the following methods have you tried in the past or are you using now to try and help your child stop wetting the bed*?" We restricted our analysis to strategies that parents reported '*using now*' because there was no information on the onset or duration of strategies used in the past. The questionnaire explained that these strategies are not necessarily effective in overcoming bedwetting.

Rationale for using propensity score methods in this study

Baseline characteristics of children whose parents used particular strategies to overcome bedwetting (referred to as 'treated' hereafter) might be expected to differ systematically from those whose parents did not use the strategy ('non-treated'), giving rise to confounding. It is, therefore, necessary to account for these differences when estimating the effect of the parental strategy ('treatment') on bedwetting. This could be achieved through multivariable regression (adjusting for differences in baseline variables between treated and untreated participants). Propensity score matching (PSM) has been proposed as a more appropriate method to minimise confounding when estimating treatment effects using observational data[14]. A detailed description of our rationale for using propensity score-based methods in our study is provided in the online supplementary materials.

Statistical analysis

We used PSM based methods to assess the effectiveness of the parental strategies to overcome bedwetting and compared this with estimates derived using logistic regression.

For peer review only - http://bmjopen.bmj.com/site/about/guidelines.xhtml

Both of these analyses aimed to estimate the effect of each parental strategy (used by parents of children who wet the bed at 7 ½ years) on risk of bedwetting at 9½ years.

Propensity score-based model: We assessed the effectiveness of each strategy by examining the difference in risk of bedwetting at 9½ years between children receiving the parental strategy ('treated') versus those children not receiving the strategy ('non-treated'). The estimate we computed was the *average treatment effect for the treated* (ATT)[15] (see supplementary material for an explanation of our choice of ATT as the measure of treatment effect). In order to put the results of PSM analyses in context we report estimates obtained both from unadjusted models (without any confounders) and from models adjusted for the set of measured confounders.

We implemented the inverse probability weighting (IPW) method using the propensity score because there is evidence that this method is relatively less biased than other methods based on propensity scores [15,16]. Aside from this, in the reweighting based on propensity scores, as opposed to matching on propensity scores, no case is excluded from the analysis. The baseline variables we selected into the propensity score model were those that were associated with the treatments (parental strategies used at 7 ½ years) and outcome (bedwetting at 9 ½ years).

We used a two-stage procedure to derive the list of baseline variables to include in the model. Firstly, we examined two logistic regression models: in model 1 the outcome variable was predicted from all theoretically relevant variables relating to children and their environment (see table S1 in the supplementary materials for the list of all variables included) In model 2, the parental strategy was the outcome variable. In the second stage, we applied a threshold for selection of variables to the propensity score model. The variables we included were those that had associations (expressed in terms of odds ratios) with the outcome variables in both models of <0.90 or >1.20, thus allowing us to detect at least 'weak'

For peer review only - http://bmjopen.bmj.com/site/about/guidelines.xhtml

associations[17]. We estimated models with and without adjustment for the confounders. A detailed list of the confounding variables is provided in table S1. Parents frequently used multiple strategies concurrently (indicated by strong correlations between subsets of parental strategies – table 2). For this reason, at the second stage of analysis, we repeated the IPW models for every strategy adjusting for confounders and strategies that were correlated (correlation >0.45, see table 2) with the particular strategy used as the outcome variable in the model. Before analysing the treatment effects, we assessed the adequacy of the propensity score model using diagnostic procedures detailed in the online supplementary material (see tables and figures S2 – S7).

Logistic regression model: We also examined the effectiveness of the parental strategies to overcome bedwetting by using multivariable logistic regression to adjust for confounders. We applied logistic regression models to the same set of baseline variables used in the propensity score model and reported the results in the form of adjusted risk differences (rather than odds ratios) for ease of comparison with the results from the propensity score-based model. We estimated risk differences through the STATA *logit* command followed by *adjrr* command[18]. The latter command returned estimates equivalent to average treatment effects (see supplementary materials). In order to obtain robust estimates of the coefficients and their standard errors we used loops developed in STATA in exactly the same way as for propensity score model.

Missing data

Many of the potential confounders we considered had some missing data, thus reducing the sample size available for analyses. The number of missing cases on confounding variables ranged from 0 (gender) to 659 (mother's social class based on

For peer review only - http://bmjopen.bmj.com/site/about/guidelines.xhtml

occupation) (see table S1). There were no missing data on exposure (treatment) variables. There were 213 missing cases on the outcome variable (bedwetting at 9½ years).

The exact numbers describing the amount of missing data per variable is presented in the supplementary materials (table S1). To deal with the missing data, we used multiple imputation by chained equations[19] within ICE (Imputation by Chained Equations) STATA package v.14[20]. Full details of the methods used are provided in the supplementary materials.

For peer review only

For peer review only - http://bmjopen.bmj.com/site/about/guidelines.xhtml

RESULTS

Table 1 describes the characteristics of the study participants.

For peer review only

For peer review only - http://bmjopen.bmj.com/site/about/guidelines.xhtml

Table 1. Characteristics of the study participants at 7½ and 9½ years.

	7½ years (n=1,258)	9½ years (n=1,045)
Gender		
Female	33.2%	32.8%
Male	66.8%	67.2%
n	1,258	1,045
Social class[1]		
Non-manual (professional, managerial, skilled professions)	82.0%	82.3%
Manual (partly or unskilled occupations)	18.0%	17.7%
n	1,094	923
Home ownership[1]		
Owner/occupier	85.5%	87.5%
Rented accommodation	14.5%	12.5%
n	1,112	952
Car access[1]		
Yes	91.1%	91.8%
No	8.9%	8.2%
n	1,132	965
Maternal education[1]		
A-level or above	43.9%	46.9%

For peer review only - http://bmjopen.bmj.com/site/about/guidelines.xhtml

O-level	33.6%	33.4%
Certificate of secondary school / vocational/ none	22.5%	19.7%
n	1,226	1,021

1. These variables were derived from responses to a questionnaire completed by mothers during the antenatal period.

For peer review only

For peer review only - http://bmjopen.bmj.com/site/about/guidelines.xhtml

Figure 1 shows the prevalence of each strategy used by parents of 7½ year old children and the total number of strategies employed by parents. The most common strategies for overcoming bedwetting were restricting drinks, rewarding and lifting. Only a very small number of parents used medication or bedwetting alarms, so we were unable to examine the effectiveness of these interventions. Over 50% of parents did not use any of the strategies to overcome bedwetting, whilst over 26% of parents used only one strategy. Table 2 shows the associations between the strategies. The strongest correlations were found between lifting and restricting drinks and between regular daytime toilet trips and rewards.

For peer review only

Table 2. Associations between the parental strategies used to overcome bedwetting in children at 7½ years (n=1,258).

	Lifting child out of bed to use toilet	Restricting drinks before bedtime	Regular daytime toilet trips	Rewarding child for being dry	Showing displeasure when child wets bed	Using nighttime protection pants / nappies	Medication for bedwetting	Bedwetting alarm
Lifting	1.000							
Restricting drinks	0.509	1.000						
Daytime toilet trips	0.295	0.413	1.000					
Rewards	0.444	0.407	0.523	1.000				
Showing displeasure	0.236	0.456	0.323	0.345	1.000			
Protection pants	-0.038	-0.151	0.231	0.249	0.040	1.000		
Medication	0.238	0.164	0.416	0.423	0.016	0.296	1.000	
Bedwetting alarm	0.050	-0.099	0.230	0.492	-0.070	0.230	0.113	1.000

Note: The associations between strategies are tetrachoric correlation coefficients. These are correlation coefficients of binary variables. The tetrachoric correlation coefficient provides an estimate of what the correlation would be if the variables were measured on a continuous scale. The size of the tetrachoric correlation coefficient can be interpreted in the same way as a correlation coefficient between 2 continuous variables i.e. ‘0’ indicates no correlation and ‘1’ indicates perfect correlation.

All coefficients were computed on the baseline sample.

For peer review only - http://bmjopen.bmj.com/site/about/guidelines.xhtml

Table 3 shows the average treatment effects on treated for each parental strategy used at 7½ years on risk of bedwetting at 9½ years derived from the propensity score based analysis. The table also shows the average treatment effects derived from the logistic regression model. The coefficients in table 3 are estimated differences between the risk of bedwetting at 9½ years after receiving given 'treatment' (parental strategy) and the risk of bedwetting if they had remained 'untreated'. Figure 2 displays the results of both analyses as forest plots.

For peer review only

Table 3. Estimated differences in the risk of bedwetting at 9½ years in children whose parents used each strategy compared with those who did not use the strategy.

	Average treatment effect on treated (95% CI) based on inverse probability weighting using propensity score			Average treatment effect / adjusted risk difference (95% CI) based on logistic regression analysis		
	Empty (unadjusted) model[a]	Model adjusted for child and family variables[b]	Model adjusted for child and family variables and other parental strategies[c]	Empty (unadjusted) model[d]	Model adjusted for child and family variables[e]	Model adjusted for child and family variables and other parental strategies[f]
Lifting	0.251 (0.167–0.335)*	0.148 (0.059–0.237)*	0.106 (0.009–0.202)*	0.251 (0.167–0.335)*	0.172 (0.081–0.263)*	0.125 (0.029–0.221)*
Restricting drinks	0.173 (0.110–0.236)*	0.138 (0.071–0.205)*	0.123 (0.021–0.226)*	0.173 (0.110–0.236)*	0.147 (0.083–0.211)*	0.112 (0.045–0.180)*
Daytime toilet trips	0.198 (0.100–0.296)*	0.088 (-0.024–0.201)	0.068 (-0.047–0.182)	0.198 (0.100–0.296)*	0.087 (-0.024–0.198)	0.057 (-0.056–0.171)
Rewards	0.281 (0.201–0.361)*	0.141 (0.049–0.233)*	0.088 (-0.014–0.191)	0.281 (0.201–0.361)*	0.158 (0.062–0.254)*	0.143 (0.044–0.242)*
Showing displeasure	0.054 (-0.091–0.199)	0.009 (-0.146–0.164)	-0.052 (-0.214–0.110)	0.054 (-0.091–0.199)	0.009 (-0.134–0.151)	-0.047 (-0.187–0.093)
Protection pants	0.290 (0.182–0.398)*	-0.010 (-0.161–0.141)	-0.010 (-0.161–0.141)	0.290 (0.182–0.398)*	0.025 (-0.134–0.184)	0.025 (-0.134–0.184)

For peer review only - http://bmjopen.bmj.com/site/about/guidelines.xhtml

The estimates provided in this table are average treatment effects for each strategy. They are risk differences i.e. estimated differences between the risk of bedwetting at 9½ years after receiving given 'treatment' (i.e. parental strategy) and the risk of bedwetting if they had remained 'untreated'.

The estimates in this table are risk differences. We provide examples of how to interpret the risk differences below:

(1) The risk difference for 'restricting drinks' (0.123 [95% CI= 0.021-0.226]) shows that there is a 12.3% (2.1% to 22.6%) increase in risk of bedwetting at 9½ years among children whose parents used restricting drinks compared with children whose parents did not use this strategy.

(2) The risk difference for 'showing displeasure' (-0.052 [-0.214-0.11]) shows that there is a 5.2% reduction in the risk of bedwetting at 9 years among children whose parents show displeasure, but the 95% confidence interval indicates that this result could be in either direction (between a 21% reduced risk and 11% increased risk).

* Indicates risk differences that provide evidence for an *increase* in bedwetting associated with the parental strategy.

[a] *Empty model* column for propensity score based methods analysis shows unadjusted differences in risk of bedwetting between 'treated' and 'untreated' children. These coefficients are equivalent to bivariate regressions including bedwetting at 9 ½ years as an outcome variable and each strategy as a single predictor.

[b] *Model adjusted for child and family variables* represents average treatment effects on treated children (ATETs). These coefficients are estimated differences in risks of bedwetting in weighted samples. These are differences in risks adjusted for child and family variables that accounted for the differences between treated and untreated groups and between those with and without bedwetting at age 9 ½. The list of child and family variables was derived separately for every strategy on the basis of odds ratios (ORs) from regression analyses providing evidence of an association (ORs <0.90 or >1.20) both with the given strategy and with bedwetting at age 9 ½. A detailed list of child and family variables included in model for each parental strategy is provided in the online supplementary material (see tables S2 – S7).

[c] *Model adjusted for child and family variables and other parental strategies.* This is the column with coefficients adjusted for the child and family variables and for other parental strategies that were highly correlated (coefficient of tetrachoric correlation: >.45) with given strategy.

For peer review only - http://bmjopen.bmj.com/site/about/guidelines.xhtml

[d] *Empty model*: results of univariable logistic regression analyses including bedwetting at 9 ½ years as an outcome variable and the given strategy as a single predictor. To ensure direct comparisons with the output of the analysis using propensity score based methods, the results of regression analyses are expressed here in terms of risk differences instead of ORs.

[e] *Model adjusted for child and family variables.* This includes estimated differences in risk of bedwetting for given strategy obtained from logistic regression, also including child and family variables.

[f] *Model adjusted for child and family variables and other parental strategies.* Risk differences for given strategy obtained from logistic regression including child and family variables and other strategies associated with the strategy under examination.

For peer review only - http://bmjopen.bmj.com/site/about/guidelines.xhtml

Propensity score based results (inverse probability weighting)

The unadjusted propensity score based analysis shows that the parental strategies used at 7½ years are associated with an increase in the risk of bedwetting at 9½ years. Showing displeasure was the only strategy that was also associated with a small decrease in the risk of bedwetting in the unadjusted model. After applying the propensity score model adjusting for the child and family variables and then further adjusting for the other strategies, the adjusted treatment effects provided evidence that lifting and restricting drinks are associated with an increase in the risk of bedwetting at 9 ½ years. The adjusted results for the other strategies indicated that the effect could be in either direction (either an increase or decrease in the risk of bedwetting).

Logistic regression results

The results obtained from the logistic regression analysis were mostly consistent with the analysis using the propensity score-based methods i.e. both lifting and restricting drinks were associated with an increase in the risk of bedwetting at 9½ years in the fully adjusted model. Using rewards was also associated with an increased risk of bedwetting at 9½ years.

For peer review only - http://bmjopen.bmj.com/site/about/guidelines.xhtml

DISCUSSION

We examined a range of common strategies used by parents to overcome bedwetting and found that when these strategies were used with 7½ year-old children who wet the bed, they were not effective in reducing the risk of bedwetting at 9½ years. Parental strategies including lifting and restricting drinks before bedtime were associated with an increased risk of subsequent bedwetting. These were among the most common parental strategies used to overcome bedwetting in our study and there is evidence in a review that these strategies are frequently used by parents across different countries[10].

This is the first study, to our knowledge, to apply propensity score-based methods to examine the effectiveness of parental strategies to overcome bedwetting using observational data from a large birth cohort. Austin [14] discusses several reasons for preferring the use of propensity score based methods to regression models when estimating treatment effects using observational data. In particular, it is easier to assess whether observed confounding has been adequately eliminated using propensity score based methods. It is important to note some caveats associated with PSM-based methods and these are discussed in the supplementary materials. Another major strength of this study is the availability of a wide range of confounders in the ALSPAC dataset that are associated with bedwetting and with the parental strategies to overcome bedwetting. We did not separately examine whether the parental strategies are differentially effective for children with non-monosymptomatic (bedwetting with daytime wetting and/or lower urinary tract symptoms [21]) and monosymptomatic enuresis (bedwetting without these symptoms)[22]. There is evidence that frequent childhood bedwetting that is accompanied by daytime wetting is less likely to resolve with age[23,24]. If we had compared children with monosymptomatic and non-monosymptomatic enuresis this would necessarily have reduced of the number of cases included in the analysis, resulting in serious problems with estimation of the propensity

For peer review only - http://bmjopen.bmj.com/site/about/guidelines.xhtml

score-based models. We did, however, include a range of covariates in the analyses including frequency of bedwetting (high frequency= twice or more/week), daytime wetting, urgency and voiding postponement. All of these variables were important confounders in most of the models. We did not have information on the onset or duration of the strategies to overcome bedwetting and, therefore, we were only able to assess strategies that were reported by parents as being used currently. Due to this being a community-based, rather than a clinical, sample, there were very small numbers of parents using medication or bedwetting alarms. We were, therefore, unable to examine the effectiveness of these interventions in this study. Additionally, there was no information on which strategies parents initiated compared with those recommended by health care workers or other sources (e.g. the child's grandparents).

Caldwell *et al.*[12] concluded from their systematic review, that simple interventions such as lifting (or waking) could initially be tried as a strategies to overcome bedwetting, since such methods are "safe and are better than doing nothing". However, they cautioned that most of the trials they reviewed were small and of poor methodological quality[12]. Our findings are consistent with the current advice given in the NICE guidelines stating: "Neither waking nor lifting children and young people with bedwetting, at regular times or randomly, will promote long-term dryness" [25]. Although lifting appears to be sensible for promoting nighttime dryness, its effectiveness as an intervention for reducing or stopping bedwetting has previously been questioned. It has been suggested that lifting may inadvertently maintain bedwetting since this strategy encourages the child to empty the bladder without fully waking and, therefore, children are not learning to waken to the sensation of a full bladder [10,26]. However, we are not aware of any studies that have formally tested this proposed mechanism. The effectiveness of lifting requires further investigation in studies that are able to distinguish between children with more severe and less severe bedwetting. Lifting the child from their

For peer review only - http://bmjopen.bmj.com/site/about/guidelines.xhtml

bed to pass urine can occur with or without waking the child, but this distinction was not possible in our study since parents were not specifically asked whether they woke the child when lifting them to use the toilet or potty. Restricting drinks one hour before bedtime is often recommended especially drinks with diuretic properties[27] and drinks containing caffeine[25]. Current NICE guidelines state that adequate daily fluid intake and using the toilet at regular intervals throughout the day are important in the management of bedwetting[25]. We did not find evidence that encouraging regular daytime toilet trips resulted in a decreased risk of bedwetting at 9 ½ years, but our findings are based on parental reports rather than a detailed diary of toileting and fluid intake completed during treatment. Using positive (rewards) or negative reinforcements (showing displeasure) as strategies aimed at overcoming bedwetting may be problematic because rewarding outcomes that the child has no control over (i.e. a dry night) could lead children to feel that they have failed if they continue to wet the bed[28]. It is recommended to reward steps towards nighttime dryness that the child *does* have control over (e.g. going to the toilet before bed without prompting, drinking and passing urine at regular intervals throughout the day, helping to change sheets)[25]. Protection pants (nappies, pull-ups) are often recommended as useful short-term solution to supporting children during school trips and sleepovers. However, health professionals do not recommend prolonged use of protection pants because this may not help children to become dry in the long-term. Instead, parents are advised to consider using alternative bed protection such as waterproof sheets[25]. A trial of at least 2 nights in a row without protection pants is recommended for children under 5 years who are still bedwetting, but have been dry during the day for more than 6 months[25].

The majority of children experience a natural resolution of bedwetting with increasing age (spontaneous resolution rate is estimated to be 15% per year[29]). Parents and clinicians, therefore, often adopt a *'wait and see'* approach to childhood bedwetting, due to the common

For peer review only - http://bmjopen.bmj.com/site/about/guidelines.xhtml

belief that it will resolve with age[7]. There is increasing evidence, however, that children who experience frequent bedwetting and those who have bedwetting with additional daytime bladder symptoms are at risk of their problems persisting into adolescence. A cross-sectional study of 5 – 19 year olds found a greater proportion of frequent bedwetting (>= 3 wet nights per week) in older (11 – 19 years) compared with younger children (5 – 10 years)[23]. A prospective study found that children with persistent bedwetting with accompanying daytime wetting had a higher chance of becoming adolescents with bedwetting compared with those who had bedwetting alone in childhood[24]. These findings suggest that parents of children with these patterns of bedwetting should seek early advice from a health professional. In the ALSPAC cohort, however, only 31.9% of parents of 7 ½ year-old children with bedwetting sought professional help[10]. It was previously common practice not to offer treatment for bedwetting until the child was aged at least 7 years. However, the current NICE guidelines for enuresis recommend that children over the age of 2 years, with on-going wetting problems, both day and night, who are showing appropriate toileting awareness and behaviour, should be considered for assessment and investigation to exclude a specific medical problem[25]. If initial advice and support does not lead to resolution of bedwetting, NICE recommend that first line treatment options should be offered to all children with bedwetting, particularly those whose families find the management of bedwetting burdensome and request help. Infrequent bedwetting at age five is not uncommon, but if children are still frequently wetting the bed at this age they may need professional help to become dry at night. There is evidence that treating childhood bedwetting is cost-effective compared to leaving it untreated[25]. Successful treatment can result in children having improved self-esteem[30,31] and quality of life[32].

This study adds further weight to the importance of encouraging parents to seek professional help for their child's bedwetting, rather than persisting with strategies that may

For peer review only - http://bmjopen.bmj.com/site/about/guidelines.xhtml

be ineffective. Replication of our findings in other samples would provide further reassurance of our findings. Future research is needed to determine whether there are risk factors in early childhood that predict the continuation of bedwetting at school age. Early identification of the underlying causes of a child's bedwetting, including overactive bladder, nocturnal polyuria and/or problems with sleep and arousal, could help to ensure that appropriate treatment is given.

For peer review only

For peer review only - http://bmjopen.bmj.com/site/about/guidelines.xhtml

Acknowledgments: This study is based on the Avon Longitudinal Study of Parents and Children (ALSPAC). We are extremely grateful to all the families who took part in this study, the midwives for their help in recruiting them, and the whole ALSPAC team, which includes interviewers, computer and laboratory technicians, clerical workers, research scientists, volunteers, managers, receptionists and nurses.

Collaborator: Dr Penny Dobson provided advice on the strategies used by parents to overcome bedwetting and on the current NICE guidelines.

Competing interests: We have read and understood BMJ policy on declaration of interests and declare that we have no competing interests.

Funding statement: This work was supported by the Medical Research Council grant number MR/L007231/1. The UK Medical Research Council, the Wellcome Trust (Grant reference: 102215/2/13/2) and the University of Bristol provide core support for ALSPAC.

Author contributions: Drs Joinson and Heron, Mr Grzeda, Professor Tilling and Dr Wright conceptualized and designed the study. Mr Grzeda carried out the statistical analysis and drafted the result section. Drs Heron, Joinson and Professor Tilling supervised the statistical analysis and interpreted the results. All authors were involved in drafting the manuscript and approved the final manuscript as submitted.

Data sharing statement: This is a secondary data analysis based on data from the ALSPAC cohort. The ALSPAC Executive encourages and facilitate data sharing with all 'bona fide' researchers. The access policy for the ALSPAC data can be found at http://www.bristol.ac.uk/media-library/sites/alspac/documents/ALSPAC_access_policy.pdf.

For peer review only - http://bmjopen.bmj.com/site/about/guidelines.xhtml

REFERENCES

1. Austin PF, Bauer SB, Bower W et al. The standardization of terminology of lower urinary tract function in children and adolescents: update report from the Standardization Committee of the International Children's Continence Society. *J Urol* 2014;191(6):1863-1865.e13.
2. Jansson UB, Hanson M, Sillén U, Hellström AL. Voiding pattern and acquisition of bladder control from birth to age 6 years--a longitudinal study. *J Urol* 2005;174(1):289-93.
3. Fergusson DM, Horwood LJ, Shannon FT. Factors related to the age of attainment of nocturnal bladder control: an 8-year longitudinal study. *Pediatrics* 1986;78(5):884-90.
4. Butler RJ, Golding J, Northstone K; ALSPAC Study Team. Nocturnal enuresis at 7.5 years old: prevalence and analysis of clinical signs. *BJU Int* 2005;96(3):404-10.
5. Butler R, McKenna S. Overcoming parental intolerance in childhood nocturnal enuresis: a survey of professional opinion. *BJU Int* 2002;89(3):295-7. Review.
6. Schulpen TW. The burden of nocturnal enuresis. *Acta Paediatr* 1997;86(9):981-4.
7. Berry AK. Helping children with nocturnal enuresis: the wait-and-see approach may not be in anyone's best interest. *Am J Nurs* 2006;106(8):56-63.
8. Glazener CM, Evans JH, Peto RE. Alarm interventions for nocturnal enuresis in children. *Cochrane Database Syst Rev* 2003;(2):CD002911. Review.
9. Glazener CM, Evans JH, Peto RE. Tricyclic and related drugs for nocturnal enuresis in children. *Cochrane Database Syst Rev* 2003;(3):CD002117. Review.
10. Butler RJ, Golding J, Heron J; ALSPAC Study Team. Nocturnal enuresis: a survey of parental coping strategies at 7 1/2 years. *Child Care Health Dev* 2005;31(6):659-67.

For peer review only - http://bmjopen.bmj.com/site/about/guidelines.xhtml

11. Schlomer B, Rodriguez E, Weiss D, Copp H. Parental beliefs about nocturnal enuresis causes, treatments, and the need to seek professional medical care. *J Pediatr Urol* 2013;9(6 Pt B):1043-8.

12. Caldwell PH, Nankivell G, Sureshkumar P. Simple behavioural interventions for nocturnal enuresis in children. *Cochrane Database Syst Rev* 2013;19(7):CD003637.

13. Boyd, A, Golding, J, Macleod, JAA, et al. Cohort Profile: The 'Children of the 90s'--the index offspring of the Avon Longitudinal Study of Parents and Children. *International Journal of Epidemiology* 2013;42:111-127.

14. Austin PC. An introduction to Propensity Score Methods for Reducing the Effects of Confounding in Observational Studies. *Multivariate Behavioral Research* 2011;46: 399-424.

15. Imbens GW. Nonparametric Estimation of Average Treatment Effects under Exogeneity: A Review. National Bureau of Economic Research. *The Review of Economics and Statistics* 2004, 86(1): 4–29.

16. Lunceford JK, Davidian M. Stratification and weighting via the propensity score in estimation of causal treatment effects: A comparative study. *Statistics in Medicine* 2004;23, 2937–2960.

17. Monson RR. Occupational Epidemiology, 2nd Edition, CRC Press, Boca Raton, London, New York, Washington 1990.

18. Norton EC, Miller MM, Kleinman LC. Computing adjusted risk ratios and risk differences in Stata. The Stata Journal, 2001.

19. Royston P, White IR. Multiple Imputation by Chained Equations (MICE): Implementation in Stata, Journal of Statistical Software 2011; 45(4), 1-20.

For peer review only - http://bmjopen.bmj.com/site/about/guidelines.xhtml

20. StataCorp. 2015. *Stata Statistical Software: Release 14*. College Station, TX: StataCorp LP.

21. Franco I, von Gontard A, DeGennaro M. Evaluation and treatment of nonmonosymptomatic nocturnal enuresis: A standardization document from the International Children's Continence Society. *Journal of Pediatric Urology* 2013;9: 234-243.

22. Nevéus T, von Gontard A, Hoebeke P, et al. The standardization of terminology of lower urinary tract function in children and adolescents: report from the Standardisation Committee of the International Children's Continence Society. *J Urol* 2006; 176(1): 314-24.

23. Yeung CK, Sreedhar B, Sihoe JD, et al. Differences in variables of nighttime enuresis between children and adolescents: a critical appraisal from a large epidemiological study. *BJU Int* 2006; 97(5): 1069-1073.

24. Heron J, Grzeda M, von Gontard A, Wright A, Joinson C. Trajectories of urinary incontinence in childhood and bladder and bowel symptoms in adolescence: prospective cohort study. *BMJ Open*, in press.

25. NICE (2010). Nocturnal enuresis: The management of bedwetting in children and young people. NICE Clinical Guidelines, No. 111. National Clinical Guideline Centre, 2010.

26. Christmanson L, Lisper H. Parent behaviors related to bed-wetting and toilet-training as etiological factors in primary enuresis. Scandinavian Journal of Behaviour Therapy. *Scandinavian Journal of Behaviour Therapy* 1982;52 11(1):29-37.

27. Novello AC, Novello JR. Enuresis. *Pediatric Clinics of North America* 1987;34(3):719–33.

For peer review only - http://bmjopen.bmj.com/site/about/guidelines.xhtml

28. Blackwell C. A guide to enuresis: A guide to treatment of enuresis for professionals. Bristol: ERIC, 1989.

29. Forsythe WI, Redmond A. Enuresis and spontaneous cure rate: study of 1129 enuretics. *Arch Dis Child* 1974;49, 259-263.

30. Longstaffe S, Moffatt ME, Whalen JC. Behavioral and self-concept changes after six months of enuresis treatment: a randomized, controlled trial. *Pediatrics* 2000;105(4 Pt 2):935-40.

31. Hägglöf B, Andrén O, Bergström E, Marklund L, Wendelius M. Self-esteem in children with nocturnal enuresis and urinary incontinence: improvement of self-esteem after treatment. *Eur Urol* 1998;33 Suppl 3:16-9.

32. Equit M, Hill J, Hübner A, von Gontard A. Health-related quality of life and treatment effects on children with functional incontinence, and their parents. *J Pediatr Urol* 2014;10(5):922-8.

ew only

Figure 1

Prevalence of parental strategies for overcoming bedwetting (figure 1a) and prevalence of children with parents employing a given number of strategies (figure 1b).

Figure 2

Estimated differences in the risk of bedwetting at 9 ½ years in children whose parents used each strategy compared with those who did not use the strategy: results of the propensity score-based model (figure 2a) and the logistic regression analysis (figure 2b).

For peer review only

For peer review only - http://bmjopen.bmj.com/site/about/guidelines.xhtml

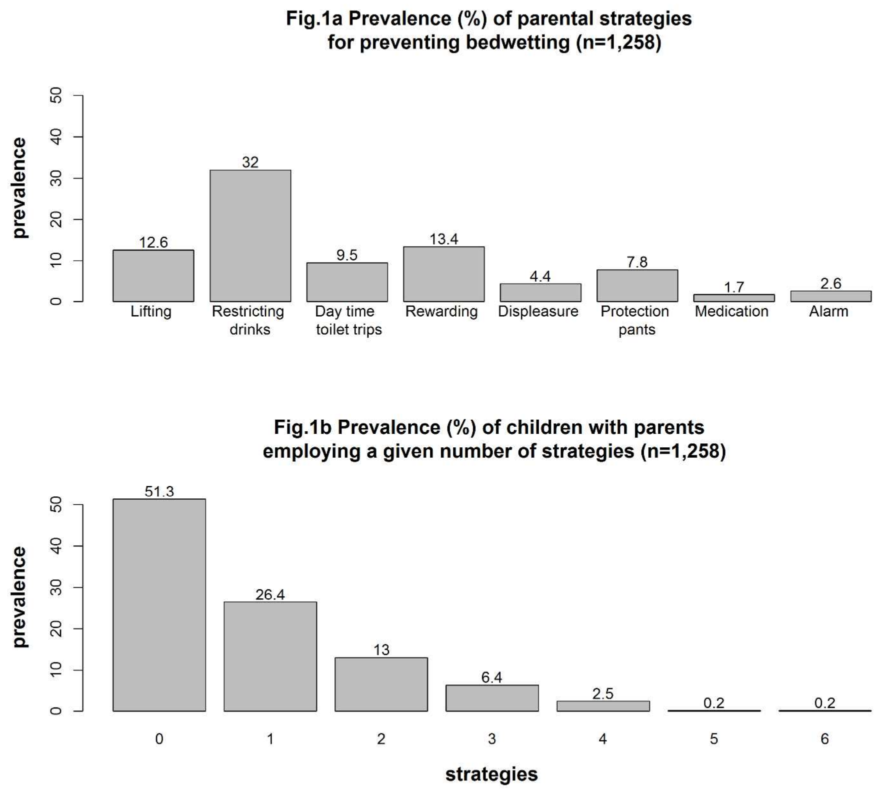

Figure 1

279x228mm (300 x 300 DPI)

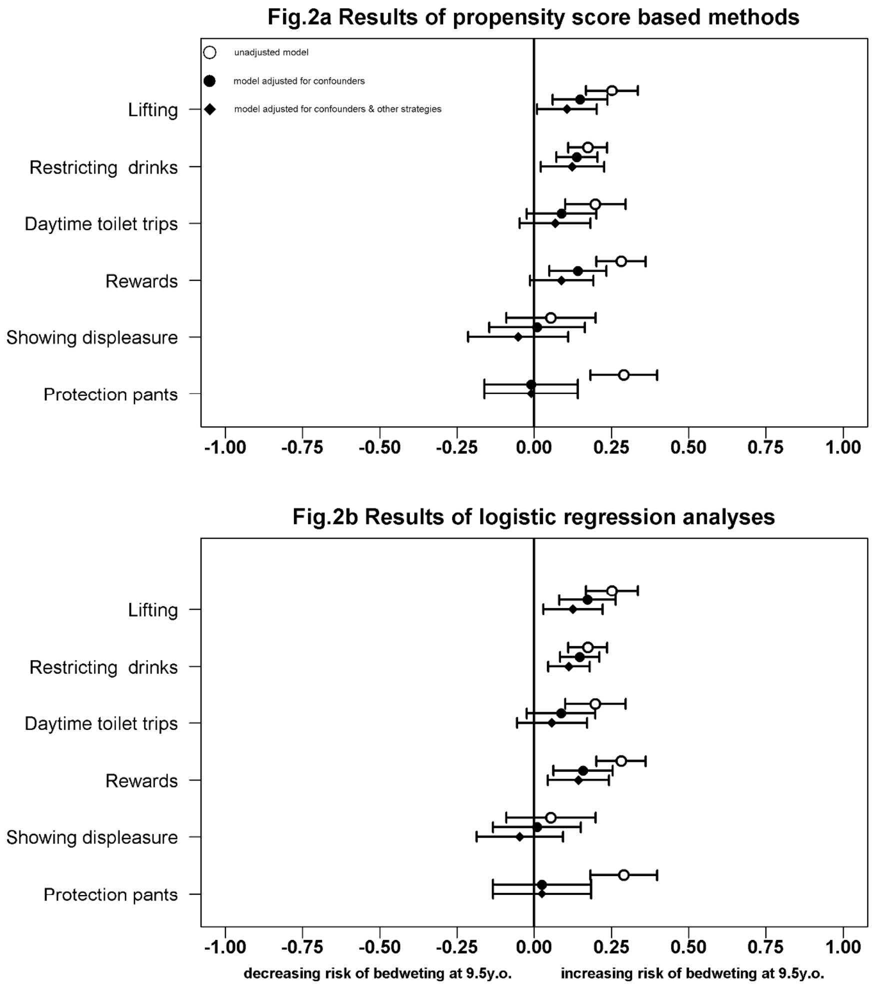

Figure 2

228x228mm (300 x 300 DPI)

Supplementary online material

Rationale for applying propensity score methods

The main purpose of propensity score-based methods is to draw causal inferences about effects of treatments from observational data. In order to do this certain assumptions are required. In observational data the process of selection to the 'treated' and 'non-treated' groups is not random. Direct comparisons of the outcomes in both groups are likely to be biased if 'treated' and 'non-treated' groups differ systematically on certain variables (both measured and unmeasured) that may also be associated with the outcome. It is, therefore, not possible to establish to what extent the observed results are effects of the 'treatment' versus other important variables that remained uncontrolled in the process of selection of cases into the groups. Dealing with this problem of confounding is a central problem of studies based on observational data. As Rosenabum (2005)[1] states the key issues in such studies are to remove 'overt biases' (related to measured/observed confounders) and to address somehow uncertainty about those hidden (unmeasured confounders).

Multivariable regression and, more recently, propensity score-based methods can be used to minimise effects of confounders when estimating treatment effects using observational data. These methods adjust the crude results to take into account the non-random mechanism of selection of cases to the 'treated' versus 'non-treated' groups by controlling for differences between both groups. Propensity score-based methods are based on the assumption that differences between 'treated' and 'non-treated' groups can be explained in terms of observable variables and bias should, therefore, be minimised by ensuring similarity of both groups on these variables. This is achieved using propensity score-based methods either by matching each case from the 'treated' group to at least one similar case from the 'non-treated' group or by re-weighting both groups in order to construct a synthetic sample in which both groups are similar with respect to the observed confounders. Similarity of the groups is assessed in terms of the probability of receiving the 'treatment' as estimated from the observed variables of the cases. This probability, called the 'propensity score', is further used to create/or reshape the sample in which distributions of variables of study participants are similar in both groups. If this condition is fulfilled then treated and untreated groups are balanced on measured confounders. However, in order to compare their results directly it is also important to assume that all of the important confounders were measured and included in the analysis. It is only under these conditions that estimates of causal effects are assumed to be unbiased.

Application of propensity score-based methods in the current study

We used propensity score-based methods to assess the effectiveness of parental strategies aimed at preventing bedwetting. Within our study, 'treated' refers to those children receiving each parental strategy at 7 ½ years and 'non-treated' refers to those not receiving the strategy. The propensity score-based analysis involves three general steps: (1) constructing the propensity scores; (2) balancing the sample based on their propensity scores; and (3) estimating effects of the treatment (parental strategy). At each of these steps in the analysis, decisions need to be made that influence the results and final conclusions. Below, we present the key decisions we made in conducting the propensity score-based analysis:

a) Average treatment effect versus average treatment effect on treated:

For peer review only - http://bmjopen.bmj.com/site/about/guidelines.xhtml

One of the most crucial decisions in the analysis concerns the indicator of the impact of the treatment. These are: average treatment effect (ATE) or average treatment effect on treated (ATT). They are both rooted within the counterfactual framework, which assumes that each participant included in the study has two potential outcomes i.e. the outcome when treatment was applied and the outcome when treatment was not applied. In reality, only one of these outcomes is truly observed, and the other is unobserved (counterfactual). The ATE is defined as the difference between averages of these two potential outcomes at the population level. The ATT is the difference between potential outcomes calculated for the treated group.
The aim of the analysis was to estimate the effect of each parental strategy aimed at preventing bedwetting at 7½ years as a difference in risk of bedwetting at 9½ years between those children receiving the strategy (treated) versus those children not receiving the strategy (non-treated). We assessed the effectiveness of each parental strategy by means of the average treatment effect for the treated (ATT) because we were interested in the average effect of treatment on those children who ultimately received the treatment rather than in the estimation of the average treatment effect at the population level (ATE).

b) Selection of propensity score based method:
The four most reported methods for using propensity scores in the estimation of the effect of treatment are propensity score matching, stratification on propensity score, inverse probability weighting and covariate adjustment using propensity scores. Austin (2011)[2] provides a detailed discussion of the strengths and limitations of these methods. In brief, the method based on inverse probability weighting is favoured, whilst the other methods were found to be more prone to bias in Monte Carlo studies. We used the inverse probability of treatment weighting (IPTW) where the synthetic balanced sample is constructed (participant variables are independent on the assignment to treated/non-treated groups). We estimated the propensity scores by logistic regression.

c) *Selection of baseline variables to the propensity score model*:
There are at least four possible strategies for variable selection and there is lack of consensus in the literature on which strategy is the most appropriate.[2] The available strategies are: (1) propensity model should include all measured, theoretically relevant variables; (2) model should include variables that are associated with the treatment; (3) model should include variables that are associated with the outcome; (4) model should include only those variables that are associated both with treatment and outcome. The final strategy seems to be the favoured approach as regards theoretical arguments and results of simulation studies.[2] Therefore, we chose this model including only those variables that are associated both with treatment (parental strategy) and outcome (bedwetting at 9 ½ years). We used a two-stage procedure to derive the list of baseline variables to include in the model. Firstly, we conducted two logistic regression analyses in the first completed dataset (m=1). In the first regression model, the outcome variable was predicted from all theoretically relevant variables relating to children and their family (see table S1 for the list of all variables included). In the second regression model, each parental strategy was the outcome variable. Secondly, we applied a threshold for selection of variables to the propensity score model - only the variables that had associations (expressed in terms of odds ratios) with the outcome variables in both models of <0.90 or >1.20.[3] We enriched the propensity score models by including the parental strategies that were highly correlated with the

For peer review only - http://bmjopen.bmj.com/site/about/guidelines.xhtml

specific strategy considered in each model. The alternative strategy was included in the model for a given strategy if the tetrachoric correlation was >0.45.

d) *Diagnostics:*
Propensity score based diagnostic methods are designed to examine whether the distributions of the baseline variables are the same/or similar enough in treated and non-treated groups. There are several specific diagnostic procedures that are recommended. Among them the most frequently used are those examining the standardized differences between means of baseline variables after matching/weighting and those comparing their variances. Before analysing the treatment effects, we assessed the adequacy of the propensity score model by checking the overlap in propensity scores. We based our diagnostic checks on the examination of differences between means of baseline variables in the weighted sample. Categorical variables with more than two categories were examined using sets of binary variables. Following the literature we assumed that absolute standardized mean difference < 0.1 indicates negligible differences between groups. Since the model computations were conducted on 50 datasets obtained from the multiple imputation procedure (see section below for details) we performed the diagnostic checks in 5 randomly selected datasets (m=1,4,5,37,50). See tables S2 to S7 for the results of the diagnostic checks to assess the adequacy of the propensity score models.

e) Dealing with missing data and estimating the treatment effects:
The magnitude of missing data per variable is presented in table S1. To deal with the missing data, we used multiple imputation by chained equations within ICE (Imputation by Chained Equations) STATA package v.14. We generated 50 imputed datasets using 10 cycles of regression switching. We used a range of auxiliary data, which does not itself form part of the analytical model. The criteria of variable selection to the imputation model for each variable included: (i) theoretical and empirical evidence from prior published research; (ii) data driven: variable was selected if in the current dataset it was strongly associated with observed values of the imputed variable or missingness on that variable; (iii) additionally, the imputation models for every variable included the parental strategies and bedwetting at 9½ years.
Conducting the analysis based on propensity scores on multiply imputed datasets required a decision about the exact routine by which the treatment effects are calculated. There are two possibilities: (1) first estimate the propensity score in each of 50 completed datasets and average the propensity score for every subject across all datasets and then estimate treatment effects based on those aggregated propensity scores; (2) estimate propensity scores and treatment effects separately in every completed dataset and then aggregate the treatment effects obtained according to Rubin's rules. Published simulation studies suggest that first strategy provides biased results[4] therefore, we used the second strategy.
To our best knowledge there is no STATA package that deals with treatment effects in the context of multiple imputation, so we developed our own syntax using loops to estimate treatment effects and standard errors separately within each file and exported the results to Excel. At the next stage, we aggregated the exported results by implementing Rubin's rules for estimating the effects and standard errors from the multiply imputed datasets. [5] For the estimation *teffects ipw* we used the STATA built-in command and for the diagnostic procedures we used *tebalance summarize*. Models for medication and bedwetting alarm were not possible to estimate due to a very low number of children receiving these interventions.

For peer review only - http://bmjopen.bmj.com/site/about/guidelines.xhtml

We conducted diagnostics checking the plausibility of the imputation process by means of the *midiagplots* STATA command after datasets from the *ICE* package were successfully exported. Due to the large number of generated datasets, the diagnostics were based only on a subset of five randomly selected datasets (m=1,4,5,37,50) obtained from the MI procedure. The diagnostics were conducted with the assumption that the missing data mechanism was MAR. We compared the distributions of observed, imputed and completed values of each variable. The diagnostics revealed that they did not differ greatly which suggested that the imputation model was correctly specified. (The figures and tables including those diagnostic statistics are available on request).

<u>Caveats of using propensity score based methods</u>

Propensity score based methods are a group of methods used to estimate treatment effects from observational data by balancing treatment and non-treatment groups on the set of measured confounders (**X**). As Rosenbaum and Rubin (1983)[6] showed that this can be achieved by using propensity scores instead of all combinations of variables from **X**.

All propensity score methods rely on several assumptions of which the most crucial is the one known as the conditional independence assumption (CIA). It consists of two parts: (a) the potential result of being treated (Y_1) and the potential result of remaining untreated (Y_0) are independent of the treatment group (G), after controlling for a vector of measured confounders **(X):** $Y_{\mathbf{0},} Y_1 \perp G|\boldsymbol{X}$ and (b) every case, (with any characteristic on vector **X**) has a positive chance of being included in either treatment group, which is $0<P(G=1|\mathbf{X})<1$.

Part (a) of CIA assumes that there are no unmeasured confounders. If vector **X** does not include all important confounders (those related both to the outcome and the treatment) (a) is not satisfied and causal inferences might be biased. Unfortunately, this is the untestable part of CIA and needs to be assumed. Part (b) of CIA implies that there must be an overlap between treated and non-treated groups with regards to their characteristics on vector **X** which means that any person with any characteristic on **X** within treatment group will have their counterpart in non treatment group[1]. In contrary to (a), part (b) of CIA concerning the overlap of treated and non-treated is testable and can be made by comparing distributions of propensity scores for both groups. We have done this by graphical examination of propensity scores estimated for both treatment groups (see figures S2-S7). This assumption was fulfilled to a satisfactory extent since the differences we observed in the distributions of propensity scores in treated and non-treated groups were minor.

It should be also added here that since in the current paper we relied on ATT, both parts of CIA are adopted in their weaker, less stringent, versions, This, weaker version of CIA requires for (a) only $Y_{\mathbf{0},} \perp G|\boldsymbol{X}$ to hold (which remains untestable) and for (b) that cases from the treatment group will have their counterparts in the untreated group (but the overlap in the opposite direction is not necessary now). As shown in figures S2-S7 this milder version of the assumption generally held in the data.

After applying methods using propensity scores the assessment of balance achieved between both compared groups is the final diagnostic step. It is also notable that the assessment of balance obtained as a result of applying inverse propensity score weighting returned satisfactory results.

For peer review only - http://bmjopen.bmj.com/site/about/guidelines.xhtml

References

1. Rosenbaum PR. Observational Study. In: Everitt BS, Howell DC, eds. Encyclopedia of Statistics in Behavioural Science Volume 3. Chichester: John Wiley & Sons, Ltd. 2005: 1451-1462.
2. Austin PC. An introduction to propensity score methods for reducing the effects of confounding in observational studies. Multivariate *Behav Res* 2011; 46(3): 399–424.
3. Monson RR. Occupational Epidemiology. 2nd Edition. CRC Press, Boca Raton, London, New York, Washington, DC, 1990.
4. Mitra R, Reiter JP. A comparison of two methods of estimating propensity scores after multiple imputation. *Stat Methods Med Res* 2016; 25(1): 188-204.
5. White IR, Royston P, Wood AM. Multiple imputation using chained equations: Issues and guidance for practice. *Statistics in Medicine* 2011; 30(4): 377-399.
6. Rosenbaum PR, Rubin DB, The central role of the propensity score in observational studies for causal effects. *Biometrika* 1983; 70: 41-55.

For peer review only - http://bmjopen.bmj.com/site/about/guidelines.xhtml

Table S1. Model variables for multiple imputation (MI) with numbers of missing cases per variable (n =1258).

	MODEL VARIABLES	Number of missing/ imputed cases by MI	Imputation model
	Confounders		
Variable number:	Child and family variables:		
1	Gender	0	none
2	Developmental level at 18 months (m)[1]	100	linear regression
3	Temperament - Activity at 24 m[2]	129	logistic
4	Temperament - Adaptability at 24 m[2]	135	logistic
5	Temperament - Intensity at 24 m[2]	170	logistic
6	Temperament - Mood at 24 m[2]	132	logistic
7	Temperament - Persistence at 24 m[2]	140	logistic
8	Prosocial behaviour score at 81m[3]	146	logistic
9	Hyperactivity score at 81m[3]	146	logistic
10	Emotional symptoms score at 81m[3]	145	logistic
11	Conduct problems score at 81 m[3]	144	logistic
12	Peer problems score at 81 m[3]	144	logistic
13	Total behavioral problems score at 81 m[3]	146	logistic
14	EAS temperament -Emotionality 69 m[4]	157	logistic
15	EAS temperament -Activity 69 m[4]	149	logistic
16	EAS temperament -Shyness 69 m[4]	149	logistic
17	EAS temperament -Sociability 69 m[4]	179	logistic
18	Toilet training has not been started by 24 m (parental report)	562	logistic

For peer review only - http://bmjopen.bmj.com/site/about/guidelines.xhtml

19	Verbal IQ 8 years (y)[5]	296	logistic
20	Performance IQ 8 y[5]	297	logistic
21	Full scale IQ 8 y[5]	303	logistic
22	Social communication 7.5 y[6]	21	logistic
23	Stressful life events (child related) 42 m[7]	137	logistic
24	Sleep problems at 42 m (parental report)	83	logistic
25	Child's general health at 81 m (parental report)	214	logistic
	<u>Problems with urinating at 7.5 y</u> (parental report)		
26	Frequency child shows signs of needing to urinate	<5	logistic
27	Going to toilet without reminder	<5	logistic
28	Child needs to dash to toilet to urinate	13	logistic
29	Bedwetting frequency (>= twice a week)	0	none
30	Daytime wetting	<5	logistic
	<u>Problems with soiling at 7.5 y</u> (parental report)		
31	Daytime soiling	<5	logistic
32	Nighttime soiling	7	logistic
	<u>Socioeconomic indicators</u> (parental report)		
33	Family size (3 children or more)	22	logistic
34	Crowding index at 85 m	143	logistic
35	Home ownership at 85 m	146	logistic
36	Difficulty affording elementary goods for mother at 85 m	141	logistic
37	Difficulty affording elementary goods for child at 85 m	186	logistic
38	No car at 33m	126	logistic
39	Social class at 3 y11 m - based on mother's occupation	659	logistic
40	Social class at 3 y11 m - based on father/partner's occupation	340	logistic

For peer review only

	Mother: (maternal report)		
42	Maternal education (reported in antenatal period)	32	logistic
43	Early parenthood	0	
44	Marital status of mother	139	logistic
45	Mother has paid work outside home since child born (85 m)	124	logistic
46	Mother has help looking after child (85 m)	168	logistic
47	Mother has help with housework (85 m)	165	logistic
48	Mother feels she is getting enough sleep (85 m)	144	logistic
49	Mother spends enough time with child (85 m)	158	logistic
50	Mother had stressful life events since child's 5th birthday	128	logistic
51	Mother's general health (73 m)	132	logistic
52	Maternal psychopathology – depression (73 m)	135	logistic
53	Maternal psychopathology - anxiety at (73 m)	142	logistic
54	Mother bedwetting history	41	logistic
60	Mother daytime wetting history	41	logistic
61	Parenting style nurturing	113	logistic
62	Parenting style discipline	95	logistic
63	Toilet training at 15 m[8]	73	logistic
64	Toilet training at 24 m[8]	109	logistic
65	Rules about smoking in house	141	logistic
66	Does anyone smoke in the house?	164	logistic
65	Father figure is natural father of child	154	logistic
	PSM - outcome variable:		
O1	Bedwetting at 9.5 y	213	logistic
	PSM - treatment (strategy) variables:		
S1	Lifting	0	
S2	Restricting drinks	0	

For peer review only - http://bmjopen.bmj.com/site/about/guidelines.xhtml

S3	Daytime toilet trips	0
S4	Rewarding	0
S5	Medication	0
S6	Alarm prevention	0
S7	Showing displeasure	0
S8	Protection pants	0

1. Maternal rating of developmental level was assessed at 18 months using a questionnaire developed by ALSPAC including items from the Denver Developmental Screening Test (Frankenburg et al. Pediatrics 1992; 89(1):91-97) and comprising four domains of development (Cronbach's alphas: fine motor=0.679; gross motor= 0.689; communication= 0.752 and social skills= 0.624). Scores on each domain were adjusted for age in weeks, standardized (using a linear regression model and extracting the residuals) and reversed where appropriate so that high values on all scores reflected a lower level of development. We adjusted for a total development score derived from the sum of the scores on each domain.
2. Mothers completed the Toddler Temperament Scale (TTS: Fullard, et al. Journal of Pediatric Psychology 1984; 9:205-216) when the study children were 24 months. The TTS comprises statements describing specific behaviours and mothers were asked to rate how often their child behaves in that way on a scale ranging from 1 (almost never) to 6 (almost always). The scale comprises nine temperament traits, but we restricted our analysis to five traits we found were associated with bedwetting in an earlier study (Joinson et al. 2009. Longitudinal and Life Course Studies 1:73-94): *activity* (a = 0.593), *adaptability* (a = 0.637), *intensity* (a = 0.570), *mood* (a = 0.672) and *persistence* (a = 0.711).
3. Psychological problems were assessed when the child was aged 81 months using the Revised Rutter Parent Scale for Preschool Children, which is an extension of the Rutter behaviour scale (Elander & Rutter. Journal of Methods in Psychiatric Research 1996; 6:63–78). The questionnaire comprises 43 statements describing behaviours and mothers are asked to rate the extent to which each statement describes their child on a scale comprising the options 1 (certainly true), 2 (sometimes true) and 3 (not true). Responses were aggregated to create scores in five domains: *emotional difficulties*, *conduct difficulties*, *hyperactivity* and *prosocial behaviour*. High levels of problems are indicated by high scores on emotional difficulties, conduct difficulties and hyperactivity and low scores on the prosocial behaviour scale.
4. The Emotionality Activity Sociability (EAS) Questionnaire (Buss & Plomin. Temperament: Early developing personality traits. Hillsdale, NJ: Erlbaum. 1984) was administered when study children were 69 months. The questionnaire comprises 20 statements about behaviours and mothers rated the extent to which each statement describes their child on a scale ranging from 1 (not at all like) to 5 (exactly like). Scores on these items are combined to form four subscales (each comprising five items): *emotionality*, *activity level*, *shyness* and *sociability*.
5. IQ: Wechsler D. Manual for the Wechsler Intelligence Scale for Children. 3rd ed. San Antonio, TX: Psychological

For peer review only - http://bmjopen.bmj.com/site/about/guidelines.xhtml

Corporation; 1991.

6. When children were 7.5 years mothers completed the 12-item Social Communication Disorder Checklist (Skuse et al. Br J Psychiatry 2005;187:568-572). A score of 2 SD or more below the mean was selected as indicating impaired social communication.
7. Stressful life events were measured using a maternally reported questionnaire comprising 42 life events that was derived for ALSPAC using previous inventories as a basis for item selection (Barnet et al. J Psychosom Res 1983: 27, 313–320).
8. Toilet training: Mothers were asked: What do you think about toilet training for your child? Options were: "It is too early to start any toilet training yet", "I have just started toilet training" and "I have been toilet training for some time".

For peer review only

Assessing the adequacy of the propensity score models

Table S2 (i). Diagnostics summary for **LIFTING**

N in unweighted dataset: treated = 159; untreated 1,099	Model adjusted for child and family variables					Model adjusted for child and family variables and other parental strategies				
	m=1	m=4	m=5	m=37	m=50	m=1	m=4	m=5	m=37	m=50
N of treated in weighted dataset:	630	628	629	629	629	632	635	632	637	639
N of untreated in weighted dataset:	628	630	629	629	629	626	623	627	621	619

Figure S2. Overlap for models for **LIFTING** including child and family variables

m=1	m=4	m=5	m=37	m=50
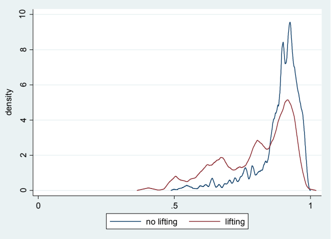	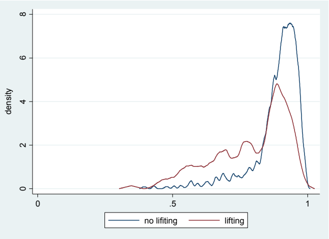	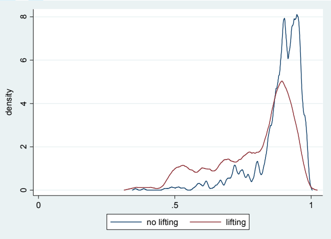	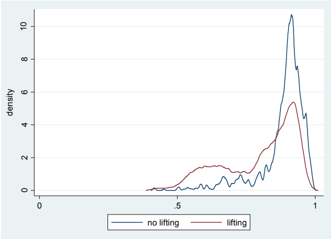	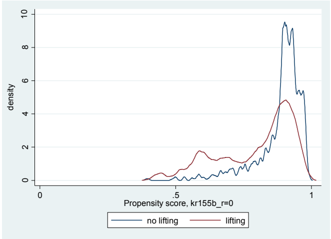
Overlap for models for **LIFTING** including child and family variables & other related parental strategies				
m=1	m=4	m=5	m=37	m=50
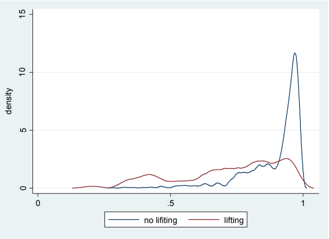	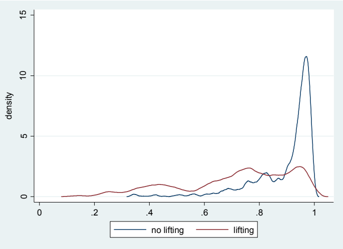	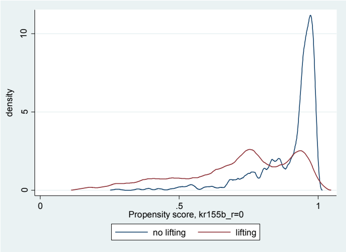	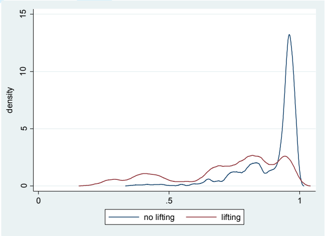	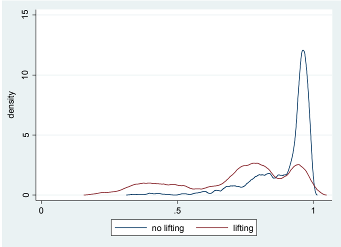

For peer review only - http://bmjopen.bmj.com/site/about/guidelines.xhtml

Table S2 (ii). Standardized differences on confounders after weighting

CONFOUNDERS	Standardized differences in weighted sample in model adjusted for child and family variables					Standardized differences in weighted sample in model adjusted for child and family variables and other parental strategies				
**Imputed dataset:**	**m=1**	**m=4**	**m=5**	**m=37**	**m=50**	**m=1**	**m=4**	**m=5**	**m=37**	**m=50**
**Child and family variables included as confounders:**										
Temperament - Activity at 24 m										
1. high score >28	.035	.026	.012	.003	.017	.054	.039	.033	.035	.043
Temperament - Adaptability at 24 m										
1. high score >17	-.002	-.009	.006	-.016	.004	.033	.014	.039	.039	.038
Temperament - Mood at 24 m										
1. high score >24	.019	-.002	.018	.017	.011	.073	.071	.063	.064	.048
Conduct problems score at 81 m										
1 abnormal (score >=4 (hyperproblems))	-.011	-.021	-.017	-.032	-.016	-.023	.003	.014	-.016	-.015
Total behavioural problems score at 81 m										
1 abnormal (score >=14 (hyper behavioral problems))	.009	-.004	-.008	.001	.007	.001	.007	.017	.002	.013
EAS temperament - activity at 69 m										
1 abnormal (score >=24 (hyper active))	-.011	.002	-.021	-.011	-.006	.041	.031	.033	.035	.036
EAS temperament -sociability at 69 m										
1 abnormal (score >=22 (antisocial))	.012	.012	.005	.013	.004	.012	.001	-.008	-.002	-.003
Verbal IQ										
1. 80<IQ<=120	.001	.022	-.001	.008	.013	-.026	-.027	-.028	.001	-.014
2. IQ>120	-.006	-.008	-.008	-.003	-.012	.015	.048	.018	-.001	.003
Performance IQ										
1. 80<IQ<=120	-.002	.018	-.001	.009	-.009	.024	.039	.029	.027	.016
2. IQ>120	-.003	-.002	-.001	-.007	-.006	-.028	-.019	-.027	-.057	-.043
Full scale IQ										
1. 80<IQ<=120	.001	.029	.022	.024	.020	-.008	.026	.028	.045	.021

For peer review only - http://bmjopen.bmj.com/site/about/guidelines.xhtml

2. IQ>120	.003	-.006	-.016	-.010	-.012	.013	.009	-.026	-.037	-.037
Social cognition										
1.(score>=8)	-.011	-.038	-.033	-.027	-.007	.000	-.036	-.004	.011	.014
Going to toilet without reminder										
1. never	-.006	.000	-.001	-.005	-.012	-.013	.001	.005	-.004	-.011
Bedtime wetting severity										
1. severe	.006	-.001	.007	.000	.017	.043	.041	.051	.061	.085
Daytime soiling										
1. yes	-.024	-.041	-.044	-.052	-.037	-.001	-.029	-.013	-.006	.003
Family size (3 or more children)										
1 # of children >=3	-.013	.005	-.002	.006	-.002	-.082	-.030	-.034	-.031	-.017
Social class - based on mother's occupation										
1. Managerial and technical	.004	-.007	.012	-.009	-.031	.025	.009	-.015	.011	-.018
2. Skilled non-manual	-.010	.004	.010	.011	-.002	-.026	-.024	-.014	.002	-.018
3. Skilled manual	-.019	-.024	-.003	-.029	.010	-.024	-.028	.000	.005	-.020
4. Partly skilled	-.003	-.005	-.022	.003	.019	.024	.037	.036	-.006	.044
5. Unskilled	.017	.019	.020	.014	.021	.037	.013	.029	.014	.036
Social class - based on father/partner's occupation										
1. Managerial and technical	.001	-.003	-.017	.002	-.005	.009	-.003	-.026	.013	.016
2. Skilled non-manual	.000	.012	.014	-.013	-.009	.002	-.002	-.002	-.020	-.043
3. Skilled manual	.003	.019	.024	.021	.030	.009	.023	.054	.0358	.059
4. Partly skilled	-.020	-.032	-.027	-.029	.002	-.044	-.020	-.053	-.044	-.017
5. Unskilled	.010	-.004	.007	.016	-.037	.015	.000	.034	.0199	-.016
Early parenthood										
1. Had child at age <19	.007	.005	.002	-.010	.000	.002	.001	.000	-.021	-.004
Amount of help mother has in looking after child at 85 m										
1. Too little	-.007	.002	.013	-.023	-.015	.030	.008	.023	-.001	.015
Mother state of health 73 m										
1. Mostly well & healthy	.030	.020	.032	.018	.025	.042	-.012	.029	.019	.034

For peer review only - http://bmjopen.bmj.com/site/about/guidelines.xhtml

2. Often unwell & hardly ever well	-.022	-.024	-.042	-.023	-.041	-.014	.037	-.034	.0159	-.011
Mother's history of bedwetting										
1. Mother had a problem when older than 5yo	-.009	-.003	-.018	-.011	-.018	-.034	-.020	-.028	-.014	-.023
Toilet training when child is 15 m										
1. Just started toilet training	.023	.002	-.005	.012	.009	.040	.022	.039	.031	.040
2. Toilet training for some time	.017	.016	.019	.015	.016	.051	.021	.019	.026	.018
Rules about smoking in household										
1. Smoking allowed in some rooms	-.021	-.017	-.017	-.010	-.014	-.005	-.014	-.014	.0196	.009
2. Smoking allowed	.004	.013	.014	.011	.010	-.002	.001	.009	.000	-.006
Father figure is natural father of child										
1. No / not living with father figure	-.005	-.003	.008	.021	.024	-.026	-.006	.011	.017	.021
**Related parental strategies variables (those with correlations>.45):**										
Restricting drinks										
1. yes						.000	.015	-.004	.021	.026

Table S3 (i). Diagnostics for **RESTRICTING DRINKS**

**N in unweighted dataset: treated = 403; untreated 855**	**Model adjusted for child and family variables**					**Model adjusted for child and family variables and other parental strategies**				
	**m=1**	**m=4**	**m=5**	**m=37**	**m=50**	**m=1**	**m=4**	**m=5**	**m=37**	**m=50**
**N of treated in weighted dataset:**	**631**	**631**	**633**	**631**	**633**	**627**	**630**	**630**	**629**	**629**
**N of untreated in weighted dataset:**	**628**	**627**	**625**	**627**	**625**	**631**	**628**	**628**	**630**	**630**

Figure S3. Overlap for models for **RESTRICTING DRINKS** including child and family variables

m=1	m=4	m=5	m=37	m=50
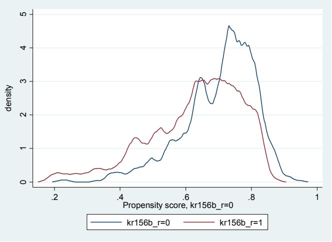	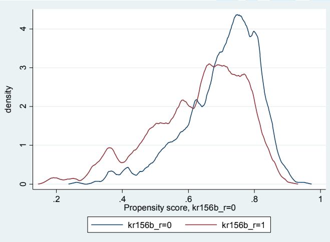	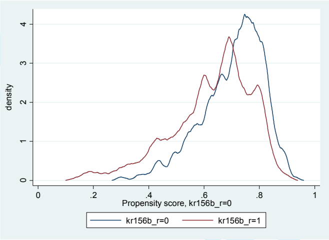	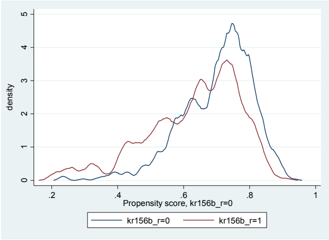	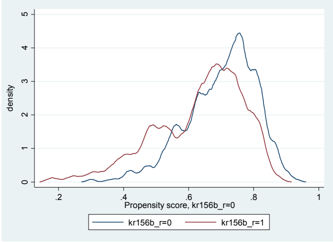
Overlap for models for **RESTRICTING DRINKS** including child and family variables & other related parental strategies				
m=1	m=4	m=5	m=37	m=50
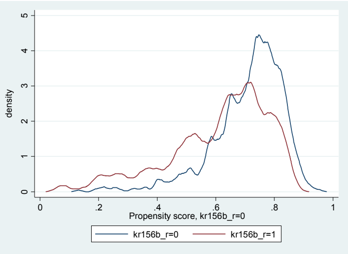	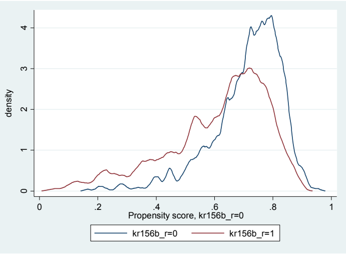	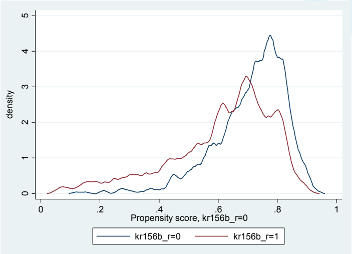	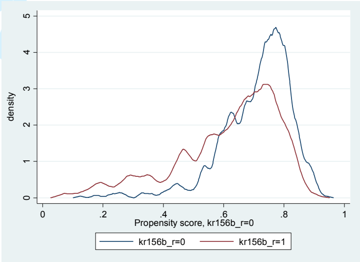	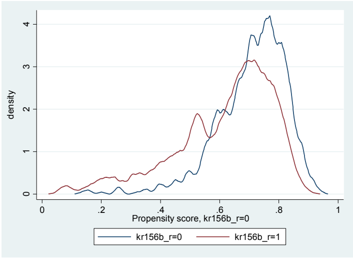

For peer review only - http://bmjopen.bmj.com/site/about/guidelines.xhtml

Table S3 (ii). Standardized differences on confounders after weighting

CONFOUNDERS	Standardized differences in weighted sample in model adjusted for child and family variables					Standardized differences in weighted sample in model adjusted for child and family variables and other parental strategies				
**Imputed dataset:**	**m=1**	**m=4**	**m=5**	**m=37**	**m=50**	**m=1**	**m=4**	**m=5**	**m=37**	**m=50**
**Child and family variables included as confounders:**										
Gender										
1. Female	*.017*	*.001*	*.010*	*-.004*	*.022*	*.025*	*.016*	*.003*	*.004*	*.027*
Temperament - Adaptability at 24 m										
1. High score >17	*.011*	*.036*	*.036*	*.009*	*.017*	*.020*	*.025*	*.041*	*.014*	*.010*
Emotional symptoms score at 81 m										
1. High score >=4	*-.007*	*.002*	*-.002*	*-.016*	*.006*	*-.108*	*.008*	*-.009*	*-.011*	*.011*
Conduct problems score at 81 m										
1. High score >=4	*.031*	*.041*	*.056*	*.016*	*.057*	*.042*	*.067*	*.068*	*.031*	*.075*
Total behavioral problems at 81 m										
1. High score >=14	*.014*	*.019*	*.031*	*.012*	*.027*	*.028*	*.038*	*.046*	*.030*	*.046*
EAS temperament -sociability at 69 m										
1 abnormal (score >=22 (antisocial))	*.029*	*.039*	*.034*	*.017*	*.020*	*.036*	*.056*	*.037*	*.031*	*.046*
Verbal IQ at 8 y										
1. 80<IQ<=120	*.019*	*.003*	*-.013*	*-.012*	*.015*	*-.001*	*-.014*	*-.023*	*-.024*	*-.012*
2. IQ>120	*-.014*	*-.014*	*-.001*	*.002*	*-.030*	*-.002*	*-.006*	*.002*	*.008*	*-.007*
Performance IQ at 8 y										
1. 80<IQ<=120	*-.022*	*.003*	*.000*	*.027*	*.009*	*-.045*	*-.019*	*-.019*	*-.003*	*-.013*
2. IQ>120	*-.005*	*-.017*	*-.011*	*.008*	*-.009*	*.018*	*-.005*	*.012*	*.033*	*.024*
Full scale IQ at 8 y										
1. 80<IQ<=120	*.015*	*.002*	*.016*	*.011*	*.018*	*.005*	*-.013*	*.019*	*-.002*	*-.015*
2. IQ>120	*-.012*	*-.014*	*-.020*	*.006*	*-.020*	*.012*	*.009*	*-.012*	*.032*	*.014*
Sleep problems at 42 m										
1 (6 sleep difficulties or more)	*-.027*	*-.001*	*-.015*	*-.039*	*-.003*	*-.039*	*-.001*	*-.026*	*-.049*	*-.010*
Child's general health at 81 m										

For peer review only - http://bmjopen.bmj.com/site/about/guidelines.xhtml

1. minor problems	*.012*	*-.007*	*-.007*	*.003*	*.017*	*.046*	*.021*	*.017*	*.028*	*.038*
2. ill/unwell	*-.025*	*-.009*	*-.020*	*-.010*	*-.037*	*-.011*	*-.005*	*-.001*	*.007*	*-.010*
Child goes to toilet without reminder at 7.5 y										
1. never	*-.002*	*.008*	*.002*	*-.006*	*.000*	*-.041*	*-.016*	*-.011*	*-.021*	*-.018*
Child needs to dash to toilet to urinate at 7.5 y										
1. straight away	*.014*	*.021*	*.042*	*.001*	*.007*	*-.052*	*-.028*	*-.008*	*-.056*	*-.042*
Daytime wetting at 7.5 y										
1.yes	*.043*	*.031*	*.055*	*.022*	*.027*	*.038*	*.029*	*.033*	*.008*	*.010*
Social class - based on mother's occupation										
1. managerial and technical	*.003*	*-.004*	*.005*	*.003*	*-.021*	*.007*	*-.012*	*-.005*	*-.017*	*-.032*
2. Skilled non-manual	*-.021*	*-.017*	*-.025*	*-.033*	*-.010*	*-.039*	*-.038*	*-.027*	*-.039*	*-.017*
3. Skilled manual	*.005*	*.014*	*.030*	*-.029*	*.002*	*.012*	*.019*	*.045*	*-.019*	*.013*
4. Partly skilled	*.021*	*.026*	*-.001*	*.030*	*.020*	*.013*	*.040*	*.012*	*.052*	*.052*
5. Unskilled	*-.001*	*-.014*	*.023*	*.041*	*.024*	*.024*	*.008*	*.007*	*.050*	*-.004*
Social class - based on father/partner's occupation										
1. Managerial and technical	*-.003*	*-.014*	*-.011*	*-.013*	*-.014*	*.007*	*-.022*	*.010*	*-.003*	*-.020*
2. Skilled non-manual	*-.001*	*-.001*	*-.001*	*-.008*	*-.004*	*.027*	*.021*	*.014*	*.022*	*.027*
3. Skilled manual	*-.013*	*-.012*	*-.015*	*-.003*	*-.006*	*-.066*	*-.042*	*-.042*	*-.050*	*-.028*
4. Partly skilled	*.000*	*.021*	*.024*	*.011*	*.013*	*.020*	*.036*	*.013*	*.024*	*.011*
5. Unskilled	*.026*	*-.006*	*.015*	*.014*	*.011*	*.033*	*.004*	*.029*	*.016*	*.016*
Early parenthood										
1.Having child at age <19	*-.025*	*.004*	*.008*	*-.010*	*.016*	*.000*	*.022*	*.029*	*.011*	*.039*
Amount of help mother has looking after child at 85m										
1. Too little	*-.010*	*.001*	*.021*	*.009*	*.015*	*.005*	*.014*	*.037*	*.026*	*.031*
Mother's bedwetting history										
1. Mother had bedwetting after 5 y	*-.009*	*-.014*	*.008*	*-.012*	*-.030*	*-.020*	*-.030*	*-.030*	*-.033*	*-.081*
Mother daytime wetting history										

For peer review only - http://bmjopen.bmj.com/site/about/guidelines.xhtml

1. Mother had daytime wetting after 5 y	*.000*	*.008*	*.025*	*.002*	*.025*	*.010*	*.014*	*.029*	*.015*	*.031*
Toilet training child at 15 m										
1. Just started	*-.011*	*.001*	*.007*	*.011*	*.012*	*-.018*	*-.020*	*-.029*	*-.011*	*-.017*
2. For some time	*-.006*	*-.002*	*.005*	*-.007*	*.004*	*.023*	*.015*	*.023*	*.012*	*.026*
Rules about smoking in household										
1. Smoking allowed in some rooms	*.012*	*-.006*	*.002*	*.008*	*.009*	*.021*	*.011*	*.024*	*.026*	*.032*
2. Smoking allowed	*.027*	*.024*	*.033*	*.033*	*.002*	*.018*	*.014*	*.021*	*.006*	*-.008*
Father figure is natural father of child										
1. No / not living with father figure	*.029*	*.015*	*.037*	*.046*	*.049*	*.033*	*.024*	*.038*	*.045*	*.028*
**Related parental strategies variables (with tetrachoric correlations > .45):**										
Showing displeasure										
1. Yes						*-.031*	*-.012*	*-.027*	*-.029*	*-.043*

For peer review only

For peer review only - http://bmjopen.bmj.com/site/about/guidelines.xhtml

Table S4 (i). Diagnostics for **DAYTIME TOILET TRIPS**

**N in unweighted dataset: treated = 119; untreated 1,139**	**Model adjusted for child and family variables**					**Model adjusted for child and family variables and other parental strategies**				
	**m=1**	**m=4**	**m=5**	**m=37**	**m=50**	**m=1**	**m=4**	**m=5**	**m=37**	**m=50**
**N of treated in weighted dataset:**	**636**	**630**	**645**	**637**	**639**	**651**	**659**	**659**	**649**	**657**
**N of untreated in weighted dataset:**	**622**	**628**	**613**	**621**	**619**	**607**	**599**	**599**	**609**	**601**

Figure S4. Overlap for models for **DAYTIME TOILET TRIPS** including child and family variables

m=1

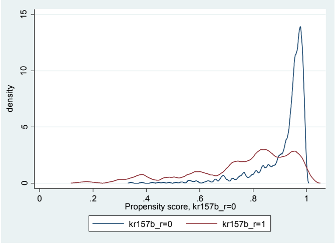

m=4

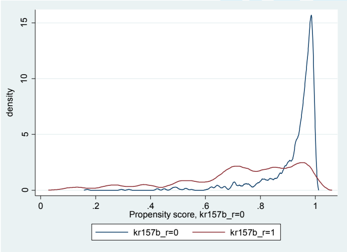

m=5

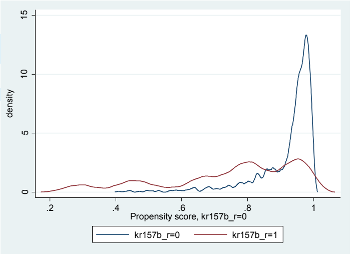

m=37

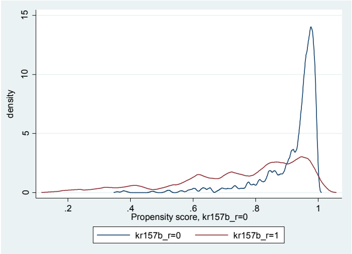

m=50

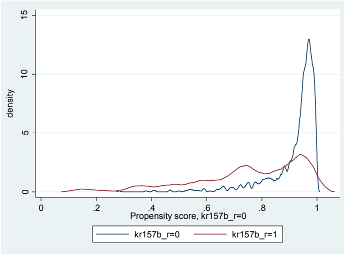

Overlap for models for **DAYTIME TOILET TRIPS** including child and family variables & other related parental strategies

m=1

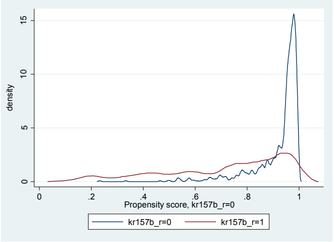

m=4

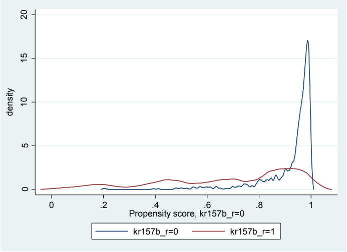

m=5

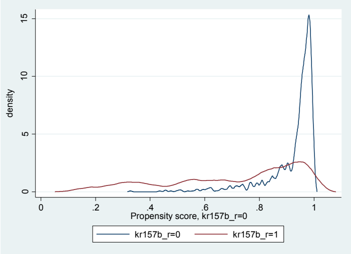

m=37

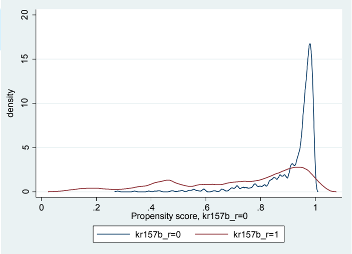

m=50

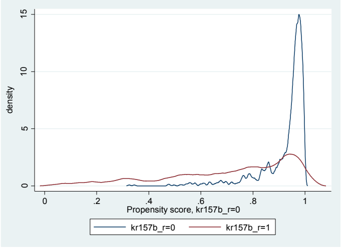

For peer review only - http://bmjopen.bmj.com/site/about/guidelines.xhtml

Table S4 (ii). Standardized differences on confounders after weighting

CONFOUNDERS	Standardized differences in weighted sample in model adjusted for child and family variables					Standardized differences in weighted sample in model adjusted for child and family variables and other parental strategies				
**Imputed dataset:**	**m=1**	**m=4**	**m=5**	**m=37**	**m=50**	**m=1**	**m=4**	**m=5**	**m=37**	**m=50**
**Child and family variables included as confounders:**										
Gender										
1. Female	*-.038*	*-.042*	*-.063*	*-.025*	*-.042*	*-.032*	*-.009*	*-.038*	*-.011*	*-.037*
Temperament - Activity at 24 m										
1. High score >28	*.018*	*.049*	*.015*	*-.004*	*.020*	*.022*	*.044*	*-.002*	*.001*	*.030*
Temperament - Adaptability at 24 m										
1. High score >17	*-.015*	*.024*	*-.012*	*-.047*	*-.022*	*-.008*	*.015*	*-.019*	*-.060*	*-.021*
Temperament - Mood at 24 m										
1. High score >24	*-.021*	*-.020*	*.004*	*-.028*	*-.023*	*-.063*	*-.080*	*-.025*	*-.070*	*-.050*
Hyperactivity score at 81 m										
1. High score>6	*.025*	*.066*	*.025*	*.012*	*.024*	*.018*	*.033*	*-.009*	*-.006*	*.017*
Emotional symptoms score at 81 m										
1. High score >=4	*-.002*	*.006*	*.007*	*-.020*	*.026*	*.004*	*.015*	*.000*	*-.007*	*.015*
Conduct problems score at 81 m										
1. High score >=4	*.052*	*-.003*	*.037*	*.030*	*.040*	*.059*	*.026*	*.062*	*.044*	*.054*
Total behavioral problems at 81 m										
1. High score >=14	*.034*	*.004*	*-.007*	*-.002*	*.010*	*.039*	*.011*	*-.030*	*-.004*	*.036*
EAS temperament - activity at 69 m										
1. High >=24	*.051*	*.098*	*.047*	*.057*	*.078*	*.028*	*.064*	*.031*	*.043*	*.056*
EAS temperament -sociability at 69 m										
1. High score >=22	*.022*	*.038*	*-.013*	*.009*	*.035*	*.001*	*.042*	*-.016*	*.011*	*.015*
Verbal IQ at 8 y										
1. 80<IQ<=120	*-.004*	*-.026*	*-.008*	*-.012*	*.029*	*-.013*	*.011*	*.016*	*.031*	*.006*
2. IQ>120	*-.017*	*-.006*	*-.022*	*.015*	*-.004*	*-.041*	*-.060*	*-.049*	*-.032*	*-.023*

For peer review only - http://bmjopen.bmj.com/site/about/guidelines.xhtml

Performance IQ at 8 y										
1. 80<IQ<=120	*-.018*	*-.004*	*-.037*	*-.018*	*-.008*	*-.010*	*-.001*	*-.034*	*-.032*	*-.038*
2. IQ>120	*.009*	*-.003*	*-.007*	*.008*	*.031*	*.003*	*.002*	*-.009*	*.000*	*.011*
Full scale IQ at 8 y										
1. 80<IQ<=120	*-.007*	*.044*	*.021*	*-.008*	*.034*	*-.035*	*.037*	*.046*	*.006*	*-.007*
2. IQ>120	*-.010*	*-.026*	*-.028*	*-.002*	*.012*	*-.019*	*-.047*	*-.035*	*-.018*	*.003*
Social cognition										
1. High score>=8	*.006*	*.041*	*.052*	*.006*	*-.008*	*-.011*	*.028*	*.039*	*-.003*	*.023*
Stressful life events (child related) at 42 m										
1. 5 or more stress events	*.012*	*-.069*	*.034*	*.008*	*-.017*	*.016*	*-.069*	*.017*	*-.013*	*-.002*
Sleep problems at 42 m										
1. 6 sleep problems or more	*.042*	*.081*	*.078*	*.041*	*.047*	*.067*	*.119*	*.111*	*.082*	*.065*
Child's general health at 81 m										
1. Minor problems	*.019*	*-.030*	*-.023*	*.035*	*-.014*	*.023*	*-.040*	*-.038*	*.010*	*-.020*
2. Ill/unwell	*-.100*	*-.099*	*-.067*	*-.111*	*-.073*	*-.159*	*-.163*	*-.105*	*-.147*	*-.095*
Bedtime wetting severity at 7.5 y										
1. Severe	*.016*	*.065*	*.042*	*.051*	*.021*	*.058*	*.098*	*.089*	*.095*	*.076*
Daytime wetting at 7.5 y										
1. Yes	*.011*	*.003*	*.026*	*.011*	*.023*	*.065*	*.064*	*.081*	*.056*	*.098*
Child needs to dash to toilet to urinate at 7.5 y										
1. Yes, straight away	*-.007*	*-.001*	*-.005*	*.009*	*-.010*	*.014*	*.020*	*.002*	*.020*	*-.006*
Daytime soiling at 7.5 y										
1. Yes	*.015*	*.054*	*.042*	*.006*	*.060*	*.012*	*.034*	*.030*	*-.005*	*.066*
Night time soiling at 7.5 y										
1. Yes	*.039*	*.107*	*.040*	*.024*	*.063*	*.062*	*.131*	*.056*	*.069*	*.121*
Car access										
1. No car at household when child is 33 m	*.028*	*-.033*	*.083*	*.010*	*.062*	*.010*	*-.039*	*.063*	*.008*	*.053*
Affordability index for elementary goods (mother) at 85m										

For peer review only - http://bmjopen.bmj.com/site/about/guidelines.xhtml

1. Severe deprivation	*.006*	*.033*	*.013*	*.016*	*-.029*	*-.001*	*.021*	*.020*	*.019*	*-.029*
Social class - based on mother's occupation										
1. Managerial and technical	*.001*	*.006*	*.022*	*-.007*	*-.042*	*-.050*	*.027*	*.045*	*.002*	*-.020*
2. Skilled non-manual	*.001*	*-.027*	*-.003*	*.006*	*.008*	*.045*	*-.032*	*-.009*	*-.008*	*.009*
3. Skilled manual	*-.014*	*.000*	*.000*	*-.048*	*.033*	*-.038*	*.000*	*.000*	*-.086*	*-.002*
4. Partly skilled	*-.008*	*.014*	*.017*	*.018*	*.012*	*.020*	*.021*	*.004*	*.034*	*-.003*
5. Unskilled	*.032*	*.028*	*-.046*	*.020*	*.029*	*.024*	*.027*	*-.027*	*.051*	*.029*
Social class - based on father's occupation										
1. Managerial and technical	*-.020*	*-.079*	*-.013*	*-.015*	*-.039*	*-.048*	*-.106*	*-.028*	*-.027*	*-.034*
2. Skilled non-manual	*.008*	*.051*	*.030*	*.032*	*.043*	*.033*	*.043*	*.031*	*.066*	*.042*
3. Skilled manual	*-.001*	*.014*	*-.017*	*-.013*	*.003*	*-.019*	*-.028*	*-.024*	*-.051*	*-.029*
4. Partly skilled	*-.026*	*.007*	*.015*	*-.014*	*-.004*	*-.012*	*.097*	*.043*	*.010*	*.031*
5. Unskilled	*.032*	*.014*	*-.006*	*.014*	*-.008*	*.054*	*.007*	*-.007*	*.026*	*-.010*
Early parenthood										
1. Had child at age <19	*-.043*	*-.026*	*-.026*	*-.029*	*-.013*	*-.079*	*-.067*	*-.067*	*-.068*	*-.045*
Amount of help mother has in looking after child at 85 m										
1. Too little	*-.008*	*-.015*	*-.012*	*-.008*	*-.013*	*-.051*	*-.053*	*-.034*	*-.032*	*-.042*
Mother's general health at 73 m										
1. Mostly well & healthy	*.004*	*.090*	*.060*	*.029*	*.032*	*-.026*	*.033*	*.048*	*.003*	*.024*
2. Often unwell & hardly ever well	*-.025*	*-.002*	*-.059*	*-.027*	*-.081*	*-.004*	*.023*	*-.069*	*-.018*	*-.053*
Mother bedwetting history										
1. mother had a problem when older than 5yo	*.014*	*.043*	*.026*	*-.004*	*.016*	*-.021*	*-.010*	*.015*	*-.048*	*-.015*
Mother day time wetting history										
1. Mother had bedwetting after 5 y	*-.033*	*-.032*	*.019*	*-.011*	*.002*	*-.071*	*-.085*	*.024*	*-.039*	*-.013*
Toilet training when child 15m										
1. Just started	*.031*	*.021*	*-.014*	*.005*	*.018*	*.017*	*.011*	*.000*	*.009*	*.011*
2. For some time	*-.038*	*-.010*	*-.041*	*.001*	*-.093*	*-.064*	*-.018*	*-.038*	*-.004*	*-.088*
Rules about smoking in household										

For peer review only - http://bmjopen.bmj.com/site/about/guidelines.xhtml

1. Smoking allowed in some rooms	*-.031*	*.028*	*-.001*	*-.002*	*-.006*	*-.034*	*.023*	*.028*	*.011*	*.000*
2. Smoking allowed	*-.030*	*.021*	*.014*	*-.011*	*.003*	*-.051*	*.000*	*.003*	*-.021*	*-.005*
Father figure is natural father of child										
1. No / not living with father figure	*.044*	*.023*	*.022*	*.023*	*.015*	*-.004*	*.008*	*.026*	*.029*	*.006*
**Related parental strategies variables (those with correlations > .45):**										
Restricting drinks										
1. Yes						*.104*	*.152*	*.133*	*.101*	*.146*

For peer review only

Table S5 (i). Diagnostics for **REWARDING**

**N in unweighted dataset: treated = 169; untreated 1,089**	**Model adjusted for child and family variables**					**Model adjusted for child and family variables and other parental strategies**				
	**m=1**	**m=4**	**m=5**	**m=37**	**m=50**	**m=1**	**m=4**	**m=5**	**m=37**	**m=50**
**N of treated in weighted dataset:**	**635**	**629**	**627**	**629**	**631**	**615**	**619**	**612**	**606**	**610**
**N of untreated in weighted dataset:**	**623**	**629**	**631**	**629**	**627**	**643**	**639**	**646**	**652**	**647**

Figure S5. Overlap for models for **REWARDING** including child and family variables

m=1

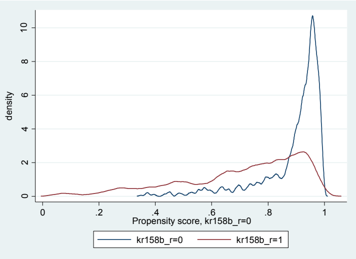

m=4

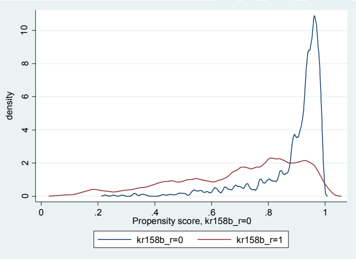

m=5

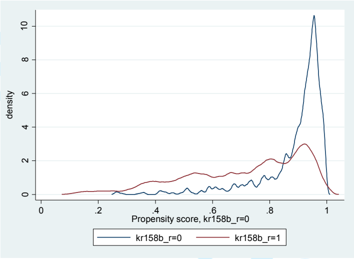

m=37

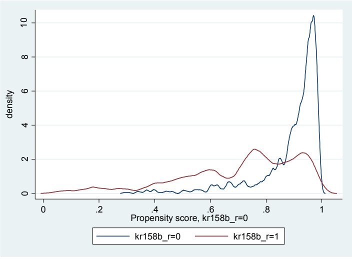

m=50

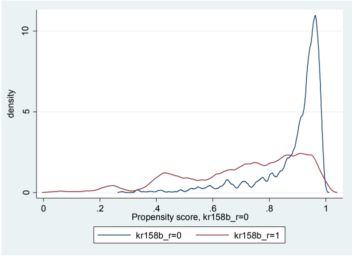

Overlap for models for **REWARDING** including child and family variables & other related parental strategies

m=1

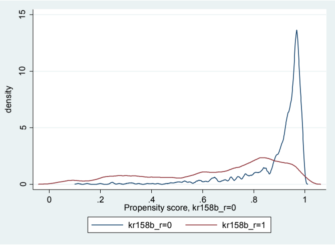

m=4

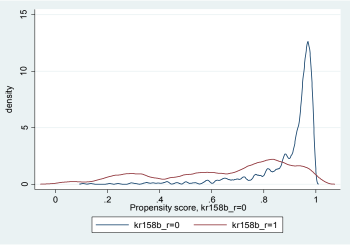

m=5

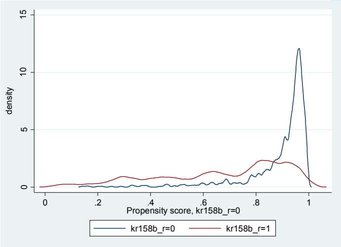

m=37

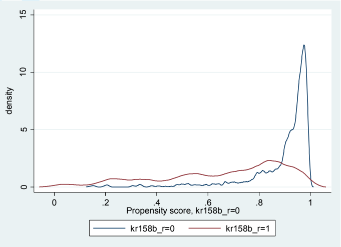

m=50

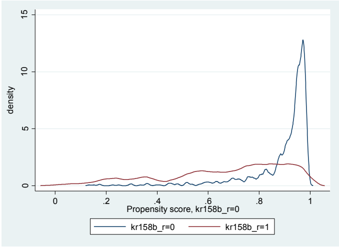

For peer review only - http://bmjopen.bmj.com/site/about/guidelines.xhtml

Table S5 (ii). Standardized differences on confounders after weighting

CONFOUNDERS	Standardized differences in weighted sample in model adjusted for child and family variables					Standardized differences in weighted sample in model adjusted for child and family variables and other parental strategies				
**Imputed dataset:**	**m=1**	**m=4**	**m=5**	**m=37**	**m=50**	**m=1**	**m=4**	**m=5**	**m=37**	**m=50**
**Child and family variables included as confounders:**										
Gender										
1. Female	*-.071*	*-.070*	*-.070*	*-.038*	*-.066*	*-.043*	*-.006*	*-.020*	*-.011*	*.004*
Temperament - Activity at 24 m										
1. High score >28	*.019*	*-.008*	*-.008*	*-.019*	*-.004*	*.032*	*.014*	*.006*	*-.011*	*.010*
Temperament - Mood at 24 m										
1. high score >24	*.040*	*.046*	*.046*	*.033*	*.050*	*.096*	*.030*	*.048*	*.067*	*.033*
Hyperactivity score at 81 m										
1. High score>6	*.034*	*-.023*	*-.023*	*.009*	*.019*	*.125*	*.030*	*.013*	*.036*	*.033*
Emotional symptoms score at 81 m										
1. High score >=4	*.006*	*-.062*	*-.061*	*-.061*	*-.026*	*-.040*	*.032*	*-.070*	*-.001*	*-.098*
Conduct problems score at 81 m										
1. High score >=4	*-.038*	*.005*	*.005*	*-.040*	*.005*	*-.008*	*.068*	*.098*	*.028*	*.082*
Total behavioral problems at 81 m										
1. High score >=14	*.014*	*-.053*	*-.053*	*-.044*	*.011*	*.054*	*.061*	*.014*	*.055*	*.081*
EAS temperament - activity at 69 m										
1 abnormal (score >=24 (hyper active))	*.067*	*.050*	*.050*	*.077*	*.073*	*.125*	*.106*	*.095*	*.125*	*.091*
EAS temperament -sociability at 69 m										
1. High score >=22	*-.007*	*-.019*	*-.019*	*.013*	*.022*	*.097*	*.049*	*.037*	*.078*	*.036*
Verbal IQ at 8 y										
1. 80<IQ<=120	*.049*	*-.010*	*-.010*	*-.026*	*.010*	*.018*	*.138*	*.045*	*-.029*	*.027*
2. IQ>120	*-.028*	*.030*	*.030*	*.028*	*-.009*	*-.046*	*-.123*	*-.049*	*.012*	*-.061*
Performance IQ at 8 y										

For peer review only - http://bmjopen.bmj.com/site/about/guidelines.xhtml

1. 80<IQ<=120	*.014*	*-.023*	*-.023*	*.042*	*-.047*	*.007*	*.066*	*-.026*	*.036*	*-.082*
2. IQ>120	*-.016*	*.004*	*.004*	*-.007*	*.009*	*-.142*	*-.018*	*-.068*	*-.075*	*-.055*
Full scale IQ at 8 y										
1. 80<IQ<=120	*.032*	*.004*	*.004*	*.040*	*.003*	*-.001*	*.023*	*.074*	*.076*	*.017*
2. IQ>120	*.001*	*.032*	*.032*	*-.002*	*.017*	*-.055*	*.008*	*-.088*	*-.044*	*-.069*
Social cognition										
1. Score>=8	*.041*	*-.016*	*-.016*	*-.005*	*.007*	*.049*	*.057*	*.028*	*.070*	*.036*
Stressful life events (child related) at 42 m										
1. 5 or more stress events	*-.013*	*-.005*	*-.005*	*.012*	*-.021*	*.031*	*-.035*	*-.013*	*.006*	*-.019*
Bedwetting severity										
1. Severe	*-.018*	*-.018*	*-.018*	*-.010*	*.004*	*-.053*	*-.065*	*-.071*	*-.093*	*-.060*
Daytime wetting										
1. Yes	*-.001*	*-.019*	*-.019*	*.008*	*-.010*	*.003*	*.081*	*.057*	*.057*	*.017*
Child goes to toilet without reminder at 7.5 y										
1. Never	*.000*	*.002*	*.002*	*-.003*	*.000*	*-.012*	*-.008*	*-.024*	*-.002*	*-.005*
Night time soiling at 7.5 y										
1. Yes	*.057*	*.039*	*.039*	*.005*	*-.007*	*.068*	*.082*	*.084*	*.081*	*-.006*
Affordability index for elementary goods (mother) at 85m										
1. Severe deprivation	*.032*	*.006*	*.006*	*-.025*	*.012*	*.099*	*.089*	*.068*	*.010*	*.076*
Social class - based on mother's occupation										
1. Managerial and technical	*.047*	*.008*	*.008*	*-.004*	*-.019*	*.002*	*.116*	*.085*	*.033*	*-.003*
2. Skilled non-manual	*-.057*	*-.023*	*-.023*	*-.010*	*-.026*	*-.078*	*-.169*	*-.143*	*-.053*	*-.077*
3. Skilled manual	*.071*	*-.018*	*-.018*	*-.025*	*.049*	*.042*	*.069*	*.012*	*-.063*	*-.001*
4. Partly skilled	*.002*	*.018*	*.018*	*.010*	*.003*	*.024*	*.021*	*.039*	*.034*	*.049*
5. Unskilled	*-.032*	*.003*	*.003*	*.043*	*.059*	*.073*	*.045*	*.036*	*.059*	*.086*
Social class - based on father's occupation										
1. Managerial and technical	*-.035*	*-.033*	*-.033*	*-.051*	*-.013*	*-.071*	*-.055*	*-.097*	*-.054*	*-.009*

For peer review only - http://bmjopen.bmj.com/site/about/guidelines.xhtml

2. Skilled non-manual	.054	.038	.038	.035	.061	.083	.057	.087	.113	.096
3. Skilled manual	.030	.011	.011	.029	-.019	-.028	.045	.013	-.039	-.089
4. Partly skilled	-.028	-.020	-.020	.000	.007	.005	-.044	-.008	-.021	.026
5. Unskilled	-.006	.018	.018	.010	.000	.067	.007	.045	.050	.021
Early parenthood										
1. Had child at age <19	.011	.024	.024	.027	.044	.039	.035	.047	.057	.067
Amount of help mother has in looking after child at 85m										
1. Too little	.038	.069	.069	-.002	.022	.106	.125	.155	.131	.134
Mother thinks she spends enough time with child (85m)										
1. No	.010	.012	.012	.025	.032	.009	.027	-.027	.032	.011
Mother's state of health 73m										
1. Mostly well & healthy	.062	.023	.023	.040	.040	.090	-.034	.024	.033	.063
2. Often unwell & hardly ever well	-.048	-.056	-.056	-.032	-.027	.034	.022	-.067	.041	.049
Mother bedwetting history										
1. Mother had bedwetting after 5 y	.011	-.002	-.002	.037	.031	.058	-.034	.011	.050	.044
Mother's daytime wetting history										
1. Mother had daytime wetting after 5 y	.002	-.056	-.056	-.009	.013	.052	.059	.009	.055	.073
Toilet training when child 15m										
1. Just started	-.024	-.028	-.028	.009	.009	.065	-.048	.011	.023	.010
2. For some time	.005	.000	.000	.010	.024	.006	.023	.026	.042	.047
**Related parental strategies variables (those with correlations > .45):**										
Restricting drinks										
1. Yes						-.081	-.067	-.051	-.099	-.097
Alarm prevention										
1. Yes						-.219	-.098	-.202	-.168	-.187

For peer review only - http://bmjopen.bmj.com/site/about/guidelines.xhtml

Table S6 (i). Diagnostics for **SHOWING DISPLEASURE**

N in unweighted dataset: treated = 55; untreated 1,203	Model adjusted for child and family variables					Model adjusted for child and family variables and other parental strategies				
	m=1	m=4	m=5	m=37	m=50	m=1	m=4	m=5	m=37	m=50
N of treated in weighted dataset:	625	627	631	623	626	629	625	628	619	622
N of untreated in weighted dataset:	633	631	628	635	631	629	633	630	639	636

Figure S6. Overlap for models for **SHOWING DISPLEASURE** including child and family variables

m=1

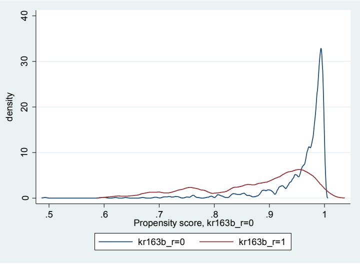

m=4

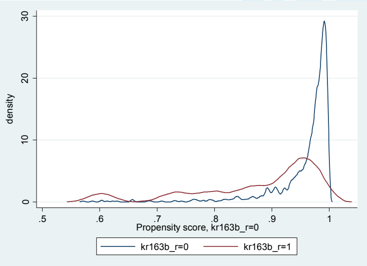

m=5

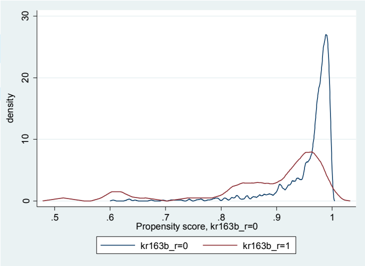

m=37

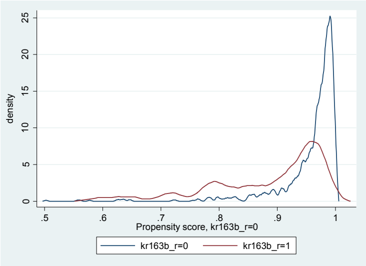

m=50

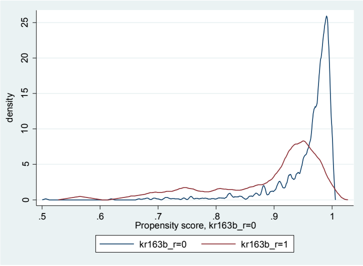

Overlap for models for **SHOWING DISPLEASURE** including child and family variables & other related parental strategies

m=1

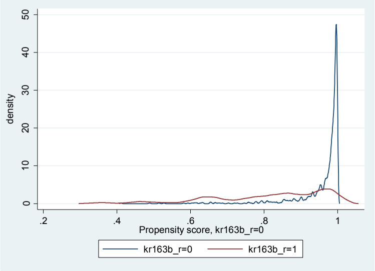

m=4

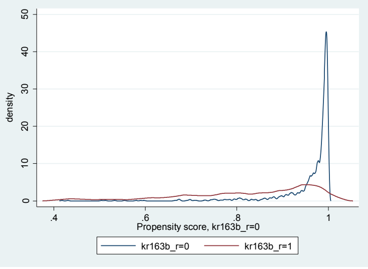

m=5

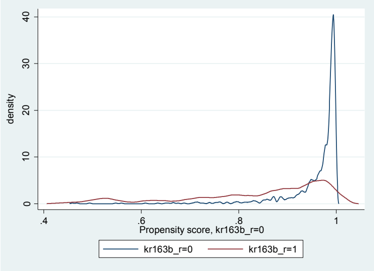

m=37

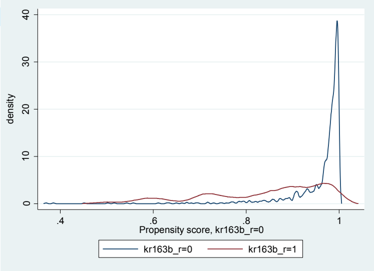

m=50

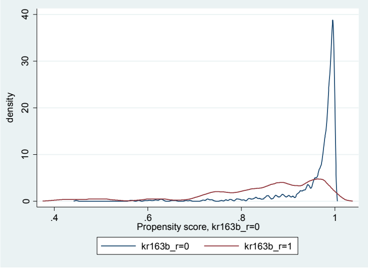

For peer review only - http://bmjopen.bmj.com/site/about/guidelines.xhtml

Table S6 (ii). Standardized differences on confounders after weighting

CONFOUNDERS	Standardized differences in weighted sample in model adjusted for child and family variables					Standardized differences in weighted sample in model adjusted for child and family variables and other parental strategies				
**Imputed dataset:**	**m=1**	**m=4**	**m=5**	**m=37**	**m=50**	**m=1**	**m=4**	**m=5**	**m=37**	**m=50**
**Child and family variables included as confounders:**										
Gender										
1. Female	*.013*	*.043*	*.030*	*.020*	*.034*	*-.004*	*.049*	*.031*	*-.006*	*.020*
Temperament - Mood at 24 m										
1. High score >24	*-.029*	*-.010*	*-.013*	*.000*	*-.009*	*.009*	*.015*	*.001*	*.026*	*.008*
Hyperactivity score at 81 m										
1. High score>6	*.003*	*.006*	*.009*	*-.010*	*.002*	*-.027*	*-.029*	*-.012*	*-.049*	*.007*
Emotional symptoms score at 81 m										
1. High score >=4	*-.010*	*.000*	*-.014*	*.010*	*-.003*	*-.037*	*-.029*	*-.050*	*-.001*	*-.012*
Conduct problems score at 81 m										
1. High score >=4	*.033*	*.044*	*.037*	*.013*	*.030*	*.009*	*.046*	*.044*	*-.011*	*.035*
Total behavioral problems at 81 m										
1. High score >=14	*.008*	*.011*	*.015*	*.003*	*-.008*	*-.025*	*-.023*	*.006*	*-.028*	*-.010*
EAS temperament - activity at 69 m										
1. High score >=24	*.014*	*-.007*	*.002*	*.016*	*-.007*	*.021*	*-.023*	*-.021*	*.011*	*-.010*
EAS temperament -sociability at 69m										
1. High score >=22	*.037*	*.048*	*.045*	*.060*	*.060*	*.062*	*.076*	*.063*	*.095*	*.098*
Verbal IQ ay 8 y										
1. 80<IQ<=120	*-.001*	*.013*	*.000*	*-.012*	*-.007*	*-.011*	*-.016*	*-.015*	*-.044*	*-.014*
2. IQ>120	*-.011*	*-.014*	*-.012*	*.008*	*.003*	*-.005*	*.010*	*.004*	*.034*	*.009*
Performance IQ at 8 y										
1. 80<IQ<=120	*.020*	*-.013*	*.016*	*.005*	*-.007*	*.025*	*-.048*	*.008*	*-.007*	*-.002*
2. IQ>120	*-.035*	*-.031*	*-.032*	*-.015*	*-.017*	*-.028*	*-.017*	*-.022*	*.012*	*-.017*
Full scale IQ at 8 y										

For peer review only

For peer review only - http://bmjopen.bmj.com/site/about/guidelines.xhtml

1. 80<IQ<=120	*.018*	*.016*	*.018*	*.006*	*.004*	*.006*	*-.014*	*-.008*	*-.034*	*-.005*
2. IQ>120	*-.021*	*-.029*	*-.019*	*-.007*	*-.010*	*.002*	*.004*	*.003*	*.044*	*-.001*
Social cognition										
1.(score>=8)	*-.001*	*.013*	*-.010*	*.018*	*-.007*	*.004*	*.024*	*.000*	*.031*	*.006*
Stressful life events (child related) at 42 m										
1. 5 or more stress events	*-.034*	*-.008*	*-.009*	*-.019*	*-.025*	*.006*	*.011*	*-.022*	*-.057*	*-.001*
Sleep problems as 42 m										
1. 6 sleep problems or more	*-.005*	*.001*	*-.033*	*-.025*	*.000*	*-.040*	*-.018*	*-.062*	*-.083*	*-.030*
Child's general health at 81 m										
1. Minor problems	*.007*	*-.006*	*.017*	*.014*	*.009*	*.001*	*.005*	*.024*	*.043*	*-.012*
2. Ill/unwell	*-.058*	*-.042*	*-.032*	*-.060*	*-.057*	*-.040*	*-.049*	*-.006*	*-.064*	*-.011*
Child goes to toilet without reminder at 7.5 y										
1. Never	*-.033*	*-.011*	*-.027*	*-.016*	*.002*	*-.072*	*-.038*	*-.020*	*-.032*	*-.007*
Bedwetting severity at 7.5 y										
1. Severe	*.001*	*-.038*	*-.013*	*-.003*	*-.015*	*.005*	*-.058*	*-.016*	*-.002*	*-.029*
Daytime wetting at 7.5 y										
1. Yes	*-.025*	*-.018*	*.011*	*-.018*	*-.014*	*-.036*	*-.011*	*-.019*	*-.052*	*-.035*
Family size										
1. 3 or more children	*.003*	*-.002*	*-.001*	*-.028*	*-.026*	*.068*	*.014*	*.037*	*.034*	*-.009*
Affordability index for elementary goods (mother) at 85m										
1. Severe deprivation	*.002*	*.008*	*.011*	*.014*	*.016*	*.031*	*.051*	*.021*	*.041*	*.008*
Social class - based on mother's occupation										
1. Managerial and technical	*-.017*	*-.033*	*-.038*	*-.021*	*-.016*	*-.012*	*-.052*	*-.035*	*-.057*	*.005*
2. Skilled non-manual	*.014*	*.022*	*.011*	*.000*	*-.006*	*-.003*	*-.013*	*.002*	*-.005*	*-.024*
3. Skilled manual	*.031*	*.029*	*.037*	*.014*	*.018*	*.025*	*.028*	*.039*	*.005*	*.011*
4. Partly skilled	*-.001*	*.021*	*-.007*	*.019*	*.005*	*-.009*	*.032*	*-.005*	*.074*	*.008*
5. Unskilled	*.016*	*-.014*	*.048*	*.003*	*.033*	*.007*	*-.004*	*.064*	*.009*	*.035*
Social class - based on father's										

For peer review only

For peer review only - http://bmjopen.bmj.com/site/about/guidelines.xhtml

occupation										
1. Managerial and technical	-.001	-.012	-.019	.007	.001	.025	.019	.017	.024	.015
2. Skilled non-manual	.014	-.016	-.006	-.001	-.015	.028	.015	.007	.027	.004
3. Skilled manual	-.025	.008	.007	.006	-.008	-.073	-.044	-.036	-.042	-.031
4. Partly skilled	.018	.027	.052	-.004	.030	.042	.026	.053	.007	.028
5. Unskilled	.027	.004	.000	.000	.000	.023	.017	.005	.000	.000
Early parenthood										
1. Had child at age <19	.000	-.023	.008	-.011	.000	.047	-.041	.024	.028	.035
Amount of help mother has in looking after child at 85 m										
1. Too little	-.024	-.006	-.007	-.035	-.021	-.033	-.012	.030	-.048	.006
Mother's general health 73 m										
1. Mostly well & healthy	-.020	-.009	-.023	-.011	-.015	-.044	-.050	-.043	-.010	-.012
2. Often unwell & hardly ever well	.010	.029	.009	.023	-.006	.034	.063	.025	.055	.024
Mother's daytime wetting history										
1. Mother had daytime wetting after 5 y	.004	.018	.005	-.018	.029	.010	.016	-.001	-.005	.046
Toilet training when child 15m										
1. Just started	.034	.037	.034	.026	.029	.013	.042	.026	.014	.025
2 .For some time	.000	-.016	.007	-.004	.017	.061	.015	.040	.066	.057
Rules about smoking in household										
1. Smoking allowed in some rooms	.021	.016	.002	.011	.020	.014	-.014	-.006	-.010	.023
2. Smoking allowed	-.003	-.007	-.020	-.031	-.052	-.029	.002	-.044	-.043	-.052
Father figure is natural father of child										
1. No / not living with father figure	.020	.017	-.001	.008	.027	.035	-.004	-.041	.024	-.007
**Related parental strategies variables (those with correlations > .45):**										
Restricting drinks										
1.yes						.023	.003	.008	-.014	-.007

For peer review only

Table S7 (i). Diagnostics for **PROTECTION PANTS**

**N in unweighted dataset: treated = 98; untreated =1,160**	**Model adjusted for child and family variables**				
	**m=1**	**m=4**	**m=5**	**m=37**	**m=50**
**N of treated in weighted dataset:**	**637**	**638**	**629**	**621**	**626**
**N of untreated in weighted dataset:**	**621**	**620**	**629**	**637**	**632**

Figure S7. Overlap for models for **PROTECTION PANTS** including child and family variables

m=1	m=4	m=5	m=37	m=50
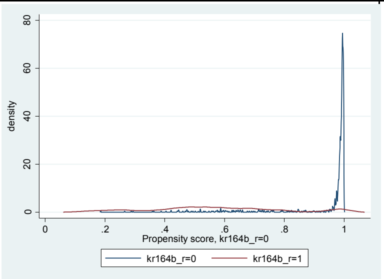	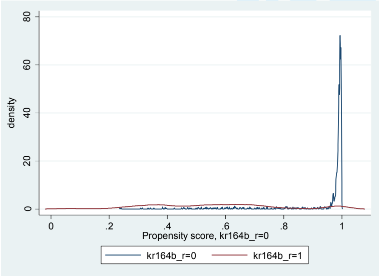	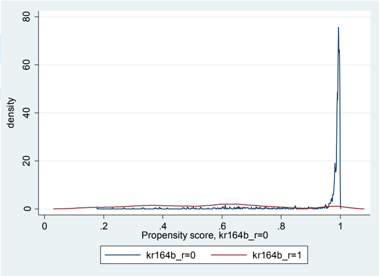	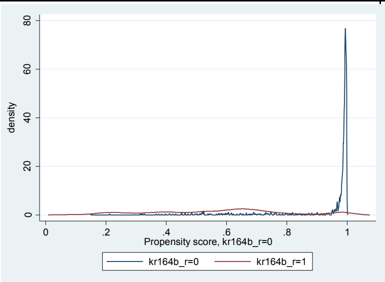	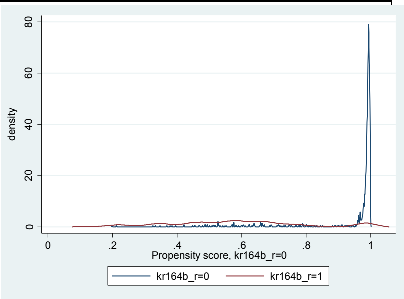

No other strategy was correlated with Protection pants strategy above the agreed level. Therefore in this case the above table includes only one row of figures.

For peer review only

Table S7 (ii). Standardized differences on confounders after weighting

CONFOUNDERS	Standardized differences in weighted sample in model adjusted for child and family variables				
**Imputed dataset:**	**m=1**	**m=4**	**m=5**	**m=37**	**m=50**
**Child and family variables included as confounders:**					
Gender					
1. Female	*.042*	*.028*	*.087*	*.041*	*.086*
Temperament - Adaptability at 24 m					
1. High score >17	*.000*	*.036*	*.053*	*.010*	*.021*
Temperament - Mood at 24 m					
1. High score >24	*-.030*	*-.048*	*-.013*	*.007*	*-.011*
Conduct problems score at 81 m					
1. High score >=4	*-.014*	*.010*	*-.022*	*.037*	*.058*
Total behavioral problems at 81 m					
1. High score >=14	*-.067*	*-.005*	*-.085*	*.048*	*.015*
EAS temperament - activity at 69 m					
1. High score >=24	*.015*	*-.018*	*-.006*	*.039*	*-.013*
EAS temperament -sociability at 69 m					
1. High score >= 22	*.038*	*.058*	*.052*	*-.043*	*.014*
Social cognition					
1. Score>=8	*-.025*	*-.008*	*-.013*	*-.014*	*-.009*
Full scale IQ at 8 y					
1. 80<IQ<=120	*-.016*	*-.059*	*.005*	*.072*	*.022*
2. IQ>120	*.032*	*.116*	*.037*	*.006*	*.078*
Verbal IQ at 8 y					
1. 80<IQ<=120	*-.090*	*-.141*	*-.074*	*-.065*	*-.086*
2. IQ>120	*.058*	*.130*	*.057*	*.053*	*.109*
Performance IQ at 8 y					
1. 80<IQ<=120	*-.139*	*-.176*	*-.130*	*.016*	*-.002*
2. IQ>120	*.072*	*.201*	*.142*	*.037*	*.086*

For peer review only - http://bmjopen.bmj.com/site/about/guidelines.xhtml

Stressful life events (child related) at 42 m					
1. 5 or more stressful events	*.041*	*-.008*	*.030*	*-.039*	*-.034*
Sleep problems as 42 m					
1. 6 sleep problems or more	*.001*	*.000*	*.019*	*.026*	*-.008*
Child's general health at 81m					
1. Minor problems	*.029*	*.012*	*.006*	*.093*	*.056*
2. Ill/unwell	*-.007*	*-.057*	*.040*	*-.010*	*.011*
Bedwetting severity at 7.5 y					
1. Severe	*.010*	*.012*	*.000*	*-.012*	*-.004*
Daytime wetting at 7.5 y					
1. Yes	*.024*	*.022*	*.041*	*-.022*	*-.022*
Daytime soiling					
1. Yes	*-.058*	*-.087*	*-.043*	*-.030*	*-.007*
Child needs to dash to toilet to urinate at 7.5 y					
1. Yes, straight away	*-.005*	*-.031*	*.002*	*.019*	*-.020*
Affordability index for elementary goods (mother) at 85m					
1. Severe deprivation	*.055*	*.071*	*.088*	*.048*	*.074*
Rules about smoking in household					
1. Smoking allowed in some rooms	*.039*	*.007*	*-.044*	*.054*	*-.002*
2. Smoking allowed	*.072*	*.060*	*.053*	*.055*	*.048*
Father figure is natural father of child					
1. no / not living with father figure	*-.008*	*-.015*	*-.014*	*-.005*	*.001*
**Related parental strategies variables (those with correlations > .45):**					
None					

For peer review only - http://bmjopen.bmj.com/site/about/guidelines.xhtml

STROBE 2007 (v4) Statement—Checklist of items that should be included in reports of *cohort studies*

Section/Topic	Item #	Recommendation	Reported on page #
**Title and abstract**	1	(*a*) Indicate the study's design with a commonly used term in the title or the abstract	1
		(*b*) Provide in the abstract an informative and balanced summary of what was done and what was found	2
**Introduction**			
Background/rationale	2	Explain the scientific background and rationale for the investigation being reported	4-5
Objectives	3	State specific objectives, including any prespecified hypotheses	5
**Methods**			
Study design	4	Present key elements of study design early in the paper	6
Setting	5	Describe the setting, locations, and relevant dates, including periods of recruitment, exposure, follow-up, and data collection	6
Participants	6	(*a*) Give the eligibility criteria, and the sources and methods of selection of participants. Describe methods of follow-up	6
		(*b*) For matched studies, give matching criteria and number of exposed and unexposed	
Variables	7	Clearly define all outcomes, exposures, predictors, potential confounders, and effect modifiers. Give diagnostic criteria, if applicable	6, 10-11, & table S1
Data sources/ measurement	8*	For each variable of interest, give sources of data and details of methods of assessment (measurement). Describe comparability of assessment methods if there is more than one group	6, 10, table S1
Bias	9	Describe any efforts to address potential sources of bias	12-14 & supplementary materials
Study size	10	Explain how the study size was arrived at	6-7
Quantitative variables	11	Explain how quantitative variables were handled in the analyses. If applicable, describe which groupings were chosen and why	12-14
Statistical methods	12	(*a*) Describe all statistical methods, including those used to control for confounding	12-14 & supplementary materials
		(*b*) Describe any methods used to examine subgroups and interactions	

For peer review only

		(*c*) Explain how missing data were addressed	14 & supplementary materials
		(*d*) If applicable, explain how loss to follow-up was addressed	14 & supplementary materials
		(*e*) Describe any sensitivity analyses	14 & supplementary materials
**Results**			
Participants	13*	(a) Report numbers of individuals at each stage of study—eg numbers potentially eligible, examined for eligibility, confirmed eligible, included in the study, completing follow-up, and analysed	6-7
		(b) Give reasons for non-participation at each stage	6-7
		(c) Consider use of a flow diagram	
Descriptive data	14*	(a) Give characteristics of study participants (eg demographic, clinical, social) and information on exposures and potential confounders	8-9
		(b) Indicate number of participants with missing data for each variable of interest	14 & table S1
		(c) Summarise follow-up time (eg, average and total amount)	
Outcome data	15*	Report numbers of outcome events or summary measures over time	
Main results	16	(*a*) Give unadjusted estimates and, if applicable, confounder-adjusted estimates and their precision (eg, 95% confidence interval). Make clear which confounders were adjusted for and why they were included	16
		(*b*) Report category boundaries when continuous variables were categorized	
		(*c*) If relevant, consider translating estimates of relative risk into absolute risk for a meaningful time period	
Other analyses	17	Report other analyses done—eg analyses of subgroups and interactions, and sensitivity analyses	Supplementary materials (p. 3-4)
**Discussion**			
Key results	18	Summarise key results with reference to study objectives	19
**Limitations**			
Interpretation	20	Give a cautious overall interpretation of results considering objectives, limitations, multiplicity of analyses, results from similar studies, and other relevant evidence	19-23
Generalisability	21	Discuss the generalisability (external validity) of the study results	24
**Other information**			
Funding	22	Give the source of funding and the role of the funders for the present study and, if applicable, for the original study on	24

For peer review only

		which the present article is based	

*Give information separately for cases and controls in case-control studies and, if applicable, for exposed and unexposed groups in cohort and cross-sectional studies.

Note: An Explanation and Elaboration article discusses each checklist item and gives methodological background and published examples of transparent reporting. The STROBE checklist is best used in conjunction with this article (freely available on the Web sites of PLoS Medicine at http://www.plosmedicine.org/, Annals of Internal Medicine at http://www.annals.org/, and Epidemiology at http://www.epidem.com/). Information on the STROBE Initiative is available at www.strobe-statement.org.

For peer review only
